# Deep-Sea Biodiversity in the Mediterranean Sea: The Known, the Unknown, and the Unknowable

**DOI:** 10.1371/journal.pone.0011832

**Published:** 2010-08-02

**Authors:** Roberto Danovaro, Joan Batista Company, Cinzia Corinaldesi, Gianfranco D'Onghia, Bella Galil, Cristina Gambi, Andrew J. Gooday, Nikolaos Lampadariou, Gian Marco Luna, Caterina Morigi, Karine Olu, Paraskevi Polymenakou, Eva Ramirez-Llodra, Anna Sabbatini, Francesc Sardà, Myriam Sibuet, Anastasios Tselepides

**Affiliations:** 1 Dipartimento Scienze del Mare, Università Politecnica delle Marche, Ancona, Italy; 2 Institut de Ciències del Mar, Consejo Superior de Investigaciones Científicas, Barcelona, Spain; 3 Department of Animal and Environmental Biology, University of Bari, Bari, Italy; 4 National Institute of Oceanography, Israel Oceanographic and Limnological Research, Haifa, Israel; 5 National Oceanography Centre, Southampton, United Kingdom; 6 Hellenic Center for Marine Research, Crete, Greece; 7 Stratigraphy Department, Geological Survey of Denmark and Greenland, Copenhagen, Denmark; 8 Département Etude des Ecosystèmes Profonds, Ifremer Centre de Brest, Plouzané, France; 9 Institut Océanographique, Paris, France; 10 Department of Maritime Studies, University of Piraeus, Piraeus, Greece; Smithsonian's National Zoological Park, United States of America

## Abstract

Deep-sea ecosystems represent the largest biome of the global biosphere, but knowledge of their biodiversity is still scant. The Mediterranean basin has been proposed as a hot spot of terrestrial and coastal marine biodiversity but has been supposed to be impoverished of deep-sea species richness. We summarized all available information on benthic biodiversity (Prokaryotes, Foraminifera, Meiofauna, Macrofauna, and Megafauna) in different deep-sea ecosystems of the Mediterranean Sea (200 to more than 4,000 m depth), including open slopes, deep basins, canyons, cold seeps, seamounts, deep-water corals and deep-hypersaline anoxic basins and analyzed overall longitudinal and bathymetric patterns. We show that in contrast to what was expected from the sharp decrease in organic carbon fluxes and reduced faunal abundance, the deep-sea biodiversity of both the eastern and the western basins of the Mediterranean Sea is similarly high. All of the biodiversity components, except Bacteria and Archaea, displayed a decreasing pattern with increasing water depth, but to a different extent for each component. Unlike patterns observed for faunal abundance, highest negative values of the slopes of the biodiversity patterns were observed for Meiofauna, followed by Macrofauna and Megafauna. Comparison of the biodiversity associated with open slopes, deep basins, canyons, and deep-water corals showed that the deep basins were the least diverse. Rarefaction curves allowed us to estimate the expected number of species for each benthic component in different bathymetric ranges. A large fraction of exclusive species was associated with each specific habitat or ecosystem. Thus, each deep-sea ecosystem contributes significantly to overall biodiversity. From theoretical extrapolations we estimate that the overall deep-sea Mediterranean biodiversity (excluding prokaryotes) reaches approximately 2805 species of which about 66% is still undiscovered. Among the biotic components investigated (Prokaryotes excluded), most of the unknown species are within the phylum Nematoda, followed by Foraminifera, but an important fraction of macrofaunal and megafaunal species also remains unknown. Data reported here provide new insights into the patterns of biodiversity in the deep-sea Mediterranean and new clues for future investigations aimed at identifying the factors controlling and threatening deep-sea biodiversity.

## Introduction

Deep-sea ecosystems include the waters and sediments beneath approximately 200 m depth. They represent the world's largest biome, covering more than 65% of the earth's surface and including more than 95% of the global biosphere. Despite their huge dimensions, our knowledge of both pelagic and benthic deep-sea diversity is scant [1], [2]. In the last decades, an increasing number of studies have been conducted to investigate deep-sea biodiversity in several regions of the world, including the Atlantic and mid-Atlantic ocean [3], [4], the Arabian Sea [3], [5]–[9], and the equatorial, tropical, and subtropical Pacific. But these studies focus on a limited number of taxa and are typically characterized by a limited spatial or temporal scale of investigation [7], [8], [10]–[12]. Traditionally the Mediterranean Sea is one of the most intensively investigated areas of the world in both terrestrial and coastal marine biodiversity, but it lags other regions of the world in studies of its deep-sea fauna.

The Mediterranean Sea is divided into western and central-eastern basins, which are separated by the Strait of Sicily. The western basin (mean depth, about 1,600 m) consists of two deep basins: the Algero Provençal basin and the Tyrrhenian Sea. The central-eastern Mediterranean consists of three main deep basins: the Ionian, Aegean, and Levantine [13]. The deepest point in the Mediterranean, 5,121 m, is found at the North Matapan-Vavilov Trench, Ionian Sea [14]. The deep-sea floor includes regions characterized by complex sedimentological and structural features: (a) continental slopes, (b) submarine canyons, (c) base-of-slope deposits, and (d) bathyal or basin plains with abundant deposits of hemipelagic and turbidity muds. Sedimentological and stratigraphic features that contribute to the complexity of the deep-sea basin include (a) effects of the Messinian salinity crisis, with the creation of deep-hypersaline anoxic basins, (b) cold seepage and “mud volcanism” associated with the release of gas from deep-sea sediments, (c) the role of catastrophic events (e.g., landslides), which increase considerably the topographic complexity of the seafloor, and (d) volcanism and its influence on the topographic features and the creation of seamounts. Water circulation is highly complex. The surface waters come from the Atlantic and turn into intermediate waters in the Eastern Mediterranean. Low-salinity Atlantic waters enter the Mediterranean, while denser deep-Mediterranean waters flow beneath the Atlantic waters in the opposite direction into the Atlantic Ocean. Mesoscale variability is extremely evident in the Mediterranean and is responsible for the creation of small gyres (eddies) that have implications for the primary productivity and the flux of organic matter settling to the seafloor. Deep and bottom currents are largely unexplored, but episodic intensification of current speed up to 1 m s^−1^ has been documented [15]. During late spring and summer, the whole Western Mediterranean is strongly stratified, the seasonal thermocline being 20–50 m deep. In winter, the water column is more homogeneous, especially in the open sea. High oxygen concentrations are present across the water column down to the seafloor [16].

The main hydrological features of the deep Mediterranean Sea are (a) high homeothermy from roughly 300–500 m to the bottom, and bottom temperatures of about 12.8°C to 13.5°C in the western basin and 13.5°C to 15.5°C in the eastern basin (i.e., there are no thermal boundaries, whereas in the Atlantic Ocean the temperature decreases with depth) [17], (b) high salinity, from about 38 to 39.5 by the stratification of the water column, (c) limited freshwater inputs (the freshwater deficit is equivalent to about 0.5–0.9 m y^−1^, compensated by the Atlantic inflow of surface water), (d) a microtidal regime, (e) high oxygen concentrations, and (f) oligotrophic conditions, with strong energetic gradients and low nutrient concentrations in the eastern basin [18]. The eastern basin is considered to be one of the most oligotrophic areas of the world [19], [20] (see Text S1 for a full list of references). Inputs of organic carbon are 15–80 times lower than in the western basin and there are extremely low concentrations of chlorophyll-a in surface offshore waters (about 0.05 µg L^−1^) [21], [22]. In addition, there are low concentrations of the potentially limiting organic nutrients (e.g., proteins and lipids) that sharply decline with increasing distance from the coast and depth within the sediment. The average depth of the Mediterranean basin is about 1,450 m, much shallower than the average depth of the world oceans (about 3,850 m). This has several implications for the deep-water turnover (roughly 50 years) and the vulnerability to climate change and deep-water warming. The Mediterranean Sea has been considered a “miniature ocean” that can be used as a model to anticipate the response of the global oceans to various kinds of pressures.

The Mediterranean basin is a hot spot of biodiversity with a uniquely high percentage of endemic species [23]. Despite its small dimensions (0.82% of the ocean surface), the basin hosts more than 7.5% of global biodiversity [24]. However, this information is almost completely confined to coastal ecosystems, and data on deep-sea assemblages are still limited [25]–[27]. This is unfortunate, as pioneer investigations of macrobenthos were conducted in the deep Cretan Sea (see Text S1 for a full list of references). While dredging in the Aegean Sea, Forbes noticed that sediments became progressively more impoverished in biodiversity with increasing sampling depth, and Forbes proposed the azoic hypothesis [28], namely, that life would be extinguished altogether by 500 m depth [29]. The Forbes hypothesis was accepted as fact, despite indisputable evidence of the presence of deep-sea life from the Gulf of Genoa [30] (see Text S1 for a complete list of references) and at depths down to 1,000 m [31]. Benthic and benthopelagic deep-sea fauna in the Mediterranean (Tyrrhenian Sea) were provided by the *Washington* expedition (1881–83) with trawls carried out down to 3,115 m depths (see Text S1 for a complete list of references). After this exploration, knowledge of Mediterranean deep-sea fauna was mainly provided by the *Hirondelle* and *Princesse Alice* expeditions (1888–1922), the ichthyological results of which were reported by Zugmayer [32] (see Text S1 for a complete list of references). The most extensive deep-sea faunistic exploration in the Levant basin of the Mediterranean occurred during the voyages of the *Pola* (1890–93). The Danish oceanographic cruises of the *Thor* (1908) and *Dana* (1928–29) also reported deep-sea fish at depths greater than 1,000 m in the Mediterranean (see Text S1 for a complete list of references). After the Danish oceanographic expeditions, the first noteworthy sampling of deep-sea fish in the Mediterranean was during the Polymède campaign made with the RV *Jean Charcot* [33] in the western basin and the German *Meteor* expedition in the eastern basin [34]. During the second half of the twentieth century, little deep-sea sampling was conducted in the deep Mediterranean, providing scattered information on Macrofauna [35]–[37] (see Text S1 for a complete list of references). However, from the late 1980s, when specific projects were designed for systematic investigation of the deep sea below 1,000 m depth, several deep-sea benthic studies have been conducted in the Mediterranean Sea [13], [20], [38]–[49], including the deep Levantine Sea [50]–[53]. In this latter period, deep-sea trawls (Agassiz drags and otter trawls) and bottom long-lines were used [54] (see Text S1 for a complete list of references), allowing the collection of several megafaunal species, including four deep-water shark species at depths of 1,330–1,440 m [55]. The first investigations on deep-sea Meiofauna started in the Western Mediterranean and subsequently expanded to the entire basin [18], [56]–[68]. In 2001, a multidisciplinary trans-Mediterranean cruise investigated bathyal and abyssal (600–4,000 m) fauna, providing pioneer data on the distribution, biology, and ecology of Meio-, Macro-, and Megafauna [46]. Only Gilat and Gelman [69], Priede and Bagley [70], and Galil [53] made use of photographic equipment to observe the deep fauna in the Levantine basin. The biodiversity of fauna associated with hot spot ecosystems, such as seamounts, cold seeps, and deep corals, has been investigated only in the last two decades [71]–[75] (see Text S1 for additional references).

Studies of deep-sea benthic Foraminifera in the Mediterranean started in the late 1950s in both the western and eastern basins and extended in the 1970s, 80s, and 90s [76]–[79] (see Text S1 for additional references) down to 4,523 m depth. The following are among the more important studies in the deep Western Mediterranean. Parisi [80] worked on samples from bathyal depths (1,003–3,593 m) in the Tyrrhenian Sea and Straits of Sicily. Bizon and Bizon [81] reported on the geographic and bathymetric distribution of species down to 2,000 m off Marseille, Corsica, and in the Ligurian Sea. Schmiedl et al. [82], Heinz et al. [83], and Fontanier et al. [84] analyzed samples from the Gulf of Lions slope (343–1,987 m) and one site located at 920 m in the Lacaze-Duthier Canyon. Three studies have analyzed samples from the Eastern and Western Mediterranean; Cita and Zocchi [85] in the Alboran, Balearic, Tyrrhenian, Ionian, and Levantine basins (166–4,625 m); De Rijk et al. [86], [87] along bathymetric transects (20–4,000 m) from the same basins and the Tyrrhenian Basin and Straits of Sicily; and Pancotti (unpublished) from the Balearic Basin, Tyrrhenian Sea, Ionian Sea, and areas around Crete and Rhodes. The large number (hundreds) of samples studied, and the variation in their surface area, make it difficult to estimate the total area sampled.

The study of the diversity of benthic prokaryotic assemblages (Bacteria and Archaea) in deep-sea sediments of the Mediterranean Sea began only after 2000 [88], [89], when the development of molecular genetic tools [90] overcame the inability to culture the large majority of deep-sea prokaryotes on conventional culture media [91]–[93]. These tools have freed researchers from culturing biases (less than 1% of environmental microbes can be cultivated) and allowed characterization of community structure (e.g., 16S and 18S ribosomal RNA genes for prokaryotes and microeukaryotes, respectively) [90], [94]. Since then the number of sites explored and the number of samples analyzed have increased enormously, although most of the data are still being processed.

**Figure 1:**
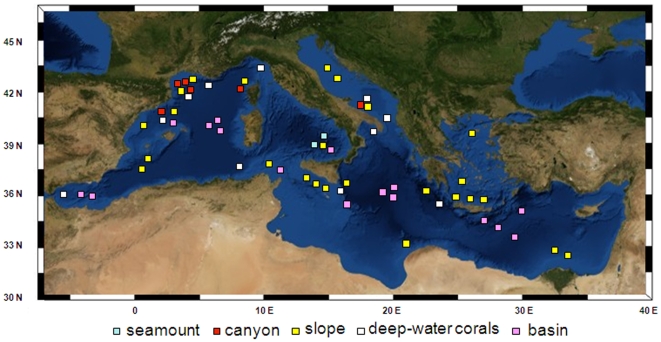
Investigated areas in the Mediterranean basin. Areas include slopes, seamounts, canyons, deep-water corals, and basin.

In this paper, we summarize the currently available information on deep Mediterranean biodiversity by examining and comparing the different components of the deep-sea biota, from Prokaryotes to Unicellular Eukaryotes, Meiofauna, Macrofauna, and Megafauna (including benthopelagic components). We performed an in-depth analysis of the main types of deep-sea ecosystems, including (a) open slopes, (b) deep canyons, (c) deep basins, (d) deep-water coral ecosystems, (e) hydrothermal vents, (f) cold seeps, and (g) deep anoxic basins. Figure 1 shows the areas where deep-sea samples and data have been collected for use in this paper.

## Results

### Prokaryotic diversity (Bacteria and Archaea)

**Figure 2:**
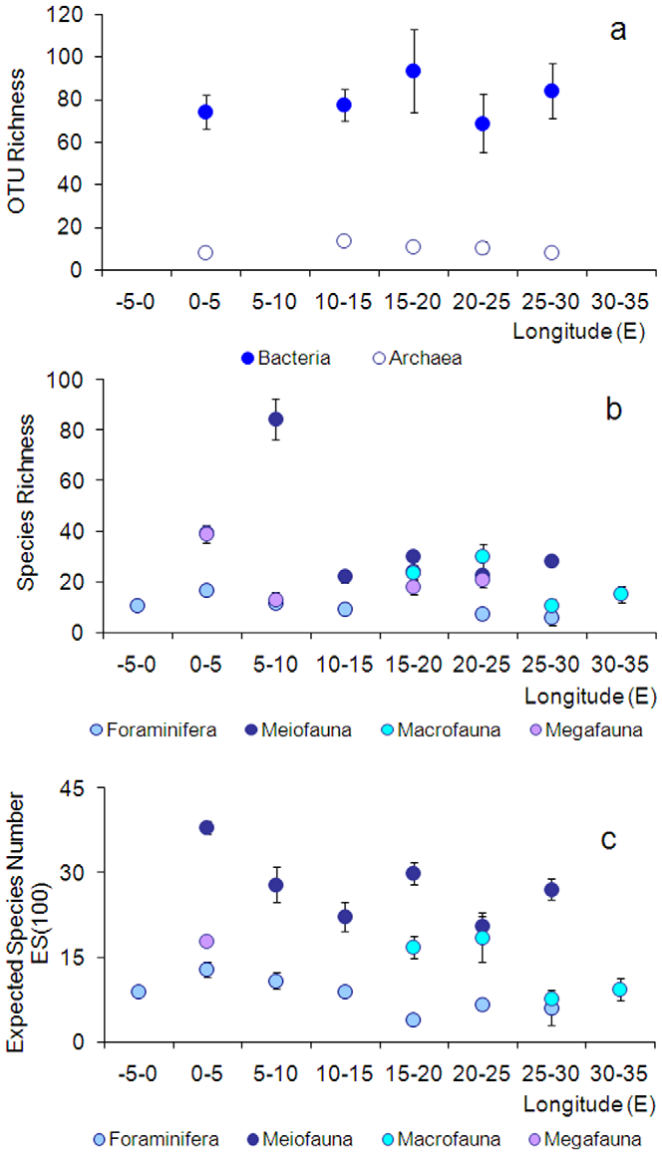
Longitudinal patterns of diversity in the deep Mediterranean Sea. Diversity is estimated as (a) bacterial and archaeal OTU richness (data obtained using ARISA and 16S rDNA T-RFLP fingerprinting technique, respectively, are unpublished); (b) Species Richness and (c) Expected Species Number estimated for 100 individuals (ES(100)) for Foraminifera, Meiofauna (as Nematoda), Macrofauna and Megafauna. Megafaunal data for ES(100) are from [26]. Reported are average values and Standard Error bars.

**Figure 3:**
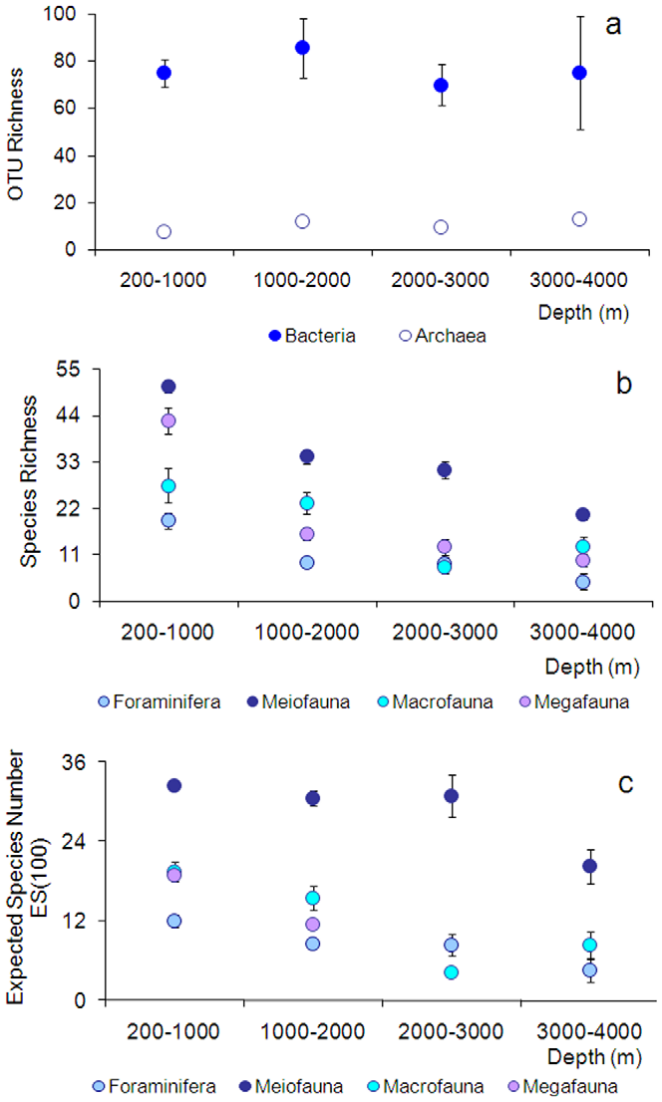
Bathymetric patterns of diversity in the deep Mediterranean Sea. Diversity is reported as (a) bacterial and archaeal OTU richness (data obtained using ARISA and 16S rDNA T-RFLP fingerprinting technique, respectively, are unpublished); (b) Species Richness and (c) Expected Species Number estimated for 100 individuals (ES(100)) for Foraminifera, Meiofauna (as Nematoda), Macrofauna and Megafauna. Megafaunal data for ES(100) are from [26]. Reported are average values and Standard Error bars. For the entire dataset of each component, the equations of the regressions are (1) *Y* = −0.0005 *X *+77.0 for the Bacteria (*n* = 54, *R^2^* = 0.0001, *p* not significant), (2) *Y* = 0.0015 *X *+7.4 for Archaea (*n* = 17, *R^2^* = 0.1692, *p* not significant), (3) *Y* = −0.0042 *X *+19.2 for Foraminifera (*n* = 172, *R^2^* = 0.0602, *p*<0.05), (4) *Y* = −0.0099 *X *+53.9 for Meiofauna (*n* = 171, *R^2^* = 0.1317, *p*<0.01), (5) *Y* = −0.006 *X *+31.4 for Macrofauna (*n* = 29, *R^2^* = 0.5150, *p*<0.01), (6) *Y* = −0.0005 X +48.1 for Megafauna (*n* = 57, *R^2^* = 0.3379, *p*<0.01).

Little is known about the biodiversity of benthic prokaryotes in the deep sea. This is particularly true in the Mediterranean Sea, where only limited and sparse studies have been carried out in “spot” locations in the Eastern Mediterranean, Cretan Sea, and South Ionian, [95]; southern Cretan margin [96] and the Ionian [88] and Tyrrhenian [97] seas (Table S1 and Text S2). The amounts of sediment that have been analyzed for bacterial and archaeal diversity in the deep Mediterranean Sea are on the order of a few tens of grams, clearly indicating that studies are just beginning (Figures 2 and 3). Available information on bacterial OTUs (operational taxonomic units) richness in the Mediterranean Sea highlights a high level of diversity ranging from 13 to 1,306 OTUs per gram of surface sediment, depending on the method used (fingerprinting or cloning/sequencing) [88], [89], [96]. These estimates do not include the “rare” taxa, which can be detected only by the powerful 454 pyro-sequencing technology. This technique, which has not been applied yet in deep-sea sediments of the Mediterranean Sea, is likely to increase significantly the estimates of bacterial species richness. Mediterranean sediments are highly diverse, displaying a bacterial richness comparable with deep Antarctic sediments [98] as well as with other deep-sea sediments [91], [92]. A comparative analysis of bacterial diversity from different oceanic regions highlights the peculiarity of the Mediterranean: the turnover diversity between Mediterranean and Atlantic sediments is about 85%, and reaches 97% between the Mediterranean and the South Pacific.

Our knowledge of benthic Archaea in the deep Mediterranean Sea is almost nonexistent. Recently, Mediterranean-specific archaeal “ecotypes” were identified in bathypelagic waters [99], while fingerprinting analyses to determine benthic archaeal OTU richness reported a diversity roughly 10 times lower than that for Bacteria (range 3–35 OTUs per gram of sediment; [100]). As in the case of bacterial assemblages, the composition of Mediterranean archaeal assemblages is significantly different from that of deep Atlantic sediments [100]. Interestingly, significant longitudinal differences could be observed between the Western, Central, and Eastern Mediterranean, with a turnover diversity reaching 99%, indicating high regional variability [95]. On the other hand, no bathymetric patterns of prokaryotic diversity have been observed in the Mediterranean sediments for either Bacteria or Archaea. The construction of 16S rDNA clone libraries [91], [92], [101] has revealed that Alpha-, Beta-, Gamma-, and Delta-Proteobacteria, Acidobacteria, Bacteroidetes, and Planctomycetes are widely distributed in most marine environments, while Alpha-Proteobacteria, Gamma-Proteobacteria, and Bacteroidetes appear to be common in deep-sea sediments [91], [92], [101], [102]. A phylogenetic analysis conducted on 207 bacterial 16S rDNA sequences from a large clone library in the South Ionian Sea at a depth of 2,790 m demonstrated that Acidobacteria was the dominant phylogenetic group, followed by Gamma-Proteobacteria, Planctomycetes, Delta-Proteobacteria, and Bacteroidetes [89], [95]. A few clones grouped with the Alpha-Proteobacteria, Beta-Proteobacteria, Actinobacteria, Verrucomicrobia, Chloroflexi, Nitrospirae, and Bacteroidetes. Recently, a total of 454 sequenced clones from the deep southern Cretan margin revealed the dominance of the phyla Acidobacteria, Planctomycetes, Actinobacteria, Gamma-, Alpha-, and Delta- Proteobacteria, and only few sequences were affiliated with the phyla Chloroflexi, Bacteroidetes, Firmicutes, Gemmatimonadetes, Verrucomicrobia, Nitrospirae, Beta-Proteobacteria, Lentisphaerae, and Dictyoglomi [96]. However, in the Eastern Mediterranean Sea, the phylum Acidobacteria dominated the microbial communities in the deep-sea sediments, followed by members of the Gamma- and Delta-Proteobacteria [95], [96]. Generally the presence of Acidobacteria phylum members has been associated with metal-contaminated, acidic sediments, or extreme conditions [103] and their presence in the deep Mediterranean and in pristine sediments remains questionable. In addition to the dominance of Acidobacteria, the phylotypes that have been identified from the Mediterranean sediment clone libraries were only distantly related to sequences included in the public databases (i.e., GenBank, [96]) whereas a large fraction of the retrieved sequences (12%) did not fall into any taxonomic division previously identified. These findings are consistent with data available from Mediterranean deep waters [104]. The still-limited available evidence indicates that deep Mediterranean sediments harbor an incredibly high and unique prokaryotic diversity, which is different from that described in other deep benthic environments. Mediterranean sediments can be considered as “bacterial hot spots.” The preservation of this biodiversity is enormously important for the ecological functioning of the entire Mediterranean basin, as well as, from a bioprospecting point of view, for potential future exploitation and sustainable use of deep Mediterranean resources.

### Foraminiferal diversity

Foraminiferal species richness and other diversity measures, as well as abundance, are reported to be lower in the Eastern than in the Central and Western Mediterranean, the lowest values being found in the deep Levantine Basin [85], [86] (Figure 2, Table S2 and Text S2). Rarefaction curves (Pancotti unpublished) generally show decreasing species richness from west to east, with highest values in the western part of the Balearic Basin (2,650–2,688 m) and lowest values in the Rhodes Basin (3,020 m) and in the south of Crete (2,090 m). Only three specimens representing a single species (a saccamminid) were recorded in the Ionian Basin (3,903 m). This east-to-west decline in species richness is probably related to the corresponding decrease in organic matter flux settling the seafloor [87]. In the Eastern Mediterranean, Cita and Zocchi [85] report a decrease in species richness from 11–64 at 1,000–1,800 m to 4–8 at 1,800–2,500 m and less than 8 at 2,500–4,000 m. This compares with 65–92 (1,311–1,867 m) and 19–71 (2,318–2,703 m) in the Western Mediterranean (Balearic Basin). Based on box core samples collected along bathymetric transects spanning the length of the Mediterranean, De Rijk and coworkers [86] reported a broad peak in species richness between 200 m and 1,000 m, below which richness decreased to 4,000 m, the maximum depth sampled. When the bathymetric distributions of individual species are considered (Figure 3), the upper and lower depth limits are usually found to be shallower in the more oligotrophic eastern basins than in the more eutrophic western basins [87]. Despite the differences in size fractions analyzed, when taken together, these data reveal a clear trend of decreasing species richness with depth, particularly in the South Adriatic Sea. Similar datasets for dead assemblages are available from studies in the Tyrrenian Sea and Sicily Channel (1,000–3,600 m, >63 µm fraction) [80] and in the Adriatic Sea (207–1,198 m, >150 µm) [78].

### Meiofaunal diversity

Nematodes are the dominant meiofaunal taxon (on average more than 80% of entire Meiofauna) and their Species Richness ranges from 3 to 159 species (Central and Western Mediterranean Sea; Table S3 and Text S2). The turnover diversity displayed high values of dissimilarity when nematode assemblages were compared from different depths (maximal values of 84% between the bathymetric ranges 200–1,000 m and 3,000–4,000 m) and longitudes (greater than 77% comparing Western, Central, and Eastern Mediterranean). This high variability in species composition is confirmed by the significant difference between nematode assemblages from different depths and longitudes (significance level less than 0.001). Nematode biodiversity displays a clear longitudinal gradient along open slopes, with values decreasing from west to east (Figure 2). At all longitudes, nematode Species Richness displays a high variability. It has been suggested that the longitudinal gradient could result from a decrease in productivity, and hence in food availability, in a west-to-east direction [18], [62]. These findings suggest that the spatial variability of food quality along the deep Mediterranean Sea influences the large-scale spatial patterns of biodiversity. This is consistent with a comparison of nematode diversity in the north and south Aegean Sea, where the contrasting surface primary production supports the hypothesis of a link between diversity and productivity [68]. These results suggest that organic inputs from the euphotic zone can have an important influence on nematode diversity. However, further analyses conducted at about 3,000 m depth revealed that nematode diversity was not associated with changing food availability or with organic input to the seafloor [61]. Diversity indexes may be strongly influenced by the local ecology of an area [7], [105], [106], and west–east differences in the deep-sea biodiversity could be also related to a different evolutionary history, related to the Messinian crisis. Unfortunately, there is not sufficient information available to clarify whether the observed nematode diversity patterns are also reflected by other taxa. Analysis of the bathymetric patterns of nematode diversity reveals the lack of unimodal patterns and no evidence for a decline with increasing water depth in the western basin; instead, Species Richness displays a high variability at all depths (Figure 3). Conversely, in the Eastern Mediterranean, nematode diversity increased from the continental shelf down to the bathyal zone (deeper than 1,000 m), where the highest diversity was found, and then decreased again down to depths greater than 2,000 m. This hump-shape pattern needs to be confirmed with the analysis of a larger dataset.

### Macrofaunal and megafaunal diversity

Despite the thorough review of Fredj and Laubier [107] regarding qualitative aspects of the benthic Macrofauna composition of the deep Mediterranean Sea, quantitative data from this basin are scarce (Figures 2 and 3, Table S4 and Text S2). Several investigations have described low-abundance and low-diversity conditions of marine invertebrates in the Eastern Mediterranean [35], [38], [43], [107]–[109]. The Gibraltar sill is, potentially, a physical barrier for the colonization of Mediterranean habitats by larvae and deep-sea benthic organisms from the richer Atlantic fauna, which could explain the low diversity observed for deep Mediterranean Macrofauna. Van Harten [110] hypothesized that several species of deep-water ostracods that are still common in the Western Mediterranean became extinct in the Eastern Mediterranean basin at the onset of early Holocene S1 sapropel deposition, which still make the bathyal bottoms unfavorable to faunal colonization (see Text S1 for more references). These results, however, were not confirmed by subsequent studies aimed at investigating the distribution of biodiversity across the Atlantic-Mediterranean region. Macpherson [111] and Galil [53] suggest that within the Atlantic-Mediterranean region, the fauna (including invertebrates and fishes) of the Mediterranean Sea is more diverse than that of the Atlantic and displays considerable endemism. In addition, except for strictly deep-dwelling species (e.g., the deep-water decapod crustacean family Polychelida), the Gibraltar sill is not an impenetrable barrier for some deeper-water macrobenthic species [112]. It has been hypothesized also that as a result of high deep-sea temperatures (about 10°C higher than in the Atlantic Ocean at the same depth), much of the present-day Mediterranean deep-sea fauna consists of reproductively sterile pseudopopulations that are constantly replenished through larval inflow [113]. However, populations of the most common benthic mollusk species at depths greater than 1,000 m in the Levantine Sea comprise both adult and juvenile specimens. Gravid benthic decapod crustaceans and fish have been collected repeatedly from the deep Levantine Sea [50], [52], [114] and Western and Central Mediterranean [115]–[127].

In the Catalan Sea (northwestern Mediterranean), 48 species of fishes have been collected between 400 m and 1,500 m, and among the most abundant are *Alepocephalus rostratus* and *Mora moro* [26] and Fernandez de Arcaya (unpublished data). Though much reduced in diversity and richness compared with the deep-sea fauna of the western and central basins of the Mediterranean, the Levantine bathybenthos appears to be composed of autochthonous, self-sustaining populations of opportunistic, eurybathic species that have settled there since the last sapropelic event. Working in the Cretan Sea, Tselepides and coworkers [20] reported mean benthic biomass, abundance, and diversity to decrease drastically with depth, and the occurrence of major faunal transitions at 200 m, 500 m, and 1,000 m depth. Although the deep Mediterranean is generally considered to be a “biological desert,” a moderate number of megabenthic species have been reported [26], [108], [123], [128], [129] even from the most oligotrophic regions of the Mediterranean, such as the Levantine Sea [53], [130] at depths between 400 m and 4,264 m. In the eastern basin, 20 species of decapod crustaceans have been encountered, including the endemic geryonid crab (*Chaceon mediterraneus*), which was photographed southwest of Cyprus at 2,900 m. One species, *Levantocaris hornungae*, was described as new to science [50], [131]. *Polycheles typhlops*, *Acanthephyra eximia*, *Aristeus antennatus*, and *Geryon longipes* were the most common species, comprising nearly 48%, 25%, 14%, and 7% of the specimens, respectively.

The same species are also dominant in the Cretan Sea and the Rhodos and Ierapetra basins. Among amphipod crustaceans, off Cyprus and Israel a total of 22 species (from 673 specimens collected) were encountered, and four of these were endemic to the Mediterranean. Two of these, *Ileraustroe ilergetes* and *Pseudotiron bouvieri*, represented 40% and 15% of the amphipod specimens, respectively. *Rhachotropis rostrata* and *Stegophaloides christianiensis* were the next most common, representing nearly 11% of the specimens. From the baited trap deployments in the Cretan Sea and the Rhodos and Ierapetra basins, *Scopelocheirus hopei*, *Scopelocheirus polymedus*, *Orchmenella nana*, *Orchomene grimaldi*, and *Epimeria cf. cornigera* were the most abundant amphipod species. Twelve species of cumaceans from a total of 575 specimens were collected: *Procampylaspis bonnieri* was the most frequently collected, representing 33% of the specimens, followed by *Campylaspis glabra* (13%) and *Makrokylindrus longipes*, *Platysympus typicus*, and *Procampylaspis armata* (each with nearly 11%). A total of 44 species of benthic mollusks were identified at depths greater than 1,000 m, the most common being *Yoldia micrometrica*, *Kelliella abyssicola*, *Cardyomia costellata*, *Entalina tetragona*, *Benthomangelia macra*, *Benthonella tenella*, and *Bathyarca pectunculoides*. Studies in the western basin have shown that non-crustacean invertebrates account for approximately 10% to 20% of total biomass and abundance of the benthic megafauna [26], [108]. Of these, mollusks and echinoderms are the groups with the highest species richness [26], [127]. The proportion of echinoderms is highly reduced compared with Atlantic fauna, the main species being the holothurian *Molpadia musculus*, the echinoid *Brissopsis lyrifera*, and the asteroid *Ceramaster grenadensis* [26], [129]. A total of 31 deep-sea fish species were collected off Cyprus and Israel, including *Bathypterois dubius* and *Nezumia sclerorhynchus* (38% and 27% of the total fish abundance, respectively). *Cataetyx laticeps*, *Chauliodus sloani*, and the ubiquitous *Bathypterois dubius* were photographed at 2,900 m depth. In baited-camera deployments in the Cretan Sea and the Rhodos and Ierapetra basins, *Chalinura mediterranea* (now *Coriphaenoides mediterraneus*) and *Lepidion lepidion* were the most abundant species. At 1,490 m depth, the sharks *Centrophorus granulosus* and *Etmopterus spinax* were the most abundant, occurring in 83% of the recordings. In the Cretan Sea and Rhodos Basin and at depths less than 2,300 m, the most abundant species were *Hexanchus griseus*, *Galeus melastomus*, *Centrophorus* spp., *Centroscymnus coelolepis*, and *Etmopterus spinax*.

In the deep Mediterranean Sea, information on diversity patterns and community structure of benthic megafauna is still scarce. Such studies in the Western and Central Mediterranean have focused on the two most abundant groups below 600 m depth: fishes [44], [116], [132] and decapod crustaceans [44], [124], [125], [128], [133]–[136]. There is an increase in the relative abundance of crustaceans relative to fish at depths below 1,500 m [128]. This change in the relative abundance of fish and decapod crustaceans has been explained by the low food availability at greater depths and the higher adaptation of crustaceans to low energy levels [48], [128]. The diversity patterns of the much less abundant noncrustacean benthic megafauna are virtually unstudied, with the exception of a few descriptive studies [107], [137], [138] and scarce quantitative data [26], [108], [129]. Fishes and crustaceans are mainly responsible for a megafaunal peak between 1,100 m and 1,300 m [13], [46], [116], [128], [132], [139], [140] that is related to high suprabenthos abundance between 800 m and 1,200 m on the slope [115], [141]. These high biomass levels have been attributed, in the Western Mediterranean, to the fishes *Alepocephalus rostratus*, *Trachyrinchus scabrus*, *Mora moro*, and *Lepidion lepidion*, and the crab *Geryon longipes* [48], [49]. Depth-related patterns of fish biomass and biodiversity have been reported by several authors, but with different zonations [116], [132], [142], [143]. Larger species are found between 600 m and 1,200 m depth (“bigger-deeper”), followed by a rapid decrease with increasing depth [49], [139], [141], [143], [144].

Megafaunal species richness decreases with depth between 600 m and 4,000 m both in the western and eastern Mediterranean basin [47], [48], [108], [123]. Biodiversity (H′) also decreases from 800 m and drops significantly below 1,500 m depth, while evenness increases [108], [116], [129]. Recent studies extend depth ranges in the Levantine Sea deeper than in the Western Mediterranean for 14 serpulid species, one-third of the depth extensions were deeper than 400 m (see Text S1 for more references). Twenty-three fish species were collected or photographed in the Levant Sea at depths greater than in the Western Mediterranean, some nearly doubling the depth record of the species [51], [52], [144]. Several mollusks—*Pteroctopus tetracirrhus, Crenilabium exile, Yoldiella philippiana, Bathyarca philippiana, Thyasira granulosa, Allogramma formosa, and Cuspidaria rostrata*—have been collected from greater depths in the Levantine Sea than elsewhere in the Mediterranean [145], [146]. Extension of the depth records was also reported for five of the bathyal amphipods collected off the Israeli coast, and for *Bathymedon monoculodiformis*, by as much as 1,100 m [147]. Species richness decreases from west to east along a longitudinal gradient in the Mediterranean [108], apparently reflecting the increased oligotrophy in the Levantine Basin [148], [149]. The Levantine Sea bathyfaunal scarcity may cause different parceling of the populations that is reflected in bathymetric distributions that differ from those of the Western Mediterranean deep-water assemblages.

### Deep-sea biodiversity hot spots in the Mediterranean Sea

**Figure 4:**
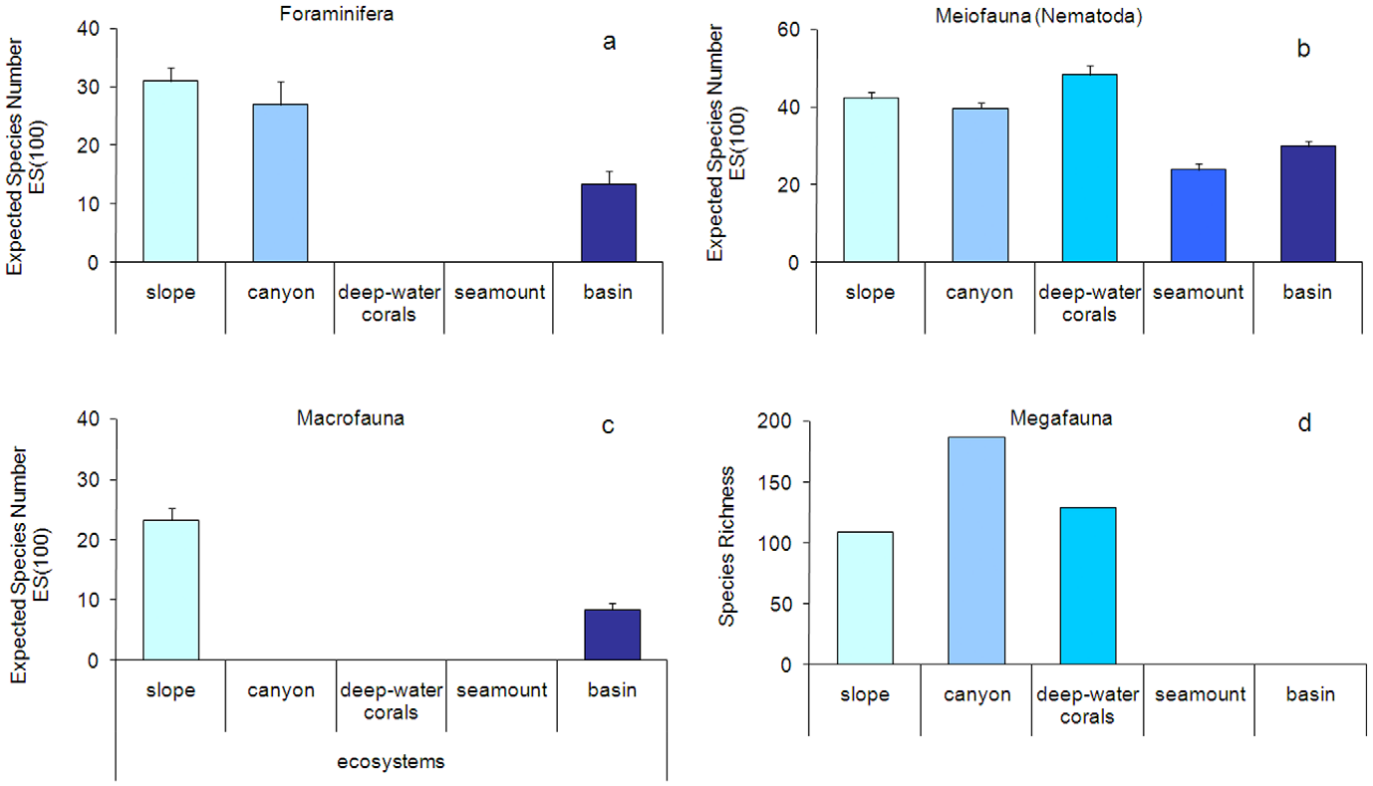
Biodiversity in slope, canyon, deep-water corals, seamount, and basin ecosystem of the deep Mediterranean Sea. Reported are (a) Foraminifera (data on live specimens), (b) Meiofauna (as Nematoda), (c) Macrofauna diversity as expected number of species for 100 specimens (ES(100)), and (d) Megafauna diversity as Species Richness.

The Mediterranean basin contains, over relatively limited spatial scales, a number of habitats that can represent potential “hot spots” of biodiversity. Knowledge of the biodiversity associated with these habitats and ecosystems is expected to enhance significantly our understanding of biodiversity and functioning of the deep seas. A tentative, possibly not exhaustive list of these systems includes (a) open slope systems, (b) submarine canyons, (c) deep basins, (d) seamounts, (e) deep-water coral systems, (f) cold seeps and carbonate mounds, (g) hydrothermal vents, and (h) permanent anoxic systems. A comparison of the benthic diversity among different ecosystems is reported in Figure 4. Here all of the species encountered in each habitat or ecosystem for each benthic component (from Foraminifera to Megafauna) are reported.

#### Open slope ecosystems

The continental slope represents the connection between the shelf and basin plain. The steepness of the slope allows the distinction between progressive, intermediate, and abrupt continental margins [16]. Margins facing the main rivers are generally progressive, with mainly fine-grained sediments. Landslides can shape the seafloor and mobilize huge volumes of sediments. All the studied margins show that the flux of particles increases with depth owing to the presence of lateral inputs, ranging from 50% in the Gulf of Lions to 80% in the Cretan Sea.

Slopes are ideal systems for investigating benthic patterns: the decrease of benthic abundance and biomass with increasing depth is one of the best-known patterns in marine ecology. An increasing number of studies suggest that we are not able to predict spatial distribution of deep-sea benthos using a limited set of variables. Danovaro et al. [63] investigated the biodiversity of meiofaunal (as richness of taxa) and nematode (as species richness) assemblages along the continental margins at large spatial scales and reported that open slopes display a species richness similar to, or higher than, that reported for bathyal and abyssal plain ecosystems. However, a unique, general driver capable of explaining the spatial patterns of biodiversity was not identified. This result is not surprising, considering the multiplicity of interactions among “local” ecological characteristics, environmental factors, and topographic and textural conditions in each specific slope environment. This complexity probably has considerable influence on the conditions, allowing settlement of a large number of species. The patterns of deep-sea biodiversity along the slope are different from those hypothesized so far, reflecting a mosaic of life more complex and varied than previously imagined. Further efforts should be devoted to increasing the spatial resolution of deep-sea investigations along open slopes. Understanding the mechanisms controlling deep-sea biodiversity within and across these attractive environments will open new perspectives for the conservation and sustainable management of open slope systems crucial to the functioning of the global ecosystem.

#### Canyon ecosystems

Submarine canyons are major topographic systems that enhance the heterogeneity of continental slopes [150]. These submarine valleys are mostly incised on the continental slope and form part of the drainage system of continental margins. Their cross sections tend to be V-shaped along the upper course and U-shaped in the lower course, thus reflecting the prevalence of erosion and accumulation processes, respectively. Submarine canyons are widespread on many continental margins, but their abundance and development vary greatly. Complex canyon networks (e.g., the Gulf of Lions) are sometimes adjacent to sections of the margin with only linear canyons (e.g., the Catalonia margin), or no canyons at all (e.g., the North Balearic margin). Submarine canyons probably have different origins, either submarine or subaerial, or both. Most canyons are relatively inactive, but others are characterized by an important sediment transport [151]. They are major pathways for the transportation and burial of organic carbon, and fast-track corridors for material transported from the land to the deep sea [152] and act as temporary buffers for sediment and carbon storage. Rapid, episodic flushing of canyons may at times transport large amounts of sediment to the deep basin [15]. Several submarine canyons cross the continental slope of the Western and Central Mediterranean. They represent hot spots of species diversity and endemism [153], [154] and are preferential areas for the recruitment of megafaunal species [46].

Canyons probably play an important role in structuring the populations and life cycles of planktonic fauna [154], as well as benthic megafauna fishery resources that are associated with them. For example, canyons are important habitats for fished species, such as hake (*Merluccius merluccius*) and for the rose shrimp *Aristeus antennatus* [16], [48], [135], [144], [155], [156]. Faunal abundance and biomass are usually higher inside the canyons than at similar depths in the surrounding habitat, but individual size is significantly smaller than on the adjacent open slope. Although information about the biology of submarine canyon fauna is still scarce, morphologic and oceanographic features of the canyons are understood to be the main factors influencing faunal characteristics [157]. For example, (a) suspension feeders may benefit from accelerated currents [158] and exposure of hard substrate in an otherwise sediment system; (b) demersal planktivores may exploit dense layers of krill and zooplankton that become concentrated in canyons during downward vertical migrations [159]; (c) accumulation of food for detritivores may be enhanced by high sedimentation rates and accumulation of macrophytic debris [157], [160], [161]. Because of their characteristics, the biodiversity of faunal assemblages can be markedly different from that on the adjacent open slopes—the so-called canyon effect [26]. Their biomass and abundance can be 2- to 15-fold higher than that in the surrounding areas at similar depths [157].

Species composition within canyons is also different from that found on the surrounding slopes. Canyon assemblages generally display lower diversity for the meiofaunal components because of the high dominance of a few species and the lower evenness [162]. On the other hand, certain canyons may contain a higher diversity of megafauna than the slopes and can be considered as hot spots of diversity as they may display high rates of endemism [1], [154]. This may be particularly important in oligotrophic areas, which must have mechanisms for the efficient recycling of energy at different scales. Therefore, certain canyons are characterized as areas of high diversity and production, and as such they may play an important role in processes related to the transfer of matter and energy in the Mediterranean Sea [163]. The analysis of foraminiferal diversity from canyon areas did not reveal the presence of species confined to canyon areas [81]. However, also in the Gulf of Lions, foraminiferal standing stocks and diversity (as Shannon-Wiener index) are both higher at an axial site in the Lacaze-Duthiers Canyon than on the adjacent slope (water depths 920 m and 800 m, respectively [82]). A comparative analysis of nematodes at similar depths in four deep-sea canyons and on adjacent open slopes in the Western and Central Mediterranean Sea suggested that species richness changed significantly with increasing water depth only in about half of the investigated systems. Both increasing and decreasing patterns in species richness were observed. The multivariate, multiple regression analyses indicated that quantity and quality of organic matter explained an important portion of the variances of the diversity indices, but also temperature and physicochemical conditions played an important role in determining the observed patterns. In addition, the analysis of nematode biodiversity revealed the presence of significant differences in species composition at different depths in all of the investigated systems, indicating that, independent of significant differences in species richness and organic matter content, bathymetric differences were always associated with significant changes in species composition. Overall, the biodiversity of nematodes (expressed as both species richness and rarefied species number) was not significantly different when canyons and adjacent open slopes were compared. Only at 500 m depth in the Central Mediterranean Sea was nematode diversity significantly lower in canyons than on slopes, possibly reflecting peculiar hydrodynamic conditions that restrict the colonization of species. However, topographic features could also contribute to the observed differences; for example, at 500 m depth in the Central Mediterranean Sea (South Adriatic margin), the lower nematode species richness in canyons could be related to the presence of hard substrates [164].

In the Eastern Mediterranean, canyon and slope sediments displayed a similar biodiversity, but nematode assemblages in canyons were characterized by higher dominance of various genera such as *Daptonema*, *Paralongicyatholaimus*, and *Pomponema*. An upper canyon site (450 m) in the Mergenguera canyon and adjacent slope (Catalan margin off Barcelona) showed higher species richness and biodiversity of non-crustacean invertebrates than the middle (600 m) and lower (1,200 m) slope sites [129]. This difference was attributed to higher habitat heterogeneity and higher organic matter availability. Furthermore, the presence or higher abundance of sessile taxa such as corals and sponges on the lower slope (1,200 m) was explained by intensified hydrodynamics associated with the proximity of the canyon, as well as by the lack of fishing activity at 1,200 m, which allows the establishment and maintenance of sessile and fragile species [129]. Crustacean biomass was also higher at the canyon site, while fish abundance was higher on the slope sites [44], [45]. In the Blanes canyon and adjacent margin, variations in community structure have been observed between two areas in the canyon (canyon head and canyon wall) and one site on the adjacent margin at similar depths [26]. Here, the community on the open margin has a lower species richness, lower diversity, and lower evenness. The MDS (multidimensional scaling) analysis and ABC (abundance-biomass curves) plots also separated the open margin community from the two canyon sites. These results can be explained by higher fishing intensity on the open margin, which has been affecting the benthic communities for over five decades [26], [48], [49].

#### Deep basins

The deep-sea basin of the Mediterranean Sea has been defined as bathyal or abyssal, based on different assumptions. According to some geologists, the Mediterranean Sea has no abyssal plains, and hence all the deep Mediterranean basins form part of the continental margin. In the Western basin the 2,600/2,700 m isobaths have been used as the upper limit of the abyssal plain, which has a maximum depth of about 3,050 m. In contrast, the Tyrrhenian Plain has been defined as bathyal [165], despite the fact that the deepest part of the Tyrrhenian Basin exceeds 3,600 m depth [14]. Bathyal and abyssal plains cover a large portion of the deep Western Mediterranean Basin [166], these having a triangular shape and an overall area of about 240,000 km^2^. Sediments filling the Mediterranean abyssal plains are dominated by the deposition of turbidities, but instead of being flat and homogeneous, as previously described, they are characterized by the presence of seafloor features up to 35 m in height [166]. The abyssal basins of the Mediterranean are extremely unusual deep-sea systems. With water temperatures at 4,000 m in excess of 14°C (rather than 4°C or colder for the deep oceanic basins) the entire benthic environment is as hot as the water around a hydrothermal vent system, but lacks the vents' rich chemical energy supply.

The Mediterranean also differs from other deep-sea ecosystems in its species composition, notably the absence of the near-ubiquitous deep-water grenadier fish *Coryphaenoides armatus* and the amphipod *Eurythenes gryllus*. Instead, *Acanthephyra eximia* appears to have functionally replaced *E. gryllus*, the dominant deep-sea scavenging crustacean throughout most of the world's oceans [167]. *A. eximia* is likely to have entered the Mediterranean Sea within the last 5 million years following Pliocene flooding by waters through the Strait of Gibraltar [168]. The Eastern deep basins formed roughly 2 million years ago, but stagnation precluded colonization for a long time [50]. A certain degree of eurythermy may have allowed *A. eximia* to become a dominant member of the Mediterranean abyssal community in the absence of the stenothermal amphipod *E. gryllus*. Barriers to colonization of the Mediterranean include the differences in temperature, salinity, and food supply between the Atlantic and Mediterranean, as well as the existence of shallow sills in the Strait of Gibraltar and Strait of Sicily. Despite these inferences and the relative youth of the system, a deep-sea fauna has developed, although it is depauperate compared with that of the oceans [130]. Typical deep-water groups, such as echinoderms, glass sponges, and macroscopic Foraminifera (Xenophyophora), are scarce or absent in the deep basins of the Mediterranean. Other groups (i.e., fishes, decapod crustaceans, mysids, and gastropods) are much less abundant in the deep Mediterranean than in the northeastern Atlantic.

#### Seamounts

Biogeographically, seamounts are islands separated by great depths. Consequently, they may serve as isolated refuges for relict populations of species that have disappeared from other areas. A complete and detailed map of all Mediterranean seamounts is not available yet. Moreover, investigations of seamounts have mainly been geological, while biological studies have been relatively neglected. In the Western Mediterranean, the Tyrrhenian bathyal plain is characterized by a large number of seamounts. These volcanic bodies of tholeitic petrology are either associated with north–south oriented crustal faults (Magnaghi, Vavilov, and Marsili seamounts) or with crescent-shape bathymetric ridges (horsts) bounded by normal faults, including the Vercelli and Cassinis ridges [169]. The Eastern Mediterranean basin is characterized by a higher topographic heterogeneity than the western sector and a large number of seamounts. The Eratosthenes Seamount is an impressive geological structure in the Levantine Sea, the biology of which is practically unknown. The only available biological information is given by Galil and Zibrowius [71], who report on the collection (with trawl and grab sampling at a depth of 800 m) of a limited number of benthic samples. Their work yielded a relatively rich and diverse fauna consisting mainly of two species of scleractinian corals (*Caryophylla calveri* and *Desmophyllum cristagalli*) (now *D. dianthus*), two types of encrusting foraminiferans, two species of encrusting poriferans, abundant scyphozoan polyps, many individuals of the small actiniarian *Kadophellia bathyalis*, seven species of bivalves, one sipunculan, one asteroid and one fish. Studies have been conducted recently on soft sediments surrounding the Marsili and Palinuro seamounts [97], [170]. The analysis of bacterial community structure revealed that the assemblages in the sediments close to these seamounts and the adjacent sediments were different. This indicates that, besides the consistently observed differences in the microbial variables, there are also differences in bacterial community composition between sediments from seamounts and sediments from other areas [97]. In addition, the authors found a much lower evenness (i.e., equitability of distribution of the OTUs among species) in Archaea than in Bacteria, which suggests that a few archaeal OTUs were dominant in these deep-sea sediments, whereas a much more equitable distribution characterized deep-sea bacterial assemblages. Overall, these findings indicate that the highest numbers of archaeal OTUs were observed in sediments close to the seamounts, where the lowest evenness and the highest viral production were also observed. Pusceddu et al. [170] emphasized that the biochemical composition of non-seamount sediments was largely different from that at Palinuro Seamount but were rather similar to the composition at Marsili Seamount. Moreover, the sediments close to the seamounts tend to harbor a small number of meiofaunal taxa and low nematode species richness, when compared with non-seamount sediments. At the same time, there were families and species exclusively present in sediments close to the seamounts and absent in adjacent sediments, and vice versa. These findings suggest that the deep-sea nematode assemblages of the Tyrrhenian Sea are highly site specific (i.e., they can vary at a regional scale within the same basin), and confirm previous studies that have indicated that the deep Mediterranean Sea can be characterized by extremely high turnover diversity among sites within the same basin [61]. Current research also involves other seamounts, such as the Vercelli and the Dauno seamounts and seamounts in the Alboran Sea. Nevertheless, the biodiversity of Mediterranean seamounts remains largely unexplored, and much work is needed to discover the potential contribution of these systems to the deep-Mediterranean biodiversity.

#### Deep-water coral ecosystems

A deep-water coral reef is a local seafloor mound consisting of accumulations of coral debris, fine- and coarse-grained sediments, and live coral colonies that provide additional hard substrate extending into midwater [171]. Thus, these reefs form locally elevated hard substrates associated with strong bottom currents that enhance food supply and prevent the settling of silt [172], [173]. The colonial stone corals *Lophelia pertusa* and *Madrepora oculata*, which occur along the northwestern European continental margin and the deep shelves and in Scandinavian fjords, are present also in different sectors of the deep-Mediterranean Sea. Zibrowius [174] provides a list of the areas where *L. pertusa* and *M. oculata* have been found in the northeast Atlantic and the Mediterranean, but the distribution of these habitats along Mediterranean margins is still incompletely known. Our knowledge of Mediterranean deep-water coral reefs comes from scientific and fishing dredge and trawl hauls. The first record of living colonial corals in the northern Ionian Sea dates back to the *Pola* expedition of 1891 (see Text S1 for more references). Information on macro- and megafauna associated with deep-water stony corals in the “hard-bottom community of white corals” was first reported by Pérès and Picard [175]. Recently, new technologies such as the multibeam echo sounder, side scan sonar, remotely operated vehicles (ROVs), and submersibles have been used to investigate the deep-water corals in the Mediterranean.

At present, a total of 14 coral bank areas have been censused, but only a few of them have been examined by ROV dives. These include the areas from the Gibraltar sill to the Gulf of Lions canyons, from the Ligurian Sea to the Sicilian Channel, and from the Apulian margin to the trough off Tassos in the Aegean Sea [75] (see Text S1 for more references). The depth distribution of the corals ranges from 150 m (Strait of Gibraltar) to 1,100 m (Santa Maria di Leuca). In the Mediterranean, cold-water corals generally occur along the edge of the continental shelf, on offshore submarine banks and in canyons. These coral communities share a set of common characteristics, including hydrographic conditions and food supply within a complex local topographic setting. Mediterranean deep-water reefs are associated with temperatures ranging from 13.4°C to 13.9°C, salinities from 38.4 to 38.9, and dissolved oxygen from 3.75 to 4.54 ml L^−1^ [75]. The temperatures in the deep Mediterranean are close to the upper limit for many cold-water corals living at bathyal depths [173]. The occurrence here of the two deep-water colonial scleractinian species living in the Mediterranean, *M. oculata* and *L. pertusa*, appears to be a relict of a much more extensive distribution during the Pleistocene [74], [137]. Most of the deep-water scleractinian species living in this basin are solitary [174], and only *M. oculata* and *L. pertusa* (so-called white coral community) are distributed on bathyal hard grounds [35]. Some of the solitary species, such as *Desmophillum dianthus*, also contribute to the reef frameworks. Cold-water corals are passive suspension feeders, which depend on the supply of particulate organic matter and zooplankton for their subsistence and are therefore preferentially distributed on topographic irregularities, such as prominent steps on canyon slopes and seamounts where currents are strong and sedimentation rates are low [172] (see Text S1 for more references). Although no quantitative comparison can be made as a result of different sampling efforts and equipment used, species richness appears to be higher in the SML coral reef. Here, both *M. oculata* and L. *pertusa* are present, together with the black coral *Leiopathes glaberrima* and a large number of poriferan species, which contribute to increase the habitat heterogeneity of the system [72], [74], [75], [176]–[178] (see Text S1 for more references). Overall, 222 species (19 still unidentified) were encountered in the SML coral area at depths between 280 m and 1,121 m [179]. The most diverse taxa were Porifera (36 species), followed by Mollusca (35), Cnidaria (31), Annelida (24), Crustacea (23), and Bryozoa (19). A total of 40 benthopelagic fish species were also collected. Other taxa, such as brachiopods and echinoderms, included a lower number of species. The species *Aka infesta* and *Paromola cuvieri* were recorded for SML coral area by Schönberg and Beuck [176] and Freiwald et al. [75], respectively. The sponge assemblage in the SML shows a high affinity with the fauna from the Boreal region with a small number of Mediterranean endemic species. Six scleractinian species were found: *M. oculata*, *L. pertusa*, *Dendrophyllia cornigera*, *Desmophyllum dianthus*, *Stenocyathus vermiformis*, and *Caryophyllia calveri*.

The gorgonians *Bebryce mollis*, *Swiftia pallida*, and *Paramuricea macrospina* as well as the hydrozoans *Clytia linearis* and *Halecium labrosum* were also reported in this system [193]. Most of the species are boreal and cosmopolitan. Among the 35 species of mollusks encountered in the SML area, none was shared with the Lacaze-Duthiers area, suggesting the possible presence of specific assemblages at each deep-water coral site. The most common polychaete associated with both *Madrepora* and *Lophelia* colonies was *Eunice norvegica*, which, together with *Serpula vermicularis*, was also found in Lacaze-Duthiers canyon, Cassidaigne canyon, and Strait of Sicily. Another polychaete, *Vermiliopsis monodiscus*, could be endemic to the Mediterranean basin, while *Harmothoë vesiculosa* is the first record for the Mediterranean. Very few crustacean species were encountered (*Bathynectes maravigna*, *Ebalia nux*, *Munida intermedia*, *M. tenuimana*, *Rochinia rissoana*, *Alpheus platydactylus*, and *Pandalina profunda*). The bryozoans *Schizomavella fischeri* and *Schizoporella neptuni* grow preferentially on deep-water corals, and three species (*Puellina pedunculata*, *P. pseudoradiata pseudoradiata*, and *Setosellina capriensis*) are considered endemic to the Mediterranean. Megafauna (cephalopods, decapod crustaceans, and fish) of the SML coral area showed a larger size, biomass, and abundance inside than outside the coral area [179], [180]. The SML coral habitat seems to act as a spawning area for the rockfish *Helicolenus dactylopterus* and a nursery for the deep-water shark *Etmopterus spinax* and the teleosts *Merluccius merluccius*, *Micromesistius poutassou*, *Phycis blennoides*, and *H. dactylopterus*. A highly diversified fauna, characterized by the presence of living *M. oculata* together with *Corallium rubrum*, was also recorded in the Lacaze-Duthiers and Cassidaigne canyons [181] (see Text S1 for more references). The most abundant taxa, which varied according to the sampling method used and the attention given to the different groups, were cnidarians, bryozoans, mollusks, annelids, echinoderms, crustaceans, and fish. Epibiotic bryozoans growing on deep-water corals were found to be different from shallow-water assemblages and constituted a greater proportion of Boreo-Atlantic species [182]. In addition, complexity of the coral community in the canyons and the presence of many suspension and filter feeders, were related to the energetic trophic conditions characteristic of this type of habitat.

A total of 51 benthic species, among them poriferans, cnidarians, brachiopods, mollusks, polychaetes, crustaceans, and echinoderms, have been recorded in the Strait of Sicily, where the deep-water corals are located in three main areas [75], [183], [184]. Not all the fauna reported by Zibrowius and Taviani [183] was found alive. Recent observations by ROV off Malta revealed thick fossil coral frameworks with overgrowing coral assemblages mainly consisting of *M. oculata* and *L. pertusa* associated with *Corallium rubrum* and gorgonians [75]. The colony bases were generally inhabited by the symbiotic polychaete *Eunice norvegica*, and in some dives *Dendrophyllia cornigera* was detected. Observations from ROV dives in the Linosa Trough showed the fossil and modern coral communities thriving under overhangs and in large caves, and they were particularly common in volcanic bedrock sequences. In the Urania Bank, the colonies of *M. oculata* measured up to 70 cm high and 50 cm wide, while those of *L. pertusa* rarely exceed 10 cm in size [75]. More than 980 species have been reported from the Atlantic deep-water coral reefs [185] and 361 taxa were found in the Sula Reef [186]. Although the Mediterranean deep-water coral systems are considered less diverse than the Atlantic ones [35], [172], the data recently acquired demonstrate that this is not the case, especially if we consider that some of the taxa investigated in the Atlantic have not yet been investigated in Mediterranean deep-water corals habitats. Cephalopods, crustaceans, and fish can be attracted by the structural complexity of the deep-water coral reefs, which may act as essential habitats for feeding and spawning. Although none of the benthopelagic species so far recorded occurs exclusively in the coral habitat, many of them can be collected in greater abundance within coral habitats than in surrounding areas of seabed. In agreement with studies carried out in the Atlantic [187]–[191], significant differences were detected between the species abundance recorded within the SML coral area and that recorded in surrounding muddy bottoms [180]. The deep-water coral habitats can act as spawning areas for some species and nursery areas for others, as suggested by the higher catches of benthopelagic species (such as the shrimp *Aristeus antennatus* and *Aristaeomorpha foliacea*), as well as sharks, hakes, rockfish, greater fork beard, gurnards, and blackspot seabream by long-line in these areas [180], [192]. Studies on prokaryotic assemblages associated with the deep-sea coral *Lophelia pertusa* in the Central Mediterranean Sea revealed that they possess a specific microbial assemblage, which is different from that observed on dead corals and on surrounding sediment samples [193]. The majority of coral-associated OTUs were related to the Holophaga-Acidobacterium and Nitrospira divisions (80%), while more than 12% formed a separate deep-branching cluster within the Alpha-Proteobacteria with no known close relatives [193]. These authors reported that Archaea were not detected on living *L. pertusa* specimens, in contrast to previous findings on tropical coastal corals [194].

#### Hydrothermal vents

Most hydrothermal vents in the Mediterranean with described biological assemblages occur in shallow depths of less than 100 m [195]. Consequently, a profound difference between these and the described oceanic deep-sea vents is the occurrence of photosynthetic primary production. Also, the species that inhabit shallow-water Mediterranean hydrothermal vents are not endemic to these habitats but represent a subgroup of the most tolerant species in the ambient fauna. The only published evidence for deep-sea hydrothermalism in the Mediterranean consists of indicators of extinct activity observed on the peak of Marsili Seamount in the Tyrrhenian Basin at about 450–500 m depth [196].

#### Cold seeps and mud volcanoes

The first biological evidence for reduced environments was the presence of Lucinidae and Vesicomyidae shells cored on the top of the Napoli mud volcano, located at 1,900 m depth on the Mediterranean ridge in the subduction zone of the African plate [197]. This was followed by the description of a new Lucinidae bivalve species, *Lucinoma kazani*, associated with bacterial endosymbionts [198]. In the southeastern Mediterranean, communities of polychaetes and bivalves were also found associated with cold seeps and carbonates near Egypt and the Gaza Strip at depths of 500–800 m, but no living fauna was collected [199]. The first in situ observations of extensive living chemosynthetic communities in the Eastern Mediterranean Sea prompted cooperation between biologists, geochemists, and geologists. During submersible dives, communities comprising large fields of small bivalves (dead and alive), large siboglinid tube worms, isolated or forming dense aggregations, large sponges, and associated endemic fauna were observed in various cold seep habitats associated with carbonate crusts at 1,700–2,000 m depth. Two mud volcano fields were first explored, one along the Mediterranean Ridge, where most of them were partially (Napoli, Milano mud volcanoes) or totally (Urania, Maidstone mud volcanoes) affected by brines, and the other on the Anaximander mounds south of Turkey. The latter area includes the large Amsterdam mud volcano, which is affected by recent mudflows, and the smaller Kazan or Kula mud volcanoes [200], [201]. Gas hydrates have been sampled at the Amsterdam and Kazan mud volcanoes, and high methane levels have been recorded above the seafloor [202]. Several provinces of the Nile deep-sea fan have been explored recently. These include the very active brine seepage named the Menes Caldera in the eastern province between 2,500 m and 3,000 m [203], the pockmarks in the central area along mid- and lower slopes [204], and the mud volcanoes of the eastern province, as well as one in the central upper slope (North Alex area) at 500 m depth [205].

During these first exploratory dives, symbiont-bearing taxa that are similar to those observed on the Olimpi and Anaximander mud fields were sampled and identified. This similarity is not surprising, as most of these taxa were originally described from dredging in the Nile fan [206]. The updated table (Table S5 and Text S2) shows the diversity of the fauna in the various seep habitats explored since 1998. Up to five species of bivalves harboring bacterial symbionts colonized these methane- and sulfide-rich environments. A new species of Siboglinidae polychaete, the tubeworm colonizing cold seeps from the Mediterranean ridge to the Nile deep-sea fan, has just been described [207]. Moreover, the study of symbioses revealed associations with chemoautotrophic Bacteria, sulfur oxidizers in Vesicomyidae and Lucinidae bivalves and Siboglinidae tubeworms [200], [208], [209], and highlighted the exceptional diversity of Bacteria living in symbiosis with small Mytilidae [210]. The Mediterranean seeps appear to represent a rich habitat characterized by megafauna species richness (e.g., gastropods) or the exceptional size of some species such as sponges (*Rhizaxinella pyrifera*) and crabs (*Chaceon mediterraneus*), compared with their background counterparts. This contrasts with the low macro- and mega-faunal abundance and diversity of the deep Eastern Mediterranean. Seep communities in the Mediterranean that include endemic chemosynthetic species and associated fauna differ from the other known seep communities in the world at the species level but also by the absence of the large size bivalve genera *Calyptogena* or *Bathymodiolus* [211], [212]. The isolation of the Mediterranean seeps from the Atlantic Ocean after the Messinian crisis led to the development of unique communities, which are likely to differ in composition and structure from those in the Atlantic Ocean. Further expeditions involved quantitative sampling of habitats in different areas, from the Mediterranean Ridge to the eastern Nile deep-sea fan [213]. Finally, cold seeps recently discovered in the Marmara Sea [214] have also revealed chemosynthesis-based communities that showed a considerable similarity to the symbiont-bearing fauna of eastern Mediterranean cold seeps [213].

#### Deep hypersaline anoxic systems

Numerous deep hypersaline anoxic basins (DHABs) have been discovered in the Eastern Mediterranean Sea, the Red Sea, and the Gulf of Mexico. The six DHABs of the Eastern Mediterranean (L'Atalante, Urania, Bannock, Discovery, Tyro, and La Medee) are located on the Mediterranean Ridge. The Mediterranean DHABs lie at depths ranging from 3,200 m to 3,600 m and contain brine, the origin of which has been attributed to the dissolution of 5.9- to 5.3-million-year-old Messinian evaporites [215]. Brines enclosed in these basins are characterized by high abundances, which hamper the mixing with overlying oxic seawater and result in a sharp chemocline and anoxic conditions. The combination of nearly saturated salt concentration and corresponding high density and high hydrostatic pressure, absence of light, anoxia, and a sharp chemocline makes these basins some of the most extreme habitats on earth.

The brines of the L'Atalante, Bannock, and Urania basins have similar dominant ion compositions, but in the Urania the overall salinity is lower, whereas concentrations of sulfide and methane are considerably higher [216]. The Discovery basin is unique in that the brines have an extremely high concentration of Mg^2+^ and low concentration of Na^+^ [216] and represents the marine environment with the lowest reported water activity [217]. Studies of prokaryotic life in the Discovery, L'Atalante, Urania, and Bannock basins using epifluorescence microscopy, analyses of 16S ribosomal RNA (16S rRNA) gene sequences, and measurement of sulfate reduction, methanogenesis, and heterotrophic activity have revealed metabolically active bacterial and archaeal communities [217]–[222]. Van der Wielen and coworkers [216] investigated prokaryotic communities in all of the Mediterranean DHABs. They reported that Bacteria dominated the Discovery basin and were slightly more abundant in L'Atalante and Bannock basins, whereas Archaea dominated the Urania basin. In all four hypersaline basins, bacterial diversity was higher than archaeal diversity, and the Urania basin displayed the lowest overall diversity. Analyses of the 16S rRNA gene sequences showed that high percentages of clone sequences obtained from the four different deep hypersaline anoxic basins belonged to Gamma-, Delta-, and Epsilon-Proteobacteria, Sphingobacteria, candidate division KB1, and Halobacteria. Many of the dominant archaeal sequences belonged to the new subdivision MSBL1. Phylogenetic analyses based on 16S rRNA gene sequences revealed that microbial communities found in the brines are not found in normal seawater [216]. Such differences are probably related to the different geochemical conditions of the different basins together with their physical separation from each other and isolation from the oxygenated deep-water layers for possibly millions of years. This isolation may have resulted in the evolution of specific microbial communities in each DHAB. The analysis of prokaryotic diversity across the seawater-brine interface of the Bannock, L'Atalante, and Urania basins revealed that many prokaryotic taxa, including phylogenetically new groups, collectively formed a diverse, sharply stratified deep-sea ecosystem [218], [221], [222].

In both the Bannock and L'Atalante basins, Bacteria and Archaea were present in similar abundances in the oxic seawater above the hypersaline brine, whereas the seawater–brine interface was dominated by Bacteria and showed a bacterial diversity higher than in the overlying deep seawater. In the Bannock basin, five new candidate divisions (MSBL2, 3, 4, 5, and 6) were also identified in the seawater-brine interface through clone libraries. Microbial communities of the upper level of the halocline (meso-bathypelagic waters) displayed a large abundance of Crenarchaeota, whereas the bottom layers hosted different groups of Euryarchaeota. Members of the Haloarchaea were found only in a narrow window of the halocline at 130% salinity. In the Urania Basin, the seawater–brine interface and the brine were largely dominated by Bacteria, and Archaea contributed less than 0.2% of the prokaryotic 16S rRNA gene [222]. The overlying oxic seawater was dominated by Alpha- and Gamma-Proteobacteria and Fibrobacteres, whereas the anoxic layers were dominated by Delta- and Epsilon-Proteobacteria. A recent study carried out on the thermal mud fluids of Urania Basin, revealed the presence of a highly diverse prokaryotic community [220], mostly composed of unculturable prokaryotes. Archaeal diversity was much lower than bacterial diversity (more than 96% of the archaeal clones belonged to the MSBL-1 candidate order). About 60% of all bacterial and 40% of all archaeal phylotypes were encountered only in mud fluids and not in the upper layers of the brines. Here, dominant phylotypes are affiliated with the Epsilon-Proteobacteria subdivision and Delta-Proteobacteria. A novel monophyletic clade was also retrieved from deep-sea sediments and halocline of the Urania Basin.

Recently, the first metazoa living in the permanently anoxic conditions of the L'Atalante basin were discovered [223]. Danovaro et al [223] reported that the sediments of the L'Atalante basin were inhabited by three species of the animal phylum Loricifera (*Spinoloricus nov. sp*., *Rugiloricus nov. sp.* and *Pliciloricus nov. sp.*) new to science. Using different techniques, Danovaro et al [223] provided evidence that these organisms were metabolically active and showed specific adaptations to the extreme conditions of the deep basin, such as the lack of mitochondria, and a large number of hydrogenosome-like organelles, associated with endosymbiotic prokaryotes.

## Discussion

### Biodiversity patterns of different deep-sea benthic components and comparative analysis of the drivers

Little is known about longitudinal gradients across the deep-sea regions. Previous studies suggested that the west–east gradient of decreasing surface water productivity of the Mediterranean Sea is reflected in a corresponding gradient of decreasing food availability in deep-sea sediments [18], [62]. Such a gradient could be responsible for a significant decrease in the abundance and biomass of most benthic components, including Meiofauna, Macrofauna, and Megafauna. However, surprisingly our results indicate that there is no corresponding gradient for most components of benthic biodiversity (e.g., number of species and ES(100); Figure 2). Only the diversity of Foraminifera showed an apparent east-to-west increase in species richness [85]–[87]. However, data on Foraminifera have mainly originated from geological studies that employ varied methodologies (e.g., different sieve fractions, depth intervals, wet vs. dry sorting, dead vs. live assemblages), which often hamper a thorough statistical synthesis of the data. Conversely for other biodiversity components, such as benthic prokaryotes, higher biodiversity values were occasionally observed in the central-eastern sector of the deep Mediterranean. Finally, some deep-sea benthic components showed highly variable diversity values at all longitudes, without any significant patterns across the regions investigated (Figure 2). The longitudinal trends are therefore apparently weak and inconsistent across different components of the deep-sea biota. These results suggest that the effects of food supply (energy availability) may be important for certain components but can be compensated or masked by other factors that influence deep-sea diversity.

Bathymetric gradients of species diversity have been more widely documented than longitudinal gradients [4], [106], [224] (see Text S1 for more references). A central paradigm of marine diversity is that species richness increases with increasing water depth to a maximum at mid-slopes (around 2,000 m) and thereafter decreases [224], [225]. The enhanced levels of biodiversity along slopes are possibly a source for biodiversity for deeper basins and shelves, through radiation and dispersal processes closely coupled with benthic topography and the hydrodynamic, physical, and biogeochemical characteristics of the deep sea. The recent “source-sink hypothesis” [226] suggests, indeed, that abyssal biodiversity is a subset of bathyal biodiversity (in particular the biodiversity of the slopes at depths typically between 1,000 m and 2,500 m). However, this hypothesis has so far only been tested for gastropods and bivalves [12], and many studies have provided evidence of reproductively active abyssal species. Results reported here (Figure 3) indicate that none of the benthic faunal components displayed the unimodal pattern of biodiversity with peaks at intermediate water depths (1,500–2,500 m) [226]. Therefore, the hump-shaped curve does not reflect the patterns of deep-sea biodiversity in the Mediterranean Sea.

The comparison between bathymetric patterns of biodiversity expressed as species richness and expected species number provides evidence of a generally negative slope for species richness. Such a pattern is probably related to the exponential decrease of abundance observed for all animal components. However, different benthic components display different spatial patterns with increasing depth. For instance, the number of bacterial and archaeal OTUs did not change significantly with increasing water depth, indicating that the biodiversity of benthic prokaryotes encountered at the greatest depths was similar to the values reported at 200 m depth. This result is consistent with the patterns of organismal abundance described by Rex et al. [227], who reported that the abundance of three animal groups (Meiofauna, Macrofauna, and Megafauna) decreased significantly with depth, while bacterial abundance remained constant. As reported for patterns in animal abundance, the biodiversity of all other benthic components decreased significantly with increasing water depth. However, the slopes of the abundance values differed significantly; the biggest difference was observed comparing the Mega- and Macrofauna that decrease with depth more rapidly than the Meiofauna [227]. We found the opposite for biodiversity. In fact, while the slopes of the abundances with increasing depth is greater for Prokaryotes than Meiofauna than Macrofauna than Megafauna, we found the slopes of biodiversity greater for Megafauna than Macrofauna than Meiofauna (as Nematoda; analysis of covariance, Johnson-Neyman tests). Finally, the slope of Foraminifera displayed intermediate values between Meiofauna and Macrofauna. These results suggest that even though abundance of Mega- and Macrofauna decreases exponentially with depth, a large number of species can be found at great depths, while the abundance of nematodes decreases with depth to a lesser extent, but this is associated with a stronger reduction in species richness. This finding could indicate that the patterns of biodiversity could be dependent on the size of the organisms and probably the greater ability of larger organisms to move and disperse across different bathymetric ranges, which can be crucial for shaping biodiversity patterns.

If spatial patterns of biodiversity in the deep sea are beginning to be clarified, our comprehension of the mechanisms driving these patterns is still poor. Various biological and environmental factors have been proposed to explain why species diversity changes with depth. Those more frequently invoked are (a) sediment grain size and substrate heterogeneity, (b) productivity, organic content or microbial features, (c) food resources, (d) oxygen availability, (e) current regimes, and (f) catastrophic disturbances [4], [225] (see Text S1 for more references). However, for each deep-sea biotic group (Prokaryotes, Foraminifera, Meiofauna, Macrofauna, and Megafauna), or for each phylum or lower taxonomic level within each benthic component, these factors can act in different combinations and can overwhelm other local or regional factors, thus causing unpredictable biotic responses [225]. Our analysis, providing the first detailed look at benthic biodiversity patterns along depth gradients, suggests that while the decrease in organic carbon input with depth can control benthic organismal abundance along depth gradients [227], the same could not hold true for the benthic biodiversity. For instance, bacterial and prokaryotic abundance remain high and rather constant throughout the depth range both at a global scale [227] and in the deep Mediterranean [99], and similar patterns are observed in bacterial and archaeal diversity. In the Central Mediterranean Sea, changes in quality and quantity of organic matter were associated with shifts in bacterial community structure, but not to different biodiversity values [88]. Buhring et al. [228] demonstrated that the Eastern Mediterranean is characterized by impoverished, “energy-thirsty” benthic microbial assemblages, which respond rapidly to inputs of fresh organic matter and are characterized by a well-developed benthic microbial loop [229] (see Text S1 for more references). The richness of bacterial assemblages inhabiting these energy-poor sediments is extremely high and comparable to estimates obtained from terrestrial ecosystems, indicating that deep-sea prokaryotic species of the eastern basin may have evolved under starvation stress to optimize the use of the available organic matter. As far as the archaeal component is concerned, temperature could be important in explaining the variance of deep benthic archaeal OTU richness, while water depth has apparently a negligible role. However, the information available is still too limited to fully understand which environmental factors influence the patterns of prokaryotic biodiversity in Mediterranean deep-sea sediments [96], [95]. Thus, we conclude that the drivers of prokaryotic diversity in the deep-sea sediments of the Mediterranean Sea have yet to be identified.

Among Foraminifera, the abundance of deep-infaunal species decreases from west to east, corresponding to the productivity gradient. Previous studies suggested that the diversity of Foraminifera is related to organic matter flux settling to the seafloor [225], [230], and that the same could apply to the deep Mediterranean. Deep infaunal species virtually disappear in the Eastern Mediterranean, where the sparse fauna consist almost entirely of shallow infaunal species living close to the sediment surface [87]. Deep infaunal species are believed to consume low-quality, degraded organic matter and Bacteria, whereas shallow infaunal species are believed to consume labile material [230]–[233]. Moreover, some deep infaunal species store nitrate that they respire to dinitrogen gas [234], [235]. These ecological contrasts suggest that faunal differences between the Western and Eastern Mediterranean may have consequences for ecosystem functioning.

Deep-sea nematode assemblages are characterized by relatively high biodiversity values at all depths. In accordance with previous studies [236], the number of taxa decreases with increasing depth along the open slopes in all investigated areas. However, the patterns in the deep Mediterranean are not always evident when comparing the western and central-eastern basins. In addition, biodiversity patterns can display either decreasing or increasing trends with increasing depth, depending on the system investigated (e.g., slopes or canyons) [64], [162], [237]. These results suggest that biodiversity patterns are also dependent on different topographic and ecological features. This underlines the importance of better understanding specific topographic features and suggests new approaches for the investigation of deep-sea biodiversity, which needs to be tightly linked to the geosphere characteristics.

The quality and quantity of the food supplied to the seafloor are assumed to be the most important factor affecting the composition and abundance of deep-sea Macro- and Megafauna [238]. The deep Mediterranean Megafauna is significantly impoverished when compared with similar Atlantic and Pacific ecosystems [239]. The overall biomass of Megafauna (fish and crustaceans) in the western Mediterranean varies from about 150 kg km^−2^ below 800 m depth to a peak of about 1,200 kg km^−2^ at 1,200–1,300 m depth, decreasing again to less than 200 kg km^−2^ below 2,000 m depth [26], [108], [128]. In the Porcupine Seabight (northeastern Atlantic), Lampitt et al. [240] report Megafauna biomass of 5,000 to 10,000 kg km^−2^, which is an order of magnitude more than that observed in the Mediterranean. Despite Megafauna composition displaying differences between the western and eastern basins, similar bathymetric patterns of species richness have been observed [116], [123], [128]. Below 1,000 m depth, the species of the Macrouridae and Moridae families were dominant in all areas investigated. The main differences recorded in the Megafauna throughout the Mediterranean concern the occurrence and the abundance of some species, such as the crustacean *Stereomastis sculpta* and the fish *Alepocephalus rostratus*, in the Western Mediterranean and the total lack in the eastern basin [123], [128]. The shark *Centroscymnus coelolepis* seems to be exclusively distributed in the Western Mediterranean [241], [242], while *Centrophorus granulosus* is present also in the eastern basin. Its occurrence in the eastern basin was only recorded using lander platforms equipped with baited cameras [70], which can provide only images from which the taxonomic identification is uncertain. The absence of *Centroscymnus coelolepis* in the Eastern Mediterranean remains an open question [50] but could be due to the distance from the point of faunal entry at the Gibraltar Strait or to the difficulty that a truly deep species faces in passing the shallow Siculo-Tunisian sill. This is a clear example of the difficulty deep-water Atlantic species may experience in spreading across the entire Mediterranean. Direct comparisons of biodiversity patterns between Mediterranean and other oceans' fauna are scarce. An example is the study by Massutí et al. [239] on fish fauna, comparing data from 20 years of trawling in the Atlantic and Mediterranean. The authors found significant differences in deep-sea fish abundance, species richness, and composition. Fish species richness was lower in the Mediterranean than in the deep Atlantic [239] and this has also been observed for other faunal groups such as gastropods [113], asteroids [243], and Asellota isopods (see Text S1 for more references).

### Comparative analysis of the deep-sea hot spots of biodiversity in the Mediterranean Sea

The presence of certain habitats, such as submarine canyons, cold-water corals, or cold seeps, can provide additional information about environmental factors that influence the abundance and distribution of deep-sea benthic species (Figure 4). However, a comparative analysis of the benthic diversity across different ecosystem types is difficult because of the different amount of data collected in different habitats and the heterogeneous focus on different taxa in each system. In the present study, we attempted to compare the biodiversity associated with open slopes, canyons, deep-water coral habitats, seamounts (i.e., in the sediments surrounding the seamount), and deep basins. To allow a more homogenous comparison, we considered only the foraminiferal, Nematoda (for Meiofauna), macrofaunal diversity expressed as ES(100), and megafaunal diversity expressed as species richness. Deep-sea canyons, for instance, can act as essential habitats for certain megafaunal species, which find a suitable environment for feeding, reproduction, and growth, often related to the increased availability of organic matter due to the enhanced transport of particles from the shelf down the canyon [26], [44], [46], [48], [154], [157], [244], [245]. This is confirmed by data in Figure 4 that show the significantly higher megafaunal diversity in deep-sea canyons of the Mediterranean than on open slopes. This pattern apparently does not hold for foraminiferal and meiofaunal diversity, which were equivalent in slopes and canyons (ANOVA, *p* not significant). However, all animal components investigated displayed significantly lower values in the deep basin than in slopes and canyons (ANOVA, *p*<0.01). For cold-water corals, the complex structure provided by the frame-building species provides refuges for many species and increases habitat heterogeneity, creating a suitable environment for recruitment and growth of many other species. This is confirmed by the large number of megafaunal species (comparable to that of slopes) and by the extremely high values of meiofaunal (as Nematoda) diversity (as ES(100)), which displayed significantly higher values in coral systems than in any other ecosystem type. A proper comparison for seamounts is difficult because Meiofauna and Macrofauna have been not investigated systematically in these habitats. However, comparing the sediments surrounding the bases of the seamounts with those of all other systems, the lowest values can be observed, probably a result of the turbulence and hydrodynamics associated with the seamount. In cold seeps, the trophic structure is completely different, as here there is primary production from chemoautotrophic Bacteria, which fuel the benthic community with a supplementary and continuous food source not found in the heterotrophic deep-sea ecosystems. Data available so far from the Mediterranean are too limited to make a comparison, but the species richness is likely to be lower than in any other system.

### Analysis of the known: How many species in the deep Mediterranean Sea?

**Figure 5:**
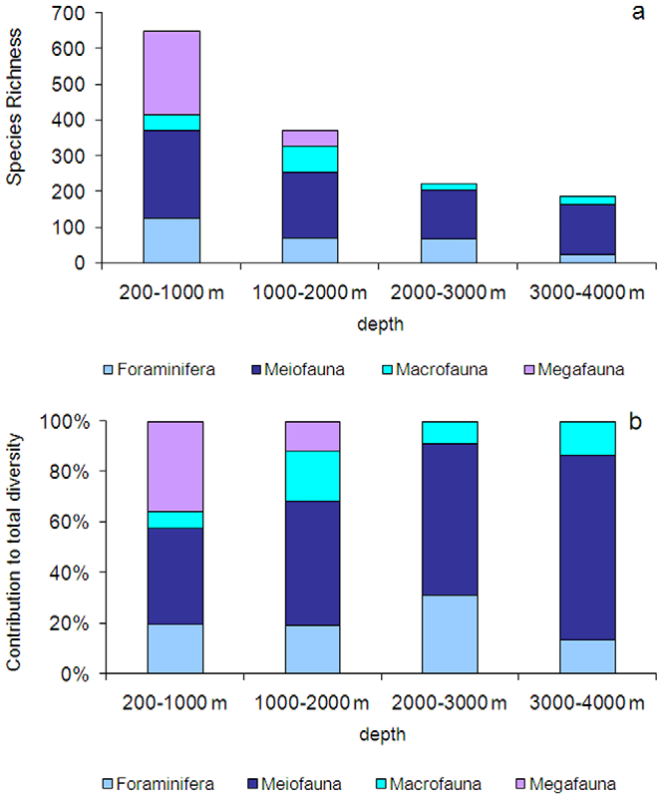
Apparent contribution of different benthic components to global biodiversity in the deep Mediterranean Sea. Reported are (a) sum of the number of species of Foraminifera (as live specimens), Meiofauna (as Nematoda), Macrofauna, and Megafauna, and (b) relative contribution of the different benthic components to the total diversity (expressed as percentage). Note that data for megafauna beneath 2,000 m depth are not available.

Despite the number of kingdoms in the deep sea being smaller than in coastal systems because of the absence of photoautotrophic taxa, there is no deep-sea area or station where the total biodiversity (i.e., the biodiversity of all forms of life ranging from Bacteria and Archaea to Megafauna) has been censused. We made a first attempt to quantify the total deep-sea diversity on the basis of the species identified so far for the Foraminifera, Nematoda (for Meiofauna), Macrofauna, and Megafauna (Figure 5). Within the bathymetric range of 200–1,000 m, approximately 650 species belonging to the Eukarya domain have been encountered, and Megafauna and Nematoda contributed almost equally to total biodiversity, while Foraminifera and Macrofauna contributed to a lesser extent (Figure 5). The total number of species decreased by almost half moving to the bathymetric range of 1,000–2,000 m, with a contextual increase of the meio- and macrofaunal contribution to the overall biodiversity. Deeper than 2,000 m, the global biodiversity was further reduced by about 40%, with a notable increase of the relative importance of the foraminiferal (20–30%) and meiofaunal diversity (60–80%). Table 1 illustrates the present state of knowledge of deep-sea biodiversity encountered from 200 m to more than 4,000 m depth in the entire Mediterranean basin. The values reported here are certainly an underestimate, not only because of the large number of still undiscovered species (see below) but also because the diversity of most phyla (e.g., Nemertea, Gnathostomulida, Kinorhyncha, Loricifera, Rotifera, Gastrotricha) has not been determined. Data reported here highlight the presence of clear differences in knowledge of the components of the deep-sea biota. Such differences are evident in the fragmented spatial coverage of the investigations, and it is clear that the claims that “*the different parts of the deep Mediterranean have not been equally sampled*” [107], and that “*the relative species richness of … faunas of the different sectors of Mediterranean is better correlated with the level of research effort than the true species richness*” [246] still hold true after 20 years of intensive deep-sea research.

**Table 1:** Taxonomic classification of species reported in the deep-sea sediments (from 200 to >4,000 m depth) of the Mediterranean Sea.

Taxonomic group	No. species	State of knowledge(1)	No. introduced species	No. experts	References
**Domain Archaea**	35 OTUs g^−1^ (2)	Scant	Not available	na	[97], [100], [193], [278]
**Domain Bacteria** (including Cyanobacteria)	1306 OTUs g^−1^ (3)	Scant	Not available	na	[88], [89], [95]–[97], [100], [193], [278]
**Domain Eukarya**					
**Kingdom Chromista**					
Phaeophyta	na	-	-	-	-
**Kingdom Plantae**	na	-	-	-	-
Chlorophyta	na	-	-	-	-
Rhodophyta	na	-	-	-	-
Angiospermae	na	-	-	-	-
**Kingdom Protoctista (Protozoa)**					
Dinomastigota (Dinoflagellata)	na	-	-	-	-
Foraminifera	197	68% unknown		na	[78], [84], [86, Pancotti unpubl.]
**Kingdom Animalia**					
Porifera	5	na		na	[26], [129], [108]
Cnidaria	2, 15	na			[72], [75], [129], [172], [179], [181], [182], [184], [279]–[284]
Platyhelminthes	na	na	na	na	
Mollusca	74	na	na	na	[26], [108], [129]
Annelida	18	na	na	na	[26], [108], [129]
Crustacea	149(4)	na	na	na	[26], [108], [129], [285], [286]
Bryozoa	2	na	na	na	[129]
Echinodermata	16	na	na	na	[26], [108], [129]
Urochordata (Tunicata)	3	na	na	na	[129]
Echiura	3	na	na	na	[129]
Sipunculida	6	na	na	na	[129]
Brachiopoda	1	na	na	na	[26], [108], [129]
Loricifera	3	na	na	na	[223]
Other invertebrates: Nematoda	345	80% unknown	na	na	[57], [59], [61]–[64], [68], [148], [170, Company unpubl.]]
Vertebrata (Pisces)	100	na	na	na	
Chondrichthya	8	na	na	na	[26], [115], [116], [123]
**SUBTOTAL**	947(5)				
**Benthic groups by size:**					
metazoan meiofauna		78% unknown			
macrofauna		76% unknown			
megafauna		42% unknown			
**TOTAL REGIONAL DIVERSITY**	2805				

Notes: na = not applicable, Scant = not evaluated in detail.

(1) The percentage of unknown species is the ratio between the total number of species estimated from the rarefaction curves and the number of species already described.

(2) Data of archaeal diversity are referred only to fingerprinting techniques and are largely underestimated.

(3) Data of bacterial diversity based on clone libraries, from a limited number of samples and spatial coverage.

(4) Only available species on deep-sea macrofauna (suprabenthic amphipods and cumaceans) and megafauna species (decapod).

(5) Total regional diversity including all taxonomic groups as reported in Table S1, S2, S3, S4, S5 (excluding prokaryotes).

### Analysis of the unknown biodiversity and identification of priorities for future discoveries in the deep Mediterranean

**Figure 6:**
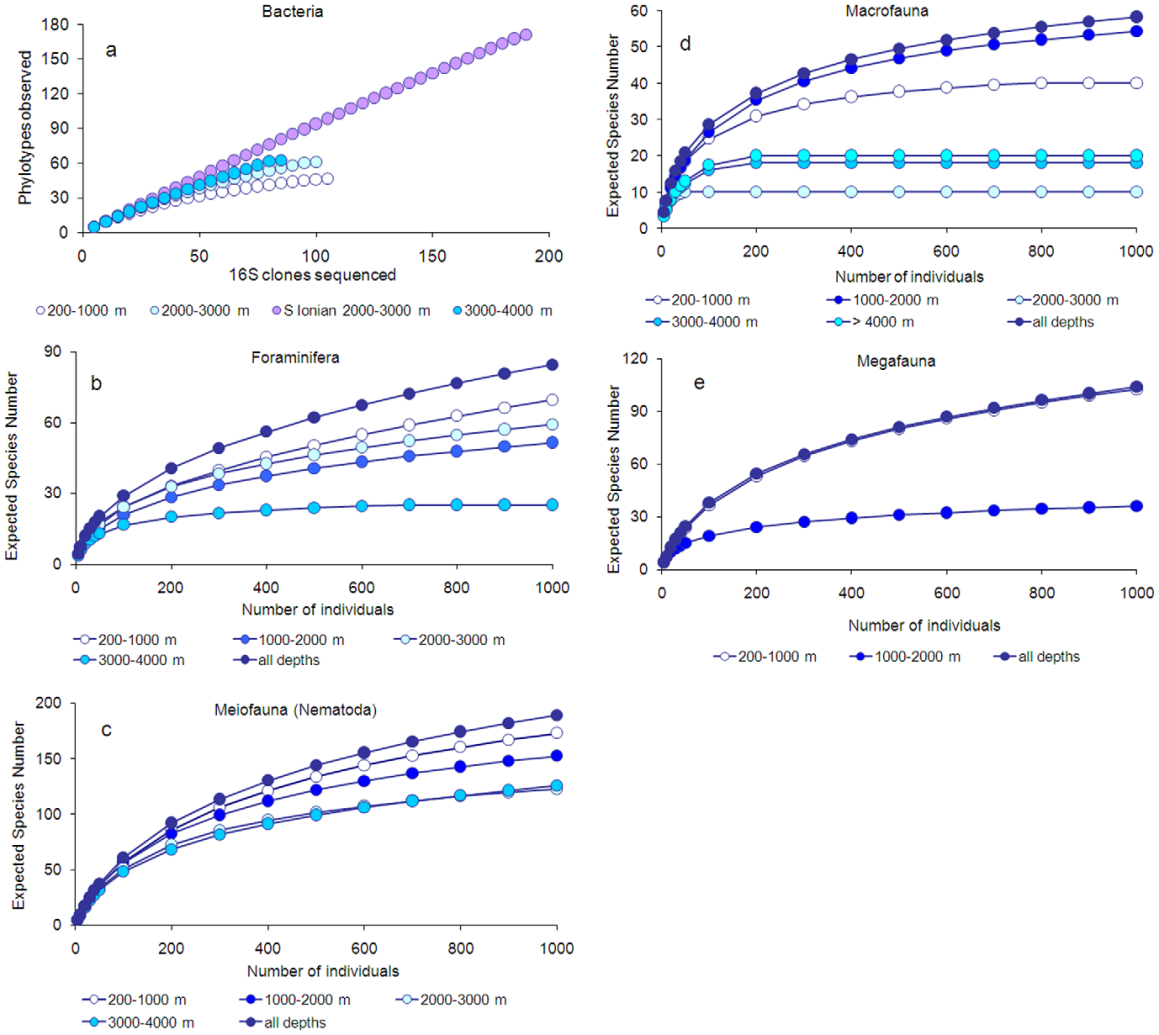
Rarefaction curves for the different components of the deep biota. The equations of the rarefaction curves are reported in Table S7.

One of the major unknowns in the deep Mediterranean is related to the quantification of the actual benthic microbial diversity. This includes Bacteria and Archaea, but to a large extent also the nanoflagellates and other protists (with the exception of Foraminifera). Although the last decades have seen a significant increase in projects sampling in the bathyal and abyssal Mediterranean, the areas covered and the number of samples are still limited. In the present study, we did not make an in-depth estimate of the potential microbial diversity of the deep-Mediterranean Sea, because different results can be obtained depending on the molecular technique used to measure microbial diversity. For instance, using a fingerprinting technique (ARISA), the number of total deep-sea bacterial species could be close to 4,000, but the same calculation based on the rarefaction curves obtained from clone libraries (Figure 6a) would give much higher diversity.

**Figure 7:**
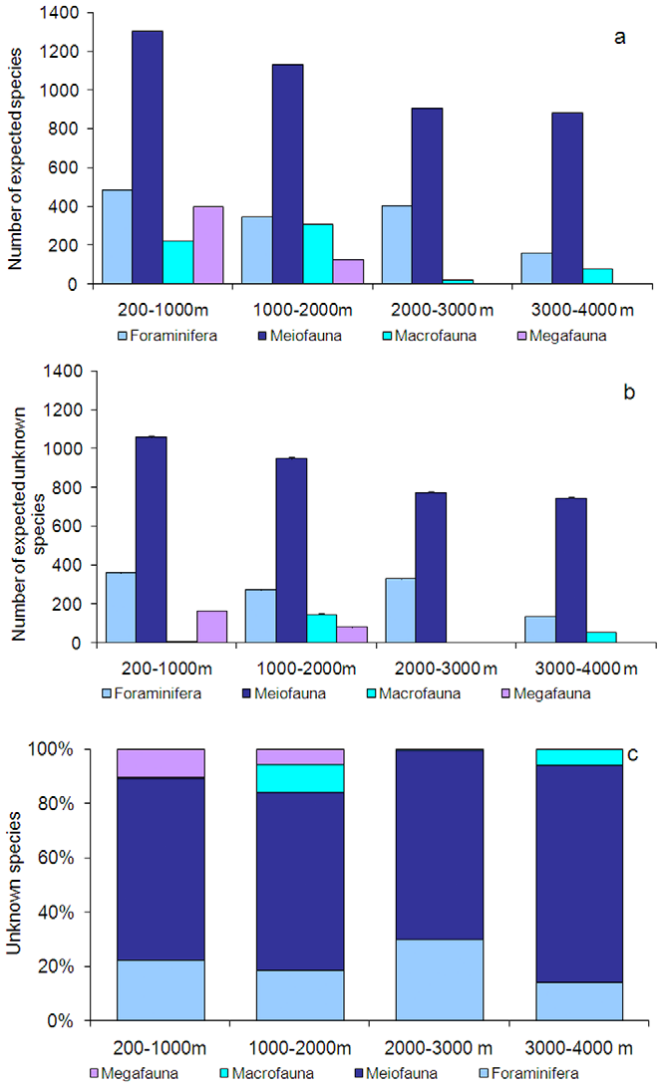
Expected number of species for each deep-fauna component within the sea bottom extension of each depth interval. Reported are (a) total number of expected species, (b) total number of unknown expected species, and (c) the relative contribution of the unknown expected species on the total diversity for Foraminifera, Meiofauna (as Nematoda), Macrofauna, and Megafauna. The expected number of species for each component has been estimated using the equations of the rarefaction curves reported in the caption of Figure 6. Details on the estimates of area per bathymetric range and the average abundance of each component are summarized in Table S6.

Using the equations derived from the rarefaction curves reported in Figure 6 (b–e) for the different animal groups and quantifying the abundance of each component per square meter at each bathymetric range and the areal extension of each depth range (Table S6), we attempted to estimate the potential number of species hosted by deep-sea sediments of the Mediterranean (the equations of the rarefaction curves are reported in Table S7). The results illustrated in Figure 7 indicate that at all depths the largest number of expected species is for Nematoda (Meiofauna), followed by Foraminifera, Megafauna (particularly in the range 200–2,000 m), and Macrofauna. We also compared these data with the number of species currently known for each bathymetric range and estimated the number of potentially unknown species for each faunal group. According to the patterns described above, the largest number and fraction of unknown diversity lie within the meiofaunal size (Foraminifera and Nematoda), but a significant number of undiscovered species are also expected within the megafaunal and macrofaunal components (approximately 200 and 270 species, respectively; Figure 7). These estimates are subject to a large degree of uncertainty because of the problems in determining accurate values of abundance of all groups in all sampling ranges and in the error associated with each equation derived from rarefaction curves. However, if estimates reported here for the investigated animal groups represent the actual proportion between known and unknown diversity, it could be concluded that approximately 66% (947 over 2,805 species expected) of the total deep-sea Mediterranean diversity remains undiscovered (Table 1).

The rate of new species discovery within the phylum Nematoda in the last 15 years has been about 15 species per year [247]. Assuming this rate of discovery, we would need 70 to 235 years just to complete a census of the deep-sea Mediterranean nematodes.

Experience suggests several points to be considered in future research to advance our knowledge of the biodiversity and ecosystem function of the deep benthic Mediterranean. For topographically isolated habitats such as deep-water corals, cold seeps, and submarine canyons, their irregular presence along the continental margin of the Mediterranean suggests the need for studies aimed at understanding the connections among interspersed systems as well as the importance of the integrity of each system to the sustainable functioning and biodiversity of adjacent systems.

Molecular studies of basin-isolated populations of benthic species would advance our understanding of their history and trace how they were affected by the cataclysms that have been part of the history of the deep Mediterranean. Such studies could, in turn, help us to understand the impact of the sapropelic conditions described in some catastrophic scenarios such as landslides”. The “impoverished” populations of the Mediterranean deep sea are in fact able survivors or agile colonizers. Facing a future of global perturbations, we would do well to study them.

Future priorities for deep-sea research in the Mediterranean include fine-scale analysis of the interactions between spatial heterogeneity at different scales and deep-sea biodiversity. Are the mosaic distribution of deep-sea biodiversity and the interaction of biotic and abiotic processes different at different spatial scales? Are the components of biodiversity contributing in the same way to deep-sea ecosystem functioning (e.g., ecological efficiency in exploiting available resources)? Is the loss of a specific benthic component harmful to the biodiversity of other benthic components? Such information for deep-sea benthos is clearly a primary issue for understanding deep-sea ecosystem functioning.

### Major threats to deep-sea biodiversity in the Mediterranean Sea

When settled on the seafloor, litter alters the habitat, either by furnishing hard substrate where none was available before or by overlying the sediment, inhibiting gas exchange, and interfering with life on the seabed. This is a persistent, but overlooked, problem for marine ecosystems worldwide, and its potential as a hazard for marine biota has been acknowledged only in recent decades [248]. It is of even greater importance in the land-enclosed Mediterranean Sea with its intensive shipping activity. In 1975, estimates of vessel-generated refuse discarded into the Mediterranean, based on 1964 shipping data, were close to 325,000 t. In the decades since, the number of mercantile vessels sailing in the waters of the Mediterranean has increased dramatically in 2006, 13,000 merchant vessels made 252,000 calls at Mediterranean ports and an additional 10,000 vessels transited through the sea. It is reasonable to suppose that litter input from vessels has increased as well. Studies of marine litter in the Mediterranean include surveys of seabed debris on the continental shelf, slope, and bathyal plain [249]–[251]. In most studies, plastic items accounted for much of the debris, sometimes as much as 90% or more of the total, owing to their ubiquitous use and poor degradability. A survey of seabed litter at depths ranging from 194 m to 4,614 m, from the Gulf of Taranto to the southeastern Levant, showed that the most common litter items were paint chips (44%) and plastics (36%). The presence of paint chips in half of the sites surveyed indicates that much of the litter originated from shipping. Most litter items were nonbuoyant objects such as glass and metal that probably sank in place [249].

Munitions and bombs have been also discharged at sea, especially during activities in Kosovo, and their dumping in open waters contributes to seafloor contamination. Another major threat to the benthic fauna is the presence of lost or discarded fishing gear, such as nets and longlines, which continue ghost fishing and can damage fragile ecosystems such as cold-water corals.

Chemical contaminants such as persistent organic pollutants, toxic metals (e.g., Hg, Cd, Pb, Ni), radioactive compounds, pesticides, herbicides, and pharmaceuticals are also accumulating in deep-sea sediments [252]. Topography (e.g., presence of canyons) and hydrography (e.g., cascading events) play a major role in the transportation and accumulation of these chemicals from the coast and shelf to the deep basins, affecting the local fauna. Recent studies have detected the presence of significant levels of dioxins in the commercial shrimp *Aristeus antennatus* [253] and significant levels of persistent organic pollutants in mesopelagic and bathypelagic cephalopods [254].

The thermohaline circulation oxygenates the deep and bottom layers in the Mediterranean. This vertical circulation is forced by the deep-water formation processes occurring under favorable meteorological conditions in the Gulf of Lions and the northern Adriatic [255], [256]. However, events in the past 20 years demonstrated the instability of the process. An abrupt change in hydrography and large-scale circulation of the deep waters of the Eastern Mediterranean resulted from a unique, high-volume influx of dense waters from the Aegean Sea during the 1990s. The event, named “Eastern Mediterranean Transient” (EMT) [257], caused significant changes in deep-sea biodiversity [258]. Extreme scenarios of climate change predict changes in the site of deep-water formation and a weakening of thermohaline circulation, which could result in changes in the oxygenation and biogeochemical cycles in the bottom layers of the deep Mediterranean Sea [148]. Recently, episodic or catastrophic events have been described as one of the main environmental contributors to faunal disturbance and thus one of the main potential drivers of deep-sea biodiversity [225], [259]. Limited information is available, but the effects of these episodic events on the deep Mediterranean Sea appear relevant (Cap de Creus Canyon, Western Mediterranean) [15], [260]. An important ecological effect on the maintenance of *Aristeus antennatus* populations in the northwestern Mediterranean has been linked to the episodic events of dense water cascading on the Gulf of Lions [260]. These events are climate-driven processes, and therefore climate change will have an impact on the frequency and intensity of cascading, with unknown effects on the benthic fauna. Another potential effect of climate change is related to energy transport from surface waters to the seafloor [261], [262]. Primary production will change in the surface layers according to sun exposure, water temperature, major stratification of water masses, for example, and this will affect the food chain down to the deep seafloor, which will be subject to differences in quantity, quality, and timing of organic matter input. Also, recent years have seen an increase in gelatinous organisms, which, when they sink, result in an important transport of energy to the deep sea. This can have significant implications for certain species, such as the fish *Alepocephalus rostratus*, which feeds mainly on gelatinous organisms. Its populations form more than 60% of the megafaunal biomass at the deep continental margins of the western and central Mediterranean basins.

Finally, the Mediterranean supports important and increasing commercial fishing activity, which is entering deeper waters as the shallower resources are depleted. For example, the commercial fleet of the Catalan Sea has exploited the rose shrimp *Aristeus antennatus* for over six decades and is now fishing at depths of about 900 m. Little is known about the effects of deep-water trawling on benthic fauna and habitat. Pioneer studies have shown that intense commercial trawling may trigger sediment gravity flows with an increase in near-bottom turbidity of one order of magnitude, an increase in current velocity of two to four times, and an increase in horizontal sediment fluxes of one to three orders of magnitude [263], [264]. The effects on the fauna, however, are unknown and need further investigation. Previous research and joint efforts of the World Wildlife Fund and the International Union for Conservation of Nature have led to the ban on trawling below 1,000 m [25], making the deep benthic Mediterranean the largest protected area in the world. Such precaution is of major importance, as it protects an ecosystem that is mostly unknown. Nevertheless, this situation needs to be monitored and managed. Future research is essential to advance our understanding of the biodiversity and ecosystem function of the deep Mediterranean and to provide sound scientific data that enable policy makers and stakeholders to develop conservation and management options.

## Methods

### Prokaryotic diversity

Prokaryotic diversity has been investigated using molecular approaches that include a wide range of techniques, among them fingerprinting methods such as ARISA or T-RFLP, which reflect the richness and community composition of the dominant components of the assemblage in large sets of samples [88], [100], [265] and the cloning and sequencing of 16S rRNA genes, which also provide information on the phylogenetic identity of dominant members [96], [193]. ARISA and T-RFLP analyses were carried out as described, respectively, by Danovaro et al. [97] and Luna et al. [88], [100]. Clone libraries were created from bacterial 16S ribosomal RNA genes amplified by PCR (polymerase chain reaction) with the universal bacterial primers 27f (modified to match also Planctomycetales; 5′-AGRGTTTGATCMTGGCTCAG-3′) and 1492r (5′-GGYTACCTTGTTACGACTT-3′); details are provided in [89], [95], [96]. The obtained sequences were used for phylogenetic analysis with the ARB software. The extracts were further used for sequencing. Similarity matrices among the sequences of the clones were calculated to identify the operational taxonomic units (OTUs) that were further used to estimate species richness (Chao-1) using the Web-based rarefaction calculator software (http://www2.biology.ualberta.ca./jbrzusto/rarefact.php).

### Foraminiferal diversity

A variety of sampling gear has been used to collect samples for the study of deep-sea Foraminifera [266]. Earlier studies were based on samples obtained using grabs, gravity cores, or piston cores, which do not retain the surface sediment where living Foraminifera are concentrated [76], [78], [80] (see Text S1 for additional references). Subsequently, box cores have been used [79], [86], [267]. Recent studies have been based on high-quality multicorer samples [82]–[84]. Pancotti (unpublished) included soft-shelled monothalamous species in her study of samples from the Eastern and Western Mediterranean. All other authors have confined themselves to hard-shelled species and therefore have not encompassed the full range of foraminiferal biodiversity in the deep Mediterranean. Some important papers [87] only report counts for selected species. In early studies, samples were not treated with Rose Bengal and therefore yielded “total” assemblages, that is, a mixture of “live” and “dead” tests. We have included some such studies [78], [80] because they are particularly relevant to this synthesis. All other samples were stained with Rose Bengal to distinguish between Foraminifera that were alive when collected and those that were dead. Sieve mesh size is a crucial variable that strongly influences assemblage composition. In the Mediterranean the following meshes have been used: 32, 63, 125, and 150 µm. A final point to consider is that geologists, who published most of the data available, are less interested in diversity than biologists, and species lists are therefore often incomplete or do not differentiate species in “difficult” genera such as *Fissurina*, *Lagena*, *Bolivina*, *Brizalina*, and *Lenticulina*. The outcome of these predominately geologically orientated studies is an inconsistent body of data that cannot be easily integrated to produce an overall synthesis of community parameters. We therefore focus our analyses mainly on data from single papers.

### Meiofaunal diversity

The dataset on nematode diversity (Species Richness) consists of 161 samples (new and literature data) collected with multicores and box cores in different ecosystems (open slope, canyon, rise, seamount, and deep basin) along the entire deep Mediterranean Sea from the western to the eastern basin at depths ranging from 204 m to 4,000 m [57], [59], [61], [64], [68], [148], [170]. Data on nematode species composition were obtained from a subset of 143 samples. For diversity, Meiofauna were extracted according to the standard protocols. All the meiobenthic animals were counted and classified per taxon under a stereomicroscope after staining with Rose Bengal (0.5 g L^−1^). For Nematoda identification, specimens were mounted on slides (following the formalin-ethanol-glycerol technique to prevent dehydration) and identified to species level according to the recent literature dealing with new nematode genera and species (see Text S1 for more references).

### Macrofaunal diversity

Deep-sea Macrofauna has been typically sampled using a modified Agassiz benthic trawl (2.3 m wide and 0.9 m high), a 14.76 m Marinovich-type deep-water trawl (codend mesh 6 mm) with a 0.5 mm plankton net secured on top, a sled for suprabenthic Macrofauna, and different types and sizes of box corers, depending on the depth considered and the research teams. A 0.062 m^2^ box corer with an effective penetration of 40 cm (Ocean Instruments model 700 AL) has been used in the Levantine Sea. The samples are typically preserved in 10% buffered formalin aboard ship. In the laboratory, samples were washed and sieved through 250 µm mesh.

### Megafaunal diversity

Deep-sea Megafauna has been sampled in the Western Mediterranean by different methods, depending on the depth considered. Slope Megafauna has been sampled from commercial trawlers using bottom otter trawls down to 700–800 m depth. These commercial trawls have horizontal mouth openings of 20–25 m and 3–5 m of vertical opening, with a 40 mm stretched mesh in the codend liner, and are trawled over the seafloor at about 3 knots [48], [134]. Rucabado et al. [268] were the first researchers to use the otter semiballoon trawl gear (OTSB: 8 m horizontal spread and 0.8 vertical mouth opening) in the Mediterranean. This sampling device was subsequently transformed into the otter trawl Maireta System (OTMS: 12 m horizontal spread and 1.4 m vertical opening approximately) [54]. The OTMS is equipped with SCANMAR sensors that provide information on bottom contact time and vertical and horizontal opening of the trawl's mouth down to 1,500 m depth, allowing calculation of sampled area [47]–[49], [108], [115], [116], [128], [129]. Furthermore, the Agassiz trawl has been commonly used to sample the deep Western and Eastern Mediterranean benthos since the late 1980s [50], [53], [71]. In the Balearic Sea, approximately 350 hauls have been made, covering no more than 7–8 km^2^ over an area of about 9,000 km^2^ (i.e., only 0.08% of the Balearic slope below 1,000 m has been directly sampled, 40% after year 2000). A total of 174 trawl hauls from a series of 24 cruises conducted between 1988 and 2004 off the coast of Israel, at depths between 720 and 1,558 m, were analyzed. The samples were collected aboard the RV *Shikmona* (720 HP; 27 m), using a modified Agassiz trawl (2.3 m width and 0.9 m height), a 14,76 m deep-water trawl (Marinovich-type, codend mesh 6 mm) with a 0.5 mm plankton net secured on top.

Deep-water Megafauna species have been collected in the central-eastern Mediterranean since the *Pola*, *Thor*, and *Dana* (see Text S1 for more references) expeditions. An important contribution to our knowledge of Megafauna was provided by professional fishing and further explorations using dredge and trawl [34] (see Text S1 for more references). Most data on the slope Megafauna were acquired using bottom otter trawl gear down to 700–800 m depth during Italian GRUND [269] and international MEDITS [270] study projects carried out since 1985 in the Italian seas and 1994 in the northern Mediterranean, respectively. Commercial motor-powered vessels, equipped with an otter trawl net, with stretched mesh of 40 mm in the codend, were hired during GRUND surveys, while a specially designed net with a stretched mesh of 20 mm in the codend was used during the MEDITS cruises. The collection of information on the Megafauna in waters deeper than 800 m using otter trawl gears has been carried out during some EU and regional projects. In particular, during the EU-DESEAS project, sampling was conducted with the otter trawl Maireta System (OTMS) using the RV *Garcia del Cid* (1,500 HP, 38 m; [46]). During INTERREG Italy-Greece, a depth range between 300 m and 1,200 m was examined using two hired commercial trawlers equipped with bottom trawl net with a codend mesh size of 40 mm (stretched) [117], [271]–[273]. During the EU-RESHIO project a commercial bottom trawler towing an Italian-type fishing net of 40 mm (stretched) was used. The sampling design was randomly stratified by depth between 300 m and 900 m [271], [274]. During the regional project GAVIS, the sampling was conducted using a professional motor-powered vessel equipped with an experimental otter trawl Maireta net, used with double warps. The stretched mesh in the codend was 20 mm. The sampling design adopted was random-stratified according to the following depth strata: 400–600 m; 600–800 m; 800–1,000 m; 1,000–1,200 m. The hauls were allocated in each depth stratum in proportion to their surface area [275]. During the regional Spanish project RETRO, sampling of Megafauna was conducted using the OTMS [44], [129], and during the regional Spanish project RECS, sampling was conducted using multicores for Meiofauna, epibenthic sledge for suprabenthos Macrofauna, and OTMS for Megafauna [48].

### Diversity metrics

The diversity of the different components was reported as (a) Species Richness (SR), the total number of species or operational taxonomic units (OTUs) identified in each sample, (b) Shannon-Wiener information function (H′, using log base 2), and (c) Margalef's index: (*D* = (*S*−1)/ln *N*), where *S* is the number of species and *N* is the number of individuals in the sample. To standardize the values of diversity estimated using a different number of individuals [276], the species-abundance data were used to calculate rarefied species richness ES(51 and 100) as the expected number of species for a theoretical sample of 51 and 100 specimens, respectively [6], [8], [61], [62], [170]. The equitability of benthic assemblages was estimated as Pielou's index (evenness J′). The turnover diversity (as % Bray-Curtis dissimilarity; [277]) was estimated as the dissimilarity in species composition at different depths and longitudes toward the SIMPER analysis (based on the Bray-Curtis similarity index). ANOSIM analysis was used to test the presence of statistical differences in the species composition among different assemblages. SIMPER and ANOSIM analyses were performed using PRIMER v5 (Plymouth Marine Laboratory, UK).

### Meta-analyses

The meta-analyses, performed on the entire dataset of this synthesis, were based on two diversity indices: Species Richness and the Expected Species Number for 100 individuals estimated for each component (data for Prokaryotes, Foraminifera, Meiofauna, Macrofauna and Megafauna are summarized in Tables S1, S2, S3, S4, S5 and in [43]). Since species richness is strongly affected by the sample size, to standardize the values of diversity estimated for each benthic component using different sampling efforts, the expected number of species for a theoretical sample of 100 specimens (ES(100)) was selected. Only for bacterial and archaeal OTU richness data, the ES(100) was not estimated due to the fact that it is not possible to convert OTU richness data in ES(100). All data for Foraminifera, Meiofauna, Macrofauna, and Megafauna have been standardized using the rarefaction curves in which the same number of specimens were used to estimated the diversity for each benthic component. For Prokaryotes, the rarefaction curves were estimated only for diversity data obtained using 16S rDNA sequences. The total number of expected species, the total number of unknown expected species, and the relative contribution of the unknown expected species on the total diversity for Foraminifera, Meiofauna (as Nematoda), Macrofauna, and Megafauna were estimated using the equations of the rarefaction curves, whereas the details on the estimates of area per bathymetric range and the average abundance of each component were summarized in the supporting information.

## Supporting Information

Table S1 Data of prokaryotes biodiversity. Reported are: location, station, habitat, latitude (Lat), longitude (Long), depth, sampling gear (BC for box corer and MC for multicorer), method for analysis (C: cloning and F: fingerprinting), bacterial Richness (BR), archaeal Richness (AR), and references.
(0.12 MB DOC)

Table S2 Data of foraminiferal biodiversity. Reported are: location, sampling period, habitat, station, latitude (Lat), longitude (Long), depth, sampling gear (GC for gravity corer, G for grab, PC for piston corer, BC for box corer, MC for multicorer), type of assemblage A (D: dead; L/S: live and stained), Species Richness (SR), number of individuals (N), ES(51), Shannon index (log base 2), Simpson (1−λ), and references included in Text S2.
(0.44 MB DOC)

Table S3 Data of nematodes biodiversity. Reported are: location, sampling period, habitat, station, latitude (Lat), longitude (Long), depth, sampling gear (BC for box corer and MC for multicorer), Species Richness (SR) and Genus Richness (values reported in red), ES(51), Shannon index (H′, log base 2), Margalef index (D), Pileou index (J) and references included in Text S2. Red values are referred to genus level.
(0.48 MB DOC)

Table S4 Data of megafauna biodiversity. Reported are: location, sampling period, habitat, station, latitude (Lat), longitude (Long), depth, sampling gear (trawl: c for commercial or OTMS), Species Richness (SR), number of individuals (N), Margalef index (D), Pielou index (J), ES(51), Shannon index (H′), Simpson (1−λ) and references included in Text S2.
(0.19 MB DOC)

Table S5 Benthic megafauna and macrofauna sampled on the Eastern Mediterranean seeps.
(0.13 MB DOC)

Table S6 Data on the extension of the sea bottom at the selected depth interval and average abundance of Foraminifera, Meiofauna (as Nematoda), Macrofauna, and Megafauna.
(0.04 MB DOC)

Table S7 The equations of the rarefaction curves reported in Figure 6(a–e).
(0.02 MB DOC)

Text S1 Additional references.
(0.04 MB DOC)

Text S2 References included in the additional tables.
(0.03 MB DOC)

## References

[1] Gage JD, Tyler PA (1991). Deep sea biology: A natural history of organisms at the deep-sea
floor.

[2] Snelgrove PVR (1999). Getting to the bottom of marine biodiversity: Sedimentary
habitats.. BioScience.

[3] Grassle JF, Maciolek NJ (1992). Deep-sea species richness: Regional and local diversity estimates
from quantitative bottom samples.. Am Nat.

[4] Etter RJ, Grassle JF (1992). Patterns of species diversity in the deep sea as a function of
sediment particle size diversity.. Nature.

[5] Blake JA, Grassle JF (1994). Benthic community structure on the US South Atlantic slope off
the Carolinas: Spatial heterogeneity in a current-dominated
system.. Deep Sea Res II.

[6] Gambi C, Vanreusel A, Danovaro R (2003). Biodiversity of nematode assemblages from deep-sea sediments of
the Atacama Slope and Trench (Southern Pacific Ocean).. Deep Sea Res I.

[7] Lambshead PJD, Tietjen J, Ferrero T, Jensen P (2000). Latitudinal diversity gradients in the deep-sea with special
reference to North Atlantic nematodes.. Mar Ecol Progr Ser.

[8] Lambshead PJD, Brown CJ, Ferrero T, Mitchell NJ, Smith CR (2002). Latitudinal diversity patterns of deep-sea marine nematodes and
organic fluxes: A test from the central equatorial Pacific.. Mar Ecol Progr Ser.

[9] Levin LA, Gage JD, Martin C, Lamont PA (2000). Macrobenthic community structure within and beneath the oxygen
minimum zone, NW Arabian Sea.. Deep Sea Res II.

[10] Rex MA, Stuart CT, Hessler RR, Allen JA, Sanders HL (1993). Global-scale latitudinal patterns of species diversity in the
deep-sea benthos.. Nature.

[11] Gooday AJ, Bett BJ, Shires R, Lambshead PJD (1998). Deep-sea benthic foraminiferal diversity in the NE Atlantic and
NW Arabian sea: A synthesis.. Deep Sea Res II.

[12] McClain CR, Etter RJ (2005). Mid-domain models as predictors of species diversity patterns:
bathymetric diversity gradients in the deep sea.. Oikos.

[13] Sardà F, Calafat A, Flexas MM, Tselepides A, Canals M (2004). An introduction to Mediterranean deep-sea
biology.. Sci Mar.

[14] Vanney JR, Gennesseaux M, Stanley DJ, Wezel F-C (1985). Mediterranean seafloor features: Overview and
assessment.. Geological evolution of the Mediterranean Basin.

[15] Canals M, Puig P, Durieu de Madron X, Heussner S, Palanques A (2006). Flushing submarine canyons.. Nature.

[16] Stanley DJ, Wezel FC (1985). Geological evolution of the Mediterranean basin.

[17] Emig CC, Geistdoerfer P (2004). The Mediterranean deep-sea fauna: Historical evolution,
bathymetric variations and geographical changes, Carnets de
Géologie/Notebooks on Geology, Maintenon, Article 2004/01
(CG2004_A01_CCE-PG).

[18] Danovaro R, Dinet A, Duineveld G, Tselepides A (1999). Benthic response to particulate fluxes in different trophic
environments: A comparison between the Gulf of Lions-Catalan Sea (Western
Mediterranean) and the Cretan Sea (Eastern Mediterranean).. Progr Oceanogr.

[19] Psarra S, Tselepides A, Ignatiades L (2000). Primary productivity in the oligotrophic Cretan Sea (NE
Mediterranean): Seasonal and interannual variability.. Progr Oceanogr.

[20] Tselepides A, Papadopoulou N, Podaras D, Plaiti W, Koutsoubas D (2000). Macrobenthic community structure over the continental margin of
Crete (South Aegean Sea, NE Mediterranean).. Progr Oceanogr.

[21] Yacobi YZ, Zohary T, Kress N, Hecht A, Robarts RD (1995). Chlorophyll distribution throughout the southeastern
Mediterranean in relation to the physical structure of the water
mass.. J Mar Syst.

[22] Krom MD, Kress N, Brenner S, Gordon LI (1991). Phosphorus limitation of primary productivity in the Eastern
Mediterranean.. Limn Oceanogr.

[23] Myers N, Mittermeier RA, Mittermeier CG, da Fonseca Gustavo AB, Kent J (2000). Biodiversity hotspots for conservation
priorities.. Nature.

[24] Bianchi N, Morri C (2000). Marine biodiversity of the Mediterranean Sea: Situation, problems
and prospects for future research.. Mar Poll Bull.

[25] WWF/IUCN, World Wildlife Fund/International Union for Conservation of
Nature (2004). The Mediterranean deep-sea ecosystems: An overview of their diversity,
structure, functioning and anthropogenic impacts.

[26] Ramirez-Llodra E, Company JB, Sardà F, Rotllant G (2009). Megabenthic diversity patterns and community structure of the
Blanes submarine canyon and adjacent slope in the Northwestern
Mediterranean: A human overprint?. Mar Ecol.

[27] Coll M, Piroddi C, Kaschner K, Ben Rais Lasram F, Steenbeek J (2010). The biodiversity of the Mediterranean Sea: Status, patterns and threats.. PLoS ONE.

[28] Forbes E (1844). Report on the Mollusca and Radiata of the Aegean Sea, and on
their distribution, considered as bearing on geology.. Report of the 13th British Association for the Advancement of Science,
London.

[29] Anderson TR, Rice T (2006). Deserts on the sea floor: Edward Forbes and his azoic hypothesis
for a lifeless deep ocean.. Endeavour.

[30] Risso A (1816). Histoire naturelle des Crustacés des environs de
Nice..

[31] Holthuis LB (1977). The Mediterranean decapod and stomatopod Crustacea in A.
Risso's published works and manuscripts.. Annales du Museum d'Histoire naturelle de Nice.

[32] Zugmayer E (1911). Poissons provenant des campagnes du yacht Princesse
Alice.. Résultats des Campagnes Scientifiques accomplies par le
Prince Albert I, Monaco.

[33] Geistdoerfer P, Rannou M (1972). Poissons benthiques récoltés en
Méditerranée occidentale par le N.O. Jean Charcot
(campagne Polymède).. Bulletin du Museum National d'Histoire Naturelle
Series.

[34] Klausewitz W (1989). Deep-sea and deep water fish of the Eastern Mediterranean,
collected during the METEOR-Expedition 1987.. Senckenb Marit.

[35] Pérès JM, Picard J (1958). Recherches sur les peuplements benthiques de la
Mediterranée Nord - Orientale.. Annales de l' Institute Océanographie Paris.

[36] Tchukhtchin VD (1964). Quantitative data on benthos of the Tyrrhenian
Sea.. Trudy Sevastopol Biological Station.

[37] Vamvakas C (1970). Peuplements benthiques des substrats meubles du sud de la Mer
Egée.. Tethys.

[38] Tselepides A, Eleftheriou A, Rowe GT, Pariente V (1992). South Aegean (Eastern Mediterranean) continental slope benthos:
macroinfaunal - environmental relationships.. Deep-sea food chains and the global carbon cycle.

[39] Koutsoubas D, Koukouras A, Karakassis I, Dounas C (1992). Contribution to the knowledge of Gastropoda and Bivalvia
(Mollusca) of Crete island (S. Aegean Sea).. Boll Malacol.

[40] Koutsoubas D, Tselepides A, Eleftheriou A (2000). Deep sea molluscan fauna of the Cretan sea (Eastern
Mediterranean): Faunal, ecological and zoogeographical
remarks.. Senckenb Marit.

[41] Karakassis J, Eleftheriou A (1997). The continental shelf of Crete: Structure of macrobenthic
communities.. Mar Ecol Progr Ser.

[42] Eleftheriou A, Smith CJ, Tselepides A (1996). Food Chains in the Aegean Sea..

[43] Kroncke I, Turkay M, Fiege D (2003). Macrofauna communities in the Eastern Mediterranean deep
sea.. P.S.Z.N. Mar Ecol.

[44] Sardà F, Cartes JE, Norbis W (1994). Spatio-temporal structure of the deep-water shrimp
*Aristeus antennatus* Risso, 1816 (Decapoda: Aristeidae)
population in the Western Mediterranean.. Fish B NOAA.

[45] Sardà F, Cartes JE, Company JB (1994). Spatio-temporal variations in megabenthos abundance in three
different habitats of the Catalan deep-sea (Western
Mediterranean).. Mar Biol.

[46] Sardà F, D'Onghia G, Politou C-Y, Tselepides A (2004). Mediterranean deep-sea biology.. Monographs Sci Mar.

[47] Sardà F, D'Onghia G, Politou CY, Company JB, Maiorano P (2004). Maximum deep-sea distribution and ecological aspects of
*Aristeus antennatus* (Risso 1816) in the Balearic and
Ionian Mediterranean Sea.. Sci Mar.

[48] Sardà F, Company JB, Bahamon N, Rotllant G, Flexas MM (2009). Relationship between environment and the occurrence of the
deep-water rose shrimp *Aristeus antennatus* (Risso, 1816) in
the Blanes submarine canyon (NW Mediterranean).. Progr Oceanogr.

[49] Sardà F, Company JB, Rotllant G, Coll M (2009). Biological patterns and ecological indicators for Mediterranean
fish and crustaceans below 1,000 m: A review.. Rev Fish Biol Fish.

[50] Galil BS, Goren M (1994). The deep sea Levantine fauna, new records and rare
occurrences.. Senckenb Marit.

[51] Goren M, Galil BS (1997). New records of deep-sea fishes from the Levant Basin and a note
on the deep-sea fishes of the Mediterranean.. Isr J Zool.

[52] Goren M, Galil BS (2002). On the occurrence of *Cataetyx laticeps* Koefoed,
1927 and *Ophidion barbatum* Linnaeus, 1758 in the Levant
Basin, Eastern Mediterranean, with a note on the deep sea fish community in
this region.. Cybium.

[53] Galil BS (2004). The limit of the sea: The bathyal fauna of the Levantine
Sea.. Sci Mar.

[54] Sardà F, Cartes JE, Company JB, Albiol T (1998). A modified commercial trawl used to sample the deep-sea
megabenthos.. Fish Sci.

[55] Golani D (1987). On deep-water sharks caught off the Mediterranean coast of
Israel.. Isr J Zool.

[56] Dinet A (1976). Etude quantitative du méiobenthos dans le secteur Nord
de la mer Egée.. Acta Adriatica.

[57] Vivier MH (1978). Influence d'un déversement industriel
profound sur la nématofaune (Canyon de Cassidaigne,
Méditerranée).. Téthys.

[58] de Boveé F, Guidi LD, Soyer J (1990). Quantitative distribution of deep-sea meiobenthos in the
northwestern Mediterranean (Gulf of Lions).. Cont Shelf Res.

[59] Soetaert K, Heip C, Vincx M (1991). Diversity of nematode assemblages along a Mediterranean deep-sea
transect.. Mar Ecol Progr Ser.

[60] Grémare A, Medernach L, de Bovée F, Amouroux JM, Vétion G (2002). Relationships between sedimentary organics and benthic meiofauna
on the continental shelf and the upper slope of the Gulf of Lions (NW
Mediterranean).. Mar Ecol Progr Ser.

[61] Danovaro R, Gambi C, Lampadariou N, Tselepides A (2008). Deep-sea nematode biodiversity in the Mediterranean basin:
testing for longitudinal, bathymetric and energetic
gradients.. Ecography.

[62] Danovaro R, Gambi C, Dell'Anno A, Corinaldesi C, Fraschetti S (2008). Exponential decline of deep-sea ecosystem functioning linked to
benthic biodiversity loss.. Curr Biol.

[63] Danovaro R, Canals M, Gambi C, Heussner S, Lampadariou N (2009). Exploring patterns and hot spots of benthic biodiversity on the
slopes of European margins.. Oceanography.

[64] Danovaro R, Bianchelli S, Gambi C, Mea M, Zeppilli D (2009). α-, β-, γ-, δ and
ε-diversity of deep-sea nematodes in canyons and open slopes of the
Northeast Atlantic and Mediterranean margins.. Mar Ecol Progr Ser.

[65] Guidi-Guilvard LD (2002). DYFAMED-BENTHOS, a long time-series benthic survey at 2347-m
depth in the northwestern Mediterranean: General
introduction.. Deep Sea Res II.

[66] Tselepides A, Lampadariou N (2004). Deep-sea meiofaunal community structure in the Eastern
Mediterranean: Are trenches benthic hot-spots?. Deep Sea Res I.

[67] Gambi C, Danovaro R (2006). A multiple-scale analysis of metazoan meiofaunal distribution in
the deep Mediterranean Sea.. Deep Sea Res I.

[68] Lampadariou N, Tselepides A (2006). Spatial variability of meiofaunal communities at areas of
contrasting depth and productivity in the Aegean Sea (NE
Mediterranean).. Progr Oceanogr.

[69] Gilat E, Gelman A (1984). On the sharks and fishes observed using underwater photography
during a deep-water cruise in the Eastern Mediterranean.. Fish Res.

[70] Priede IG, Bagley PM (2000). In situ studies on deep-sea demersal fishes using autonomous
unmanned lander platforms.. Oceanogr Mar Biol Annu Rev.

[71] Galil BS, Zibrowius H (1998). First benthos samples from Eratosthenes Seamount, Eastern
Mediterranean.. Senckenb Marit.

[72] Tursi A, Mastrototaro F, Matarrese A, Maiorano P, D'Onghia G (2004). Biodiversity of the white coral reefs in the Ionian Sea (Central
Mediterranean).. Chem Ecol.

[73] Taviani M, Freiwald A, Zibrowius H, Freiwald A, Roberts JM (2005). Deep coral growth in the Mediterranean Sea: An
overview.. Cold water corals and ecosystems.

[74] Taviani M, Remia A, Corselli C, Freiwald A, Malinverno E (2005). First geo-marine survey of living cold-water Lophelia reefs in
the Ionian Sea (Mediterranean basin).. Facies.

[75] Freiwald A, Beuck L, Rüggerberg A, Taviani M, Hebblen D (2009). The white coral community in the Central Mediterranean Sea
revealed by ROV surveys.. Oceanography.

[76] Massiotta R, Cita MB, Mancuso M (1976). Benthonic foraminifers from bathyal depths in the Eastern
Mediterranean.. Maritime sediments, Special publication.

[77] Wright WC, Rupert FP (1981). Late neogene and recent bathyal foraminifera of Mediterranean:
AAPG Bulletin,.

[78] Jorrisen FJ (1988). The distribution of benthic foraminifera in the Adriatic
Sea.. Utrecht Micropaleontological Bulletins.

[79] De Stigter HC (1996). Recent and fossil benthic foraminifera in the Adriatic Sea:
Distribution patterns in relation to organic carbon flux and oxygen
concentration at the seabed.. Geologica Ultraiectina.

[80] Parisi E (1981). Distribuzione dei foraminiferi bentonici nelle zone batiali del
Tirreno e del Canale di Sicilia.. Rivista Italiana di Paleontologia.

[81] Bizon G, Bizon JJ (1984). Les foraminifères des sediments
profonds.. Pétrole et Techniques.

[82] Schmiedl G, de Bovee F, Buscail R, Charrière B, Hemleben C (2000). Trophic control of benthic foraminiferal abundance and
microhabitat in the bathyal Gulf of Lions, Western Mediterranean
Sea.. Mar Micropaleontol.

[83] Heinz P, Kitazato H, Schmiedl G, Hemleben C (2001). Response of deep-sea benthic foraminifera from the Mediterranean
Sea to simulated phytoplankton pulses under laboratory
conditions.. J Foram Res.

[84] Fontanier C, Jorissen FJ, Lansard B, Mouret A, Buscail R (2008). Live (stained) foraminiferal faunas from open slope environments
separating submarine canyons in the Gulf of Lions (NW Mediterranean):
Diversity, density and microhabitats.. Deep Sea Res I.

[85] Cita MB, Zocchi M (1978). Distribution patterns of benthic foraminifera on the floor of the
Mediterranean Sea.. Oceanol Acta.

[86] De Rijk S, Troelstra SR, Rohling EJ (1999). Benthic foraminiferal distribution in the Mediterranean
Sea.. J Foram Res.

[87] De Rijk S, Jorissen FJ, Rohling EJ, Troelstra SR (2000). Organic flux on bathymetric zonation of Mediterranean benthic
Foraminifera.. Mar Micropaleontol.

[88] Luna GM, Dell'Anno A, Giuliano L, Danovaro R (2004). Bacterial diversity in deep Mediterranean sediments: Relationship
with the active bacterial fraction and substrate
availability.. Environ Microb.

[89] Polymenakou PN, Bertilsson S, Tselepides A, Stephanou EG (2005). Links between geographic location, environmental factors and
microbial community composition in sediments of the Eastern Mediterranean
Sea.. Microb Ecol.

[90] Amann RI, Ludwig W, Schleifer KH (1995). Phylogenetic identification and in situ detection of individual
microbial cells without cultivation.. Microbiol Rev.

[91] Li L, Kato C, Horikoshi K (1999). Bacterial diversity in deep-sea sediments from different
depths.. Biodiversity Conserv.

[92] Li L, Kato C, Horikoshi K (1999). Microbial diversity in sediments collected from the deepest
cold-seep area, the Japan Trench.. Mar Biotechnol.

[93] Lauro FM, Bartlett DH (2008). Prokaryotic lifestyles in deep Sea habitats.. Extremophiles.

[94] Hugenholtz P, Goebel BM, Pace NR (1998). Impact of culture independent studies on the emerging
phylogenetic view of bacterial diversity.. J Bacteriol.

[95] Polymenakou PN, Bertilsson S, Tselepides A, Stephanou EG (2005). Bacterial community composition in different sediments from the
Eastern Mediterranean Sea: A comparison of four 16S Ribosomal DNA clone
libraries.. Microb Ecol.

[96] Polymenakou PN, Lampadariou N, Mandalakis M, Tselepides A (2009). Phylogenetic diversity of sediment bacteria from the southern
Cretan margin, Eastern Mediterranean Sea.. Syst Appl Microbiol.

[97] Danovaro R, Corinaldesi C, Luna GM, Magagnini M, Manini E (2009). Prokaryote diversity and viral production in deep-sea sediments
and seamounts.. Deep Sea Res II.

[98] Bowman JP, McCuaig RD (2003). Biodiversity, community structural shifts, and biogeography of
prokaryotes within Antarctic continental shelf sediment.. Appl Environ Microbiol.

[99] Yakimov MM, La Cono V, Denaro R (2009). A first insight into the occurrence and expression of functional
amoA and accA genes of autotrophic and ammonia-oxidizing bathypelagic
Crenarchaeota of Tyrrhenian Sea.. Deep Sea Res II.

[100] Luna GM, Stumm K, Pusceddu A, Danovaro R (2009). Archaeal diversity in deep-sea sediments estimated by means of
different terminal-restriction fragment length polymorphisms (T-RFLP)
protocols.. Curr Microbiol.

[101] Urakawa H, Kita-Tsukamoto K, Ohwada K (1999). Microbial diversity in marine sediments from Sagami bay and Tokyo
bay, Japan, as determined by 16S rRNA gene analysis.. Microbiology.

[102] Amann RI (1995). Fluorescently labeled, ribosomal-RNA-targeted oligonucleotide
probes in the study of microbial ecology.. Mol Ecol.

[103] Barns SM, Takala SL, Kuske CR (1999). Wide distribution and diversity of members of the bacterial
kingdom Acidobacterium in the environment.. App Environ Microb.

[104] Zaballos M, Lopez-Lopez A, Ovreas L, Bartual SG, D'Auria G (2006). Comparison of prokaryotic diversity at offshore oceanic locations
reveals a different microbiota in the Mediterranean Sea.. FEMS Microbiol Ecol.

[105] Gage JD, May RM (1993). A dip into the deep seas.. Nature.

[106] Gray JS (1997). Marine biodiversity: Patterns, threats and conservation
needs.. Biodiversity Conserv.

[107] Fredj G, Laubier L, Moraitou-Apostolopoulou M, Kiortsis V (1985). The deep Mediterranean benthos.. Mediterranean marine ecosystems. NATO Conference Series.

[108] Tecchio S, Ramirez-Llodra E, Sardà F, Company B (2010). Biodiversity patterns of deep-sea benthic megafauna on western
and central Mediterranean basins.. Sci Mar.

[109] Janssen R (1989). Benthic molluscs from the deepwater of the Eastern Mediterranaean
Sea, collected during “METEOR” - cruise 5
(1987).. Senckenb Mar.

[110] Van Harten D (1987). Ostracodes and the early Holocene, anoxic event in the Eastern
Mediterranean: Evidence and implications.. Mar Geol.

[111] Macpherson E (2002). Large-scale species-richness gradients in the
Atlantic.. Oceanographic Proceeding of the Royal Society of London B.

[112] Abelló P, Cartes J (1992). Population characteristics of the deep-sea lobster
*Polycheles typhlops* and *Stereomastis
sculpta* (Decapoda: Polychelidae) in a bathyal mud community of
the Mediterranean Sea.. Mar Biol.

[113] Bouchet P, Taviani M (1992). The Mediterranean deep-sea fauna: Pseudopopulations of Atlantic
species?. Deep Sea Res A.

[114] Fishelson L, Galil BS (2001). Gonad structure and reproductive cycle in the deep-sea
herpaphrodite tripodfish, *Bathypterois mediterraneus*
(Chlorophthalmidae, Teleostei).. Copeia.

[115] D'Onghia G, Lloris D, Sion L, Capezzuto F, Labropoulou M (2004). Observations on the distribution, population structure and
biology of Bathypterois mediterraneus Bauchot, 1962 in three areas of the
Mediterranean Sea.. Sci Mar.

[116] D'Onghia G, Politou CY, Bozzano A, Lloris D, Rotllant G (2004). Deep-water fish assemblages in three areas of the Mediterranean
Sea.. Sci Mar.

[117] D'Onghia G, Sion L, Maiorano P, Mytilineou Ch, Dalessandro S (2006). Population biology and life strategies of *Chlorophthalmus
agassizii* Bonaparte, 1840 (Pisces: Osteichthyes) in the
Eastern-Central Mediterranean Sea.. Mar Biol.

[118] Matarrese A, D'Onghia G, Basanisi M, Mastrototaro F (1998). Spawning and recruitment of *Phycis blennoides*
(Brunnich, 1768) from the north-western Ionian Sea (middle-eastern
Mediterranean).. Italian Journal of Zoology.

[119] Company JB, Sardà F (1997). Reproductive patterns and population characteristics in five
deep-water pandalid shrimps in the Western Mediterranean along a depth
gradient (150–1100m).. Mar Ecol Progr Ser.

[120] Company JB, Cartes JE, Sardà F (2001). Biological patterns and near-bottom population characteristics of
two pasiphaeid decapod crustacean species, *Pasiphaea sivado*
and *Pasipahea multidentata*, in the Northwestern
Mediterranean Sea.. Mar Biol.

[121] Company JB, Sardà F, Puig P, Cartes J, Planques A (2003). Duration and timing of reproduction in decapod crustaceans of the
NW Mediterranean continental margins: Is there a general
pattern?. Mar Ecol Progr Ser.

[122] D'Onghia G, Basanisi M, Matarrese A, Megli F (1999). Reproductive strategy of macrourid fish: Seasonality or
not?. Mar Ecol Progr Ser.

[123] D'Onghia G, Lloris D, Politou C-Y, Sion L, Dokos J (2004). New records of deep-water teleost fish in the Balearic Sea and
Ionian Sea (Mediterranean Sea).. Sci Mar.

[124] Maiorano P, D'Onghia G, Capezzuto F, Sion L (2002). Life-history traits of *Plesionika martia*
(Decapoda: Caridea) from the Eastern-Central Mediterranean
Sea.. Mar Biol.

[125] Maiorano P, Pastore M, D'Onghia G, Latorre F (1998). Note on the population structure and reproduction of
*Polycheles typhlops* (Heller, 1862) (Decapoda:
Polychelidae) on the upper slope of the Ionian Sea.. J Nat Hist.

[126] Rotllant G, Moranta J, Massutí E, Sardà F, Morales-Nin B (2002). Reproductive biology of three gadiform fish species through the
Mediterranean deep-sea range (147–1850 m).. Sci Mar.

[127] Ramirez-Llodra E, Company JB, Camps M, Rotllant G (2007). Spatio-temporal variations in reproductive patterns and
population structure of *Pasiphaea multidentata* (Decapoda:
Caridea) in the Blanes canyon and adjacent margin, Northwestern
Mediterranean Sea.. Mar Ecol.

[128] Company JB, Maiorano A, Tselepides T, Politu CY, Plaity W (2004). Population characteristics of deep-sea decapod crustacean at four
different sites of the Mediterranean Sea.. Sci Mar.

[129] Ramirez-Llodra E, Ballesteros M, Company JB, Dantart L, Sardà S (2008). Spatio-temporal variations of biomass and abundance in bathyal
non-crustacean megafauna in the Catalan Sea (Northwestern
Mediterranean).. Mar Biol.

[130] Jones EG, Tselepides A, Bagley PM, Collins MA, Priede IG (2003). Bathymetric distribution of some benthic and benthopelagic
species attracted to baited cameras and traps in the deep Eastern
Mediterranean.. Mar Ecol Progr Ser.

[131] Galil B S, Clark PF (1993). A new genus and species of axiid (Decapoda, Thalassinidea) from
the Levantine basin of the Mediterranean.. Crustaceana.

[132] Stefanescu C, Lloris D, Rucabado J (1993). Deep-sea fish assemblages in the Catalan Sea (western
Mediterranean) below a depth of 1000 m.. Deep Sea Res I.

[133] Abelló P, Valladares F, Castellón A (1988). Analysis of the structure of decapod crustaceans assemblages off
the Catalan coast (North-West Mediterranean).. Mar Biol.

[134] Cartes JE, Sardà F (1992). Abundance and diversity of decapod crustaceans in the
deep-Catalan Sea (Western Mediterranean).. J Nat Hist.

[135] Sardà F, Cartes JE (1997). Morphological features and ecological aspects of early juvenile
specimens of the aristeid shrimp *Aristeus antennatus*
(Risso, 1816).. Marine Freshwater Research.

[136] Maynou F, Cartes JE (2000). Community structure of bathyal decapod crustaceans off south-west
Balearic Islands (western Mediterranean): Seasonality and regional patterns
in zonation.. J Mar Biol Ass UK.

[137] Pérès JM, Margalef R (1985). History of the Mediterranean biota and the colonization of the
depths.. Key Environments: Western Mediterranean.

[138] Laubier L, Emig C (1993). La faune benthique profonde de
Méditerranée.. NFR Della Croce. Symposium Mediterranean Seas.

[139] Morales-Nin B, Massutí E, Stefanescu C (1996). Distribution and biology of *Alepocephalus
rostratus* from the Mediterranean Sea.. J Fish Biol.

[140] Moranta J, Stefanescu C, Massutí E, Morales-Nin B, Lloris D (1998). Fish community structure and depth-related trends on the
continental slope of the Balearic Islands (Algerian basin, western
Mediterranean).. Mar Ecol Progr Ser.

[141] Stefanescu C, Rucabado J, Lloris D (1992). Depth-size trends in western Mediterranean demersal deep-sea
fishes.. Mar Ecol Progr Ser.

[142] Massutí E, Morales-Nin B, Stefanescu C (1995). Distribution and biology of five grenadier fish (Pisces:
Macrouridae) from the upper and middle slope of the northwestern
Mediterranean.. Deep Sea Res I.

[143] Moranta J, Palmer M, Massutí E, Stefanescu C, Morales-Nin B (2004). Body fish size tendencies within and among species in the
deep-sea of the western Mediterranean.. Sci Mar.

[144] Capezzuto F, Carlucci R, Maiorano P, Sion L, Battista B (2010). The bathyal benthopelagic fauna in the NW Ionian Sea: structure,
patterns and interactions.. Chem Ecol.

[145] Goren M, Mienis H, Galil BS (2006). Not so poor - New records for the deep sea fauna of the Levant
Sea, Eastern Mediterranean.. J Mar Biol Ass UK.

[146] Bogi C, Galil BS (2004). The bathyenthic and pelagic molluscan fauna off the Levantine
coast, Eastern Mediterranean.. Boll Malacol.

[147] Sorbe JC, Galil BS (2002). The bathyal Amphipoda of the Levantine coast, Eastern
Mediterranean.. Crustaceana.

[148] Danovaro R, Dell' Anno A, Fabiano M, Pusceddu A, Tselepides A (2001). Deep-sea ecosystem response to climate changes: The Eastern
Mediterranean case study.. Trends Ecol Evol.

[149] Basso D, Thomson J, Corselli C (2004). Indications of low macrobenthic activity in the deep sediments of
the eastern Mediterranean Sea.. Sci Mar.

[150] Levin LA, Sibuet M, Gooday AJ, Smith CR, Vanreusel A (2010). The roles of habitat heterogeneity in generating and maintaining
biodiversity on continental margins: an introduction.. Mar Ecol.

[151] Puig P, Palanques A, Guillen J, García-Ladona E (2000). Deep slope currents and suspended particle fluxes in and around
the Foix submarine canyon (NW Mediterranean).. Deep Sea Res I.

[152] Puig P, Ogsto AS, Mullenbach BL, Nittrouer CA, Sternberg RW (2003). Shelf-to-canyon sediment transport processes on the Eel
Continental Margin (Northern California).. Mar Geol.

[153] Gili JM, Bouillon J, Pagès F, Palanques A, Puig P (1999). Submarine canyons as habitats of prolific plankton populations:
Three new deep-sea Hydrodomedusae in the Western
Mediterranean.. Zool J Linn Soc.

[154] Gili JM, Pagès F, Bouillon J, Palanques A, Puig P (2000). A multidisciplinary approach to the understanding of hydromedusan
populations inhabiting Mediterranean submarine canyons.. Deep Sea Res I.

[155] Stefanescu C, Morales-Nin B, Massutí E (1994). Fish assemblages on the slope in the Catalan Sea (western
Mediterranean): Influence of a submarine canyon.. J Mar Biol Ass UK.

[156] Tudela S, Sardà F, Maynou F, Demestre M (2003). Influence of submarine canyons on the distribution of the
deep-water shrimp (*Aristeus antennatus*, Risso 1816) in the
northwestern Mediterranean.. Crustaceana.

[157] Vetter EW, Dayton PK (1998). Macrofaunal communities within and adjacent to a detritus-rich
submarine canyon system.. Deep Sea Res II.

[158] Zúñiga D, Flexas MM, Sánchez-Vida A, Coenjaerts J, Calafat A (2009). Particle fluxes dynamics in Blanes submarine canyon (Northwestern
Mediterranean).. Progr Oceanogr.

[159] Greene HG, Wiebe PH, Burczynski J, Youngbluth MJ (1988). Acoustical detection of high-density krill demersal layers in the
submarine canyons off georges bank.. Science.

[160] Harrold C, Light K, Lisin S (1998). Organic enrichment of submarine-canyon and continental-shelf
benthic communities by macroalgal drift imported from nearshore kelp
forests.. Limn Oceanogr.

[161] Vetter EW (1994). Hotspots of benthic production.. Nature.

[162] Bianchelli S, Gambi C, Pusceddu A, Danovaro R (2008). Trophic conditions and meiofaunal assemblages in the Bari Canyon
and the adjacent open slope (Adriatic Sea).. Chem Ecol.

[163] Margalef R (1997). Turbulence and marine life.. Sci Mar.

[164] Trincardi F, Foglini F, Verdicchio G, Asioli A, Correggiari A (2007). The impact of cascading currents on the Bari Canyon System,
SW-Adriatic Margin (Central Mediterranean).. Mar Geol.

[165] Selli R, Stanley DJW (1985). Tectonic evolution of the Tyrrhenian Sea.. Geological Evolution of the Mediterranean Basin.

[166] Acosta J, Canals M, Lòpez-Martìnez J, Munõz A, Herranz P (2002). The Balearic Promontory geomorphology (western Mediterranean):
morphostructure and active processes.. Geomorphology.

[167] Christiansen B (1989). *Acanthephyra sp*. (Crustacea: Decapoda) in the
Eastern Mediterranean Sea 9 captured by baited traps.. Senkenb Mar.

[168] Hsü KJ (1972). When the Mediterranean dried up.. Sci Am.

[169] Wezel FC, Stanley DJ, Wezel FC (1985). Structural features and basin tectonics of the Tyrrhenian
Sea.. Geological evolution of the Mediterranean Basin.

[170] Pusceddu A, Gambi C, Zeppilli D, Bianchelli S, Danovaro R (2009). Organic matter composition, meiofauna and nematode biodiversity
in deep-sea sediments surrounding two seamounts.. Deep Sea Res II.

[171] Hovland M (2008). Deep-water coral reefs: Unique biodiversity hot-spots.

[172] Zibrowius H (2003). The “White Coral Community”, canyon and
seamount faunas of the deep Mediterranean Sea..

[173] Freiwald A, Fossa JH, Grehan A, Koslow T, Roberts JM (2004). Cold-water coral reefs.

[174] Zibrowius H (1980). Les Scléractiniaires de la
Méditerranée et de l'Atlantique
nord-oriental.. Mémoires de l'Institut Océanographique
Monaco.

[175] Pérès JM, Picard J (1964). Nouveau manuel de bionomie benthique de la mer
Méditerranée.. Recueil des Travaux de la Station Marine d'Endoume.

[176] Schönberg CHL, Beuck L (2007). Where Topsent went wrong: Aka infesta a.k.a. Aka labyrinthica
(Demospongiae: Phloeodictyidae) and implications for other Aka
spp.. J Mar Biol Ass UK.

[177] Rosso A, Vertino A, Di Geronimo I, Sanfilippo R, Sciuto F (2010). Hard- and soft-bottom thanatofacies from the Santa Maria di Leuca
deep-water coral province, Mediterranean.. Deep Sea Res II.

[178] Vertino A, Savini A, Rosso A, Di Geronimo I, Mastrototaro F (2010). Benthic habitat characterization and distribution from two
representative sites of the deep-water SML coral mound province
(Mediterranean).. Deep Sea Res II.

[179] Mastrototaro F, D'Onghia G, Corriero G, Matarrese A, Maiorano P (2010). Biodiversity of the white coral and sponge community off Cape
Santa Maria di Leuca (Mediterranean Sea).. Deep Sea Res II.

[180] D'Onghia G, Maiorano P, Sion L, Giove A, Capezzuto F (2010). Effects of deep-water coral banks on the abundance and size
structure of the megafauna in the Mediterranean Sea.. Deep Sea Res II.

[181] Bourcier M, Zibrowius H (1973). Les «boues rouges»
déversées dans le canyon de la Cassidaigne
(région de Marseille). Observations en soucoupe plongeante SP 350
(juin 1971) et résultats de dragages.. Tethys.

[182] Zabala M, Maluquer P, Harmelin J-G (1993). Epibiotic bryozoans on deep-water scleractinian corals from the
Catalonian slope (Western Mediterranean, Spain, France).. Sci Mar.

[183] Zibrowius H, Taviani M, Freiwald A, Roberts JM (2005). Remarkable sessile fauna associated with deep coral and other
calcareous substrates in the Strait of Sicily, Mediterranean
Sea.. Cold Water Corals and Ecosystems.

[184] Schembri PJ, Dimech M, Camilleri M, Page R (2007). Living deep-water Lophelia and Madrepora corals in Maltese waters
(Strait of Sicily, Mediterranean Sea).. Cah Biol Mar.

[185] Buhl-Mortensen L, Mortensen PB (2004). Symbiosis in deep-water corals.. Symbiosis.

[186] Mortensen PB, Fosså JH (2006). Species diversity and spatial distribution of invertebrates on
deep-water Lophelia reef in Norway..

[187] Husebo A, Nottestand L, Fosså JH, Furevik DM, Jorgensen SB (2002). Distribution and abundance of fish in deep-sea coral
habitats.. Hydrobiologia.

[188] Krieger KJ, Wing B (2002). Megafauna associations with deep-water corals (*Primnoa
spp.*) in the Gulf of Alaska.. Hydrobiologia.

[189] Reed JK (2002). Deep-water Oculina coral reefs of Florida: Biology, impacts, and
management.. Hydrobiologia.

[190] Costello MJ, McCrea M, Freiwald A, Lundälv T, Jonsson L, Freiwald A, Roberts JM (2005). Role of cold-water Lophelia pertusa coral reefs as fish habitat
in the NE Atlantic.. Cold water corals and ecosystems.

[191] Ross SW, Quattrini AM (2007). The fish fauna associated with deep coral banks off the
southeastern United States.. Deep Sea Res I.

[192] D'Onghia G, Mastrototaro F, Matarrese A, Politou C-Y, Mytilineou Ch (2003). Biodiversity of the upper slope demersal community in the Eastern
Mediterranean: Preliminary comparison between two areas with and without
trawl fishing.. J Northwest Atl Fish Soc.

[193] Yakimov MM, Cappello S, Crisafi E, Tursi A, Savini A (2006). Phylogenetic survey of metabolically active microbial communities
associated with the deep-sea coral *Lophelia pertusa* from
the Apulian plateau, Central Mediterranean Sea.. Deep Sea Res I.

[194] Kellogg CA (2004). Tropical Archaea: diversity associated with the surface
microlayer of corals.. Mar Ecol Progr Ser.

[195] Dando PR, Stüben D, Varnavas SP (1999). Hydrothermalism in the Mediterranean Sea.. Progr Oceanogr.

[196] Uchupi C, Ballard A (1989). Evidence of hydrothermal activity on Marsili Seamount, Tyrrhenian
Basin.. Deep Sea Res A.

[197] Corselli C, Basso D (1996). First evidence of benthic communities based on chemosynthesis on
the Napoli mud volcano (Eastern Mediterranean).. Mar Geol.

[198] Salas C, Woodside J (2002). *Lucinoma kazani* n. sp. (Mollusca, Bivalvia):
Evidence of a living community associated with a cold seep in the Eastern
Mediterranean Sea.. Deep Sea Res I.

[199] Coleman DF, Ballard RD (2001). A highly concentrated region of cold hydrocarbon seeps in the
southeastern Mediterranean Sea.. Geo-Mar Lett.

[200] Olu-Le Roy K, Sibuet M, Fiala-Médioni A, Gofas S, Salas C (2004). Cold seep communities in the deep eastern Mediterranean Sea:
composition, symbiosis and spatial distribution on mud
volcanoes.. Deep Sea Res I.

[201] Zitter TAC, Huguen C, Woodside JM (2005). Geology of mud volcanoes in the eastern Mediterranean from
combined sidescan sonar and submersible surveys.. Deep Sea Res I.

[202] Charlou JL, Donval JP, Zitter T, Roy N, Jean-Baptiste P (2003). Evidence of methane venting and geochemistry of brines on mud
volcanoes of the eastern Mediterranean Sea.. Deep Sea Res I.

[203] Huguen C, Foucher JP, Mascle J, Ondréas H, Thouement M (2009). Menes caldera, a highly active site of brine seepage in the
Eastern Mediterranean Sea: “In situ” observations from
the NAUTINIL expedition (2003).. Mar Geol.

[204] Bayon G, Loncke L, Dupré S, Caprais JC, Ducassou E (2009). Multi-disciplinary investigation of fluid seepage on an unstable
margin: The case of the Central Nile deep sea fan.. Mar Geol.

[205] Dupré S, Woodside J, Foucher J-P, de Lange G, Mascle J (2007). Seafloor geological studies above active gas chimneys off Egypt
(Central Nile Deep Sea Fan).. Deep Sea Res I.

[206] Sturany R (1896). Zoologische Ergebnisse VII. Mollusken I (Prosobranchier und
Opisthobranchier; Scaphopoden; Lamellibranchier) gesammelt von SM Schiff
“Pola” 1890–18.. Denkschriften der Kaiserlichen Akademie der Wissenschaften,
Mathematische-Naturwissenschaftlischen Classe.

[207] Southward E, Andersen A, Hourdez S *Lamellibrachia anaximandri* n.sp., a new
vestimentiferan tubeworm from the Mediterranean (Annelida).. Zoosystema.

[208] Duperron S, de Beer D, Zbinden M, Boetius A, Schipani V (2009). Molecular characterization of bacteria associated with the
trophosome and the tube of Lamellibrachia sp., a siboglinid annelid from
cold seeps in the eastern Mediterranean.. FEMS Microb Ecol.

[209] Duperron S, Fiala-Médioni A, Caprais JC, Olu K, Sibuet M (2007). Evidence for chemoautotrophic symbiosis in a Mediterranean cold
seep clam (Bivalvia: Lucinidae): Comparative sequence analysis of bacterial
16S rRNA, APS reductase and RubisCO genes.. FEMS Microb Ecol.

[210] Duperron S, Halary S, Lorion J, Sibuet M, Gaill F (2008). Unexpected co-occurrence of six bacterial symbionts in the gills
of the cold seep mussel *Idas* sp. (Bivalvia:
Mytilidae).. Environ Microb.

[211] Sibuet M, Olu K (1998). Biogeography, biodiversity and fluid dependence of deep-sea
cold-seep communities at active and passive margins.. Deep Sea Res II.

[212] Sibuet M, Olu-Le Roy K, Wefer G, Billett D, Hebbeln D, Jorgensen B, Schlüter M, van Weering T (2002). Cold seep communities on continental margins: structure and
quantitative distribution relative to geological and fluid venting
patterns. Ocean Margin Systems, Springer, Berlin.

[213] Ritt B, Sarrazin J, Caprais JC, Noel P, Gauthier O (2010). First insights into the structure and environmental setting of
cold-seep communities in the Marmara Sea.. Deep Sea Res.

[214] Zitter TAC, Henry P, Aloisi G, Delaygue G, Çagatay MN (2008). Cold seeps along the main Marmara Fault in the Sea of Marmara
(Turkey).. Deep Sea Res I.

[215] Hsü KJ, Montadert L, Bernoulli D, Cita MB, Erickson A (1977). History of the Mediterranean salinity crisis.. Nature.

[216] van der Wielen PWJJ, Bolhuis H, Borin S, Daffonchio D, Corselli C (2005). The enigma of prokaryotic life in deep hypersaline anoxic
basins.. Science.

[217] Hallsworth JE, Yakimov MM, Golyshin PN, Gillion JLM, D'Auria G (2007). Limits of life in MgCl2-containing environments: Chaotropicity
defines the window.. Environ Microb.

[218] Daffonchio D, Borin S, Brusa T, Brusetti L, van der Wielen PWJJ (2006). Stratified prokaryote network in the oxic–anoxic
transition of a deep-sea halocline.. Nature.

[219] van der Wielen PWJJ, Heijs SK (2007). Sulfate-reducing prokaryotic communities in two deep hypersaline
anoxic basins in the Eastern Mediterranean deep sea.. Environ Microb.

[220] Yakimov MM, Lo Cono V, Denaro R, D'Auria G, Decembrini F (2007). Primary producing prokaryotic communities of brine, interface and
seawater above the halocline of deep anoxic lake L'Atalante,
Eastern Mediterranean Sea.. ISME J.

[221] Yakimov MM, Giuliano L, Cappello S, Denaro R, Golyshin PN (2007). Microbial community of a hydrothermal mud vent underneath the
deep-sea anoxic brine Lake Urania (Eastern Mediterranean).. Origins of Life and Evolution of Biospheres.

[222] Borin S, Brusetti L, Mapelli F, D'Auria G, Brusa T (2009). Sulfur cycling and methanogenesis primarily drive microbial
colonization of the highly sulfidic Urania deep hypersaline
basin.. PNAS.

[223] Danovaro R, Dell'Anno A, Pusceddu A, Gambi C, Heiner I, Kristensen RM (2010). The first metazoa living in permanently anoxic
conditions.. BMC Biology.

[224] Rex MA (1981). Community structure in the deep-sea benthos.. Annual Annu Rev Ecol Syst.

[225] Levin LA, Etter RJ, Rex MA, Gooday AJ, Smith CR (2001). Environmental influences on regional deep-sea species
diversity.. Annu Rev Ecol Syst.

[226] Rex MA, Crame JA, Stuart CT, Clarke A (2005). Large-scale biogeographic patterns in marine molluscs: A
confluence of history and productivity?. Ecology.

[227] Rex MA, Etter RJ, Morris JS, Crouse J, McClain CR (2006). Global bathymetric patterns of standing stock and body size in
the deep-sea benthos.. Mar Ecol Progr Ser.

[228] Buhring SI, Lampadariou N, Moodley L, Tselepides A, Witte U (2006). Benthic microbial and whole –community responses to
different amounts of C-13 enriched algae: in situ experiments in the deep
Cretan Sea (Eastern Mediterranean).. Limn Oceanogr.

[229] Hausmann K, Hulsmann N, Polianski I, Schade S, Weitere M (2002). Composition of benthic protozoan communities along a depth
transect in the Eastern Mediterranean Sea.. Deep Sea Res I.

[230] Gooday AJ (2003). Benthic foraminifera (Protista) as tools in deep-water
palaeoceanography: A review of environmental influences on faunal
characteristics.. Adv Mar Biol.

[231] Jorissen FJ, de Stigter HC, Widmark JGV (1995). A conceptual model explaining benthic foraminiferal
microhabitats.. Mar Micropaleont.

[232] Van der Zwaan GJ, Duijnstee IAP, den Dulk M, Ernst SR, Jannink NT (1999). Benthic foraminifers: proxies or problems? A review of
paleoecological concepts.. Earth Sci Rev.

[233] Fontanier C, Jorissen FJ, Chaillou G, Anschutz P, Gremare A (2005). Live foraminiferal faunas from a 2800 m deep lower canyon station
from the Bay of Biscay: Faunal response to focusing of refractory organic
matter.. Deep Sea Res I.

[234] Risgaard–Petersen N, Langezaal AM, Ingvardsen S, Schmid MC, Jetten MS (2006). Evidence for complete denitrification in a benthic
foraminifer.. Nature.

[235] Høgslund S, Revsbech NP, Cedhagen T, Nielsen LP, Gallardo VA (2008). Denitrification, nitrate turnover and aerobe respiration by
benthic foraminifera in the oxygen minimum zone off Chile.. J Exp Mar Biol Ecol.

[236] Vincx M, Bett BJ, Dinet A, Ferrero T, Gooday AJ, Blaxter JHS, Southward AJ (1994). Meiobenthos of the deep Northeast Atlantic.. Advances in Marine Biology vol. 30.

[237] Bianchelli S, Gambi C, Zeppilli D, Danovaro R (2009). Metazoan meiofauna in deep-sea canyons and adjacent open slopes:
A large-scale comparison with focus on the rare taxa.. Deep Sea Res I.

[238] Gage J, Tyler PA (2003). Food inputs, utilisation, carbon flow and
energetics.. Ecosystems of the world: The deep ocean.

[239] Massutí M, Gordon JDM, Moranta J, Swan SC, Stefanescu C (2004). Mediterranean and Atlantic deep-sea fish assemblages: Differences
in biomass composition and size-related structure.. Sci Mar.

[240] Lampitt RS, Billett DSM, Rice AL (1986). Biomass of the invertebrate megabenthos from 500 to 4100 m in the
northeast Atlantic Ocean.. Mar Biol.

[241] Sion L, Bozzano A, D'Onghia G, Capezzuto F, Panza M (2004). Chondrichthyes species in deep waters of the Mediterranean
Sea.. Sci Mar.

[242] Tosti L, Danovaro R, Dell'Anno A, Olivotto I, Bompadre S (2006). Vitellogenesis in the deep-sea shark *Centroscymnus
coelolepis*.. Chem Ecol.

[243] Sibuet M (1979). Distribution and diversity of Asteroids in Atlantic abyssal
basins.. Sarsia.

[244] Vetter EW, Dayton PK (1999). Organic enrichment by macrophyte detritus and abundance patterns
of megafaunal populations in submarine canyons.. Mar Ecol Progr Ser.

[245] Tyler PA, Ramirez-Llodra E, Billett DSM, Wefer G, Hebbeln D, Jorgensen BB, Schluter M, Van Weering TCE (2002). Larval and reproductive strategies on European continental
margins.. Ocean Margin Systems.

[246] Bellan-Santini D (1990). Mediterranean deep-sea Amphipoda: Composition, structure and
affinities of the fauna.. Progr Oceanogr.

[247] Ramirez-Llodra E, Brandt A, Danovaro R, Escobar E, German CR (2010). Deep, diverse and definitely different: unique attributes of the
world's largest ecosystem.. Biogeosciences Discuss.

[248] Derraik JGB (2002). The pollution of the marine environment by plastic debris: A
review.. Mar Poll Bull.

[249] Galil BS, Golik A, Türkay M (1995). Litter at the bottom of the sea: A sea bed survey in the Eastern
Mediterranean.. Mar Poll Bull.

[250] Galgani F, Jaunet S, Campillo A, Guenegan X, His E (1995). Distribution and abundance of debris on the continental shelf of
the northwestern Mediterranean Sea.. Mar Poll Bull.

[251] Galgani F, Souplet A, Cadiou Y (1996). Accumulation of debris on the deep sea floor off the French
Mediterranean coast.. Mar Ecol Progr Ser.

[252] Richter TO, de Stigter HC, Boer W, Jesús CC, van Weering TCE (2009). Dispersal of natural and anthropogenic lead through submarine
canyons in the Portuguese margin.. Deep Sea Res I.

[253] Rotllant G, Abad Holgado E, Sardà F, Ábalos M, Company JB (2006). Dioxin compounds in the deep-sea rose shrimp *Aristeus
antennatus* (Risso, 1816) throughout the Mediterranean
Sea.. Deep Sea Res I.

[254] Unger MA, Harvey E, Vadas GG, Vecchione M (2008). Persistent pollutants in nine species of deep-sea
cephalopods.. Mar Poll Bull.

[255] Béthoux JP, Durrieu de Madron X, Nyffeler F, Tailliez D (2002). Deep water in the western Mediterranean: Peculiar 1999 and 2000
characteristics, shelf formation hypothesis, variability since 1970 and
geochemical inferences.. J Mar Syst.

[256] Ivanov VV, Shapiro GI, Huthnance JM, Aleynik DL, Golovin PN (2004). Cascades of dense water around the world ocean.. Progr Oceanogr.

[257] Roether W, Klein B, Manca BB, Theocharis A, Kioroglou S (2007). Transient Eastern Mediterranean Deep waters in response to the
massive dense-water output of the Aegean Sea in the 1990s.. Progr Oceanogr.

[258] Danovaro R, Dell'Anno A, Pusceddu A (2004). Biodiversity response to climate change in a warm deep
Sea.. Ecol Lett.

[259] Levin LA, Dayton PK (2009). Ecological theory and continental margins: Where shallow meets
deep.. Trends Ecol Evol.

[260] Company JB, Puig P, Sardà F, Palanques A, Latasa M (2008). Climate control on deep-sea fisheries.. PLoS ONE.

[261] Smith CR, De Leo FC, Bernardino AF, Sweetman AK, Martinez Arbizu P (2008). Abyssal food limitation, ecosystem structure and climate
change.. Trends Ecol Evol.

[262] Smith KL, Ruhl HA, Bett BJ, Billet DSM, Lampitt RS (2009). Climate, carbon cycling, and deep-ocean
ecosystems.. PNAS.

[263] Palanques A, Marín J, Puig P, Guillén J, Company JB (2006). Evidence of sediment gravity flows induced by trawling in the
Palamós (Fonera) submarine canyon (northwestern
Mediterranean).. Deep Sea Res I.

[264] Martín J, Puig P, Palanques A, Masqué P, García-Orellana J (2008). Effect of commercial trawling on the deep sedimentation in a
Mediterranean submarine canyon.. Mar Geol.

[265] Danovaro R, Luna GM, Dell'Anno A, Pietrangeli B (2006). Comparison of two fingerprinting techniques, Terminal Restriction
Fragment Length Polymorphism and Automated Ribosomal Intergenic Spacer
Analysis, for determination of bacterial diversity in aquatic
environments.. Appl Environ Microbiol.

[266] Murray JW (1991). Ecology and palaeoecology of benthic
foraminifera.. Longman Scientific and Technical.

[267] Jannink NT (2001). Seasonality, biodiversity and microhabitats in benthic
foraminifera.. Geologica Ultraiectina 203.

[268] Rucabado J, Lloris D, Stefanescu C (1991). OTSB14: Un arte de arrastre bentónico para la pesca
profunda (por debajo de los mil metros).. Inf Tech de Sci Mar CSIC.

[269] Relini G (1998). Valutazione delle risorse demersali.. Biologia Marina Mediterranea.

[270] Bertrand JA, Gil de Sola L, Papaconstantinou C, Relini G, Souplet A (2002). The general specifications of the MEDITS surveys.. Sci Mar.

[271] D'Onghia G, Capezzuto F, Mytilineou Ch, Maiorano P, Kapiris K (2005). Comparison of the population structure and dynamics of
*Aristeus antennatus* (Risso, 1816) between exploited and
unexploited areas in the Mediterranean Sea.. Fish Res.

[272] Politou C-Y, Mytilineou Ch, D'Onghia G, Dokos J (2008). Demersal faunal assemblages in the deep waters of the Eastern
Ιonian Sea.. J Nat Hist.

[273] Mytilineou Ch, Politou C-Y, Papaconstantinou C, Kavadas S, D'Onghia G (2005). Deep-water fish fauna in the Eastern Ionian Sea.. Belgian Journal of Zoology.

[274] Politou C-Y, Maiorano P, D'Onghia G, Mytilineou Ch (2005). Deep-water decapod crustacean fauna of the Eastern Ionian
Sea.. Belgian Journal of Zoology.

[275] D'Onghia G, Maiorano P, Capezzuto F, Carlucci R, Battista D (2009). Further evidences of deep-sea recruitment of *Aristeus
antennatus* (Crustacea: Decapoda) and its role in the population
renewal on the exploited bottoms of the Mediterranean.. Fish Res.

[276] Sanders HL (1968). Marine benthic diversity: A comparative study.. Am Nat.

[277] Gray JS (2000). The measurement of marine species diversity, with an application
to the benthic fauna of the Norwegian continental shelf.. J Exp Mar Biol Ecol.

[278] Heijs SK, Laverman AM, Forney LJ, Hardoim PR, van Elsas JD (2008). Comparison of deep-sea sediment microbial communities in the
Eastern Mediterranean.. FEMS Microb Ecol.

[279] Álvarez-Pérez G, Busquets P, De Mol B, Sandoval NG, Canals M, Freiwald A, Roberts JM (2005). Deep-water coral occurrences in the Strait of
Gibraltar.. Cold water corals and ecosystems.

[280] Orejas C, Gori A, Gili JM (2008). Growth rates of live *Lophelia pertusa* and
*Madrepora oculata* from the Mediterranean Sea maintained
in aquaria.. Coral Reefs.

[281] Reyss D (1964). Observations faites en soucoupe plongéante dans deux
vallées sous-marines de la Mer Catalane: le rech du Cap et le
rech Lacaze-Duthiers. Bulletin de l'Institut
Océanographique.. Fondation Albert I, Prince de Monaco.

[282] Tunesi L, Diviacco G, Mo G, Martin Willison JH (2001). Observation by submersible on the Biocoenosis of the deep-sea
corals off Portofino Promontory (Northwestern Mediterranean
Sea).. Proceedings of the First International Symposium on Deep-Sea Corals,
Ecology Action Centre and Nova Scotia Museum, Halifax, Nova Scotia.

[283] Azouz A (1973). Les fonds chalutables de la région nord de la Tunisie.
1. Cadre physique et biocoenoses benthiques. Bull. Inst.
Océanogr. Pêche.. Salammbô.

[284] Vafidis D, Koukouras A, Voultsiadou-Koukoura E (1997). Actiniaria, Corallimorpharia, and Scleractinia (Hexacorallia,
Anthozoa) of the Aegean Sea, with a checklist of eastern Mediterranean and
Black Sea species.. Isr J Zool.

[285] Cartes JE (1997). Dynamics of the bathyal Benthic Boundary Layer in the
northwestern Mediterranean: depth and temporal variations in
macrofaunal–megafaunal communities and their possible connections
within deep-sea trophic webs.. Progr Oceanogr.

[286] Cartes JE, Sorbe JC (1999). Deep-water amphipods from the Catalan Sea slope (western
Mediterranean): Bathymetric distribution, assemblage composition and
biological characteristics.. Journal of Natural History.

