# Supplementary material for: Deep-Sea Biodiversity in the Mediterranean Sea: The Known, the Unknown, and the Unknowable
Source: PLoS One. 2010 Aug 2;5(8):e11832. doi: 10.1371/journal.pone.0011832 (PMC2914020; doi:10.1371/journal.pone.0011832)
Supplement: Table S1 — Data of prokaryotes biodiversity. Reported are: location, station, habitat, latitude (Lat), longitude (Long), depth, sampling gear (BC for box corer and MC for multicorer), method for analysis (C: cloning and F: fingerprinting), bacterial Richness (BR), archaeal Richness (AR), and references. (0.12 MB DOC) [file pone.0011832.s001.doc]

**Table S1.**

| Location | Station | Habitat | Lat | Long | Depth | Layer | Sampling | Method | BR | AR | Reference |
| --- | --- | --- | --- | --- | --- | --- | --- | --- | --- | --- | --- |
|  |  |  | N | E | m | cm | gear |  | OTU | OTU |  |
| Ionian Sea | St. 1 | slope | 39.6 | 18.7 | 670 | 0-1 | MC | F(T-RFLP) | 13 | n.a. | [1] |
| Ionian Sea | St.2 | slope | 39.5 | 18.3 | 1040 | 0-1 | MC | F(T-RFLP) | 13 | n.a. | [1] |
| Ionian Sea | St.3 | slope | 39.2 | 18.0 | 2570 | 0-1 | MC | F(T-RFLP) | 18 | n.a. | [1] |
| Southern Cretan margin | Creta 2 | slope |  |  | 617 | 0-1 | MC | F(T-RFLP) | n.a. | n.a. | [2,3] |
| Southern Cretan margin | Creta 3 | slope |  |  | 1494 | 0-1 | MC | F(T-RFLP) | n.a. | n.a. | [2,3] |
| Ionian Sea | Ionian-A | slope |  |  | 2790 | 0-1 | MC | F(T-RFLP) | n.a. | n.a. | [2,3] |
| Ionian Sea | Ionian-B | slope |  |  | 2860 | 0-1 | MC | F(T-RFLP) | n.a. | n.a. | [2,3] |
| Levantine Sea | Levan | slope |  |  | 1780 | 0-1 | MC | F(T-RFLP) | n.a. | n.a. | [2,3] |
| Southern Cretan margin | Creta 2 | slope |  |  | 617 | 0-1 | MC | F(DGGE) | n.a. | n.a. | [2,3] |
| Southern Cretan margin | Creta 3 | slope |  |  | 1494 | 0-1 | MC | F(DGGE) | n.a. | n.a. | [2,3] |
| Ionian Sea | Ionian-A | slope |  |  | 2790 | 0-1 | MC | F(DGGE) | n.a. | n.a. | [2,3] |
| South Ionian Sea | Ionian-B | slope |  |  | 2860 | 0-1 | MC | F(DGGE) | n.a. | n.a. | [2,3] |
| Levantine Sea | Levan | slope |  |  | 1780 | 0-1 | MC | F(DGGE) | n.a. | n.a. | [2,3] |
| Southern Cretan margin | Creta 1 | slope |  |  | 100 | 0-1 | MC | C | 478 | n.a. | [2,3] |
| Ionian Sea | Ionian-A | slope |  |  | 2790 | 0-1 | MC | C | 1306 | n.a. | [2,3] |
| E Mediterranean |  | basin | 35.2 | 21.5 | 3342 |  | BC | C | 80(CHAO-1), 93(ACE) | 28(CHAO-1), 34(ACE) | [4] |
| Ionian Sea |  | slope | 36.5 | 15.8 | 700 | 0-1 |  | C | 80 | 15 | [5] |
| Gulf of Lyon | M500T | canyon | 42.4 | 3.4 | 498 | 0-1 | MC | F(T-RFLP) | n.a. | 3 - 18 | [6] |
| Gulf of Lyon | TM 750 | canyon | 42.3 | 3.5 | 736 | 0-1 | MC | F(T-RFLP) | n.a. | 12 - 28 | [6] |
| Gulf of Lyon | CC1835 | canyon | 42.2 | 4.1 | 1836 | 0-1 | MC | F(T-RFLP) | n.a. | 11 - 25 | [6] |
| Gulf of Lyon | M571 | canyon | 42.4 | 4.4 | 506 | 0-1 | MC | F(T-RFLP) | n.a. | 6 - 21 | [6] |
| Tyrrhenian Sea | CIESM 7 | seamount | 39.9 | 12.8 | 3587 | 0-1 | MC | F(T-RFLP) | n.a. | 7 - 18 | [6] |
| Tyrrhenian Sea | CIESM 4 | slope | 39.5 | 13.4 | 3507 | 0-1 | MC | F(T-RFLP) | n.a. | 9 - 35 | [6] |
| C Mediterranean | Malta 26 | slope | 36.8 | 15.4 | 2325 | 0-1 | MC | F(T-RFLP) | n.a. | 11 - 22 | [6] |
| Tyrrhenian Sea | Marsili | seamount | 39.1 | 14.1 | 3430 | 0-1 | MC | F(ARISA/  T-RFLP) | 100 | 15 | [7] |
| Tyrrhenian Sea |  | slope | 39.5 | 13.4 | 3507 | 0-1 | MC | F(ARISA/  T-RFLP) | 66 | 12 | [7] |
| Tyrrhenian Sea | Palinuro | seamount | 39.9 | 12.8 | 3587 | 0-1 | MC | F(ARISA/  T-RFLP) | 118 | 20 | [7] |
| Tyrrhenian Sea |  | slope | 38.9 | 13.3 | 3463 | 0-1 | MC | F(ARISA/  T-RFLP) | 127 | 14 | [7] |
| Southern Cretan margin | #78 | canyon | 35.1 | 23.5 | 3603 | 0-1 | MC | C | 296 ± 66 (CHAO-1) | n.a. | [8] |
| Southern Cretan margin | #91 | canyon | 34.6 | 24.1 | 3589 | 0-1 | MC | C | 88 ± 13 (CHAO-1) | n.a. | [8] |
| Southern Cretan margin | #83 | canyon | 34.9 | 24.5 | 2670 | 0-1 | MC | C | 109 ± 16 (CHAO-1) | n.a. | [8] |
| Southern Cretan margin | #80 | slope | 35.0 | 24.6 | 215 | 0-1 | MC | C | 196 ± 35 (CHAO-1) | n.a. | [8] |
| Southern Cretan margin | #90 | slope | 35.0 | 23.8 | 520 | 0-1 | MC | C | 71 ± 11 (CHAO-1) | n.a. | [8] |
| Ionian Sea | St. 1 | slope | 39.6 | 18.7 | 670 | 1-2 | MC | F(T-RFLP) | 15 | n.a. | [1] |
| Ionian Sea | St. 1 | slope | 39.6 | 18.7 | 670 | 2-3 | MC |  | 12 |  | [1] |
| Ionian Sea | St. 1 | slope | 39.6 | 18.7 | 670 | 8-10 | MC |  | 6 |  | [1] |
| Ionian Sea | St. 2 | slope | 39.5 | 18.3 | 1040 | 1-2 | MC | F(T-RFLP) | 16 | n.a. | [1] |
| Ionian Sea | St. 2 | slope | 39.5 | 18.3 | 1040 | 2-3 | MC |  | 25 |  | [1] |
| Ionian Sea | St. 2 | slope | 39.5 | 18.3 | 1040 | 8-10 | MC |  | 10 |  | [1] |
| Ionian Sea | St. 3 | slope | 39.2 | 18.0 | 2570 | 1-2 | MC | F(T-RFLP) | 10 | n.a. | [1] |
| Ionian Sea | St. 3 | slope | 39.2 | 18.0 | 2570 | 2-3 | MC |  | 14 |  | [1] |
| Ionian Sea | St. 3 | slope | 39.2 | 18.0 | 2570 | 8-10 | MC |  | 16 |  | [1] |
| E Mediterranean |  | basin | 35.2 | 21.5 | 3342 |  | BC | C | 80(CHAO-1), 93(ACE) | 28(CHAO-1), 34(ACE) | [4] |
