# Supplementary material for: Deep-Sea Biodiversity in the Mediterranean Sea: The Known, the Unknown, and the Unknowable
Source: PLoS One. 2010 Aug 2;5(8):e11832. doi: 10.1371/journal.pone.0011832 (PMC2914020; doi:10.1371/journal.pone.0011832)
Supplement: Table S2 — Data of foraminiferal biodiversity. Reported are: location, sampling period, habitat, station, latitude (Lat), longitude (Long), depth, sampling gear (GC for gravity corer, G for grab, PC for piston corer, BC for box corer, MC for multicorer), type of assemblage A (D: dead; L/S: live and stained), Species Richness (SR), number of individuals (N), ES(51), Shannon index (log base 2), Simpson (1−λ), and references included in Text S2. (0.44 MB DOC) [file pone.0011832.s002.doc]

**Table S2**.

| Location | Period | Habitat | Station | Lat | Long | Depth | Sampling | A | SR | N | ES (51) | H' | 1-λ | Reference |
| --- | --- | --- | --- | --- | --- | --- | --- | --- | --- | --- | --- | --- | --- | --- |
|  |  |  |  | N | E | m |  |  |  |  |  |  |  |  |
| Tyrrhenian | 68-75 | bathyal | T74-42 | 39.2 | 15.6 | 1003 | GC | D | 87 | 2795 | 16.3 | 4.0 | 0.90 | [9] |
| Tyrrhenian | 68-75 | bathyal | CT69-72 | 40.2 | 10.5 | 1063 | GC | D | 57 | 485 | 19.9 | 4.3 | 0.92 | [9] |
| Tyrrhenian | 68-75 | bathyal | CT69-85 | 38.5 | 15.4 | 1124 | GC | D | 68 | 552 | 17.8 | 4.1 | 0.91 | [9] |
| Tyrrhenian | 68-75 | bathyal | CT69-91 | 38.8 | 15.6 | 1229 | GC | D | 52 | 467 | 17.4 | 4.0 | 0.91 | [9] |
| Tyrrhenian | 68-75 | bathyal | T72-44 | 40.8 | 11.7 | 1444 | GC | D | 44 | 533 | 14.6 | 3.6 | 0.87 | [9] |
| Tyrrhenian | 68-75 | bathyal | CT68-30 | 41.8 | 10.7 | 1713 | GC | D | 52 | 773 | 13.6 | 3.4 | 0.83 | [9] |
| Tyrrhenian | 68-75 | bathyal | T73-35 | 39.8 | 10.1 | 1744 | GC | D | 95 | 1456 | 19.4 | 4.3 | 0.92 | [9] |
| Tyrrhenian | 68-75 | abyssal | CT69-88 | 39.0 | 15.4 | 2239 | GC | D | 89 | 863 | 23.2 | 4.8 | 0.94 | [9] |
| Tyrrhenian | 68-75 | abyssal | T75-10 | 38.8 | 14.1 | 2386 | GC | D | 72 | 705 | 16.2 | 3.8 | 0.88 | [9] |
| Tyrrhenian | 68-75 | abyssal | CT69-46 | 40.0 | 11.8 | 2393 | GC | D | 17 | 201 | 6.6 | 2.1 | 0.70 | [9] |
| Tyrrhenian | 68-75 | abyssal | CT69-43 | 40.2 | 12.3 | 2397 | GC | D | 14 | 850 | 4.9 | 1.0 | 0.28 | [9] |
| Tyrrhenian | 68-75 | abyssal | T70-50 | 40.0 | 11.5 | 2860 | GC | D | 26 | 429 | 9.1 | 2.7 | 0.79 | [9] |
| Tyrrhenian | 68-75 | abyssal | CT69-96 | 39.5 | 11.1 | 2860 | GC | D | 108 | 1525 | 21.7 | 4.7 | 0.93 | [9] |
| Tyrrhenian | 68-75 | abyssal | CT69-53 | 39.7 | 12.0 | 3500 | GC | D | 34 | 190 | 13.6 | 3.1 | 0.80 | [9] |
| Tyrrhenian | 68-75 | abyssal | CT69-31 | 39.5 | 13.3 | 3547 | GC | D | 20 | 742 | 5.1 | 1.7 | 0.60 | [9] |
| Tyrrhenian | 68-75 | abyssal | CT69-94 | 40.6 | 12.8 | 3588 | GC | D | 30 | 232 | 10.1 | 2.7 | 0.77 | [9] |
| Tyrrhenian | 68-75 | abyssal | CT69-32 | 39.6 | 12.9 | 3593 | GC | D | 14 | 452 | 5.1 | 1.6 | 0.56 | [9] |
| Sicily Channel | 68-75 | bathyal | CS70-2 | 35.8 | 14.1 | 1018 | GC | D | 97 | 1282 | 21.7 | 4.6 | 0.92 | [9] |
| Sicily Channel | 68-75 | bathyal | CS73-30 | 36.2 | 12.9 | 1060 | GC | D | 75 | 1112 | 18.0 | 4.1 | 0.89 | [9] |
| Sicily Channel | 68-75 | bathyal | CS72-38 | 36.5 | 12.6 | 1293 | GC | D | 60 | 843 | 15.8 | 3.5 | 0.82 | [9] |
| Sicily Channel | 68-75 | bathyal | CS72-37 | 36.7 | 12.3 | 1304 | GC | D | 39 | 553 | 13.2 | 3.0 | 0.75 | [9] |
| Sicily Channel | 68-75 | bathyal | CS70-5 | 35.7 | 13.2 | 1486 | GC | D | 77 | 985 | 24.8 | 4.8 | 0.94 | [9] |
| Sicily Channel | 68-75 | bathyal | CS72-39 | 36.5 | 13.2 | 1709 | GC | D | 39 | 359 | 16.7 | 3.7 | 0.82 | [9] |
| Gulf of Lion | 08 | slope | F | 42.9 | 4.7 | 343 | MC | L/S | 40 | 120 | 26.2 | 4.4 | 0.94 | [10] |
| Gulf of Lion | 08 | slope | F | 42.9 | 4.7 | 350 | MC | L/S | 43 | 198 | 28.2 | 4.5 | 0.95 | [10] |
| Gulf of Lion | 08 | slope | E | 42.8 | 4.7 | 552 | MC | L/S | 48 | 232 | 19.3 | 4.4 | 0.93 | [10] |
| Gulf of Lion | 08 | slope | E | na | na | na | MC | L/S | 61 | 401 | 16.8 | 4.0 | 0.88 | [10] |
| Gulf of Lion | 08 | slope | D | 42.8 | 4.7 | 745 | MC | L/S | 34 | 129 | 21.6 | 4.0 | 0.91 | [10] |
| Gulf of Lion | 08 | slope | D | na | na | na | MC | L/S | 50 | 190 | 26.8 | 4.4 | 0.92 | [10] |
| Gulf of Lion | 08 | slope | C | 42.7 | 4.8 | 980 | MC | L/S | 40 | 137 | 26.8 | 4.5 | 0.93 | [10] |
| Gulf of Lion | 08 | slope | C | na | na | na | MC | L/S | 28 | 83 | 18.2 | 3.9 | 0.91 | [10] |
| Gulf of Lion | 08 | slope | B | 42.6 | 4.9 | 1488 | MC | L/S | 31 | 91 | 20.5 | 4.2 | 0.93 | [10] |
| Gulf of Lion | 08 | slope | B | na | na | na | MC | L/S | 18 | 51 | 14.4 | 3.4 | 0.87 | [10] |
| Gulf of Lion | 08 | slope | A | 42.5 | 5.0 | 1987 | MC | L/S | 28 | 87 | 17.5 | 3.9 | 0.91 | [10] |
| Gulf of Lion | 08 | slope | A | na | na | na | MC | L/S | 24 | 55 | 18.9 | 4.0 | 0.94 | [10] |
| Gulf of Lion | 97 | canyon | canyon | 42.5 | 3.5 | 920 | MC | L/S | 51 | 609 | 18.3 | 4.4 | 0.94 | [11] |
| Gulf of Lion | 97 | canyon | canyon | na | na | na | MC | L/S | 54 | 786 | 22.3 | 4.6 | 0.95 | [11] |
| Gulf of Lion | 97 | slope | slope | 42.4 | 3.7 | 800 | MC | L/S | 44 | 307 | 20.9 | 4.3 | 0.93 | [11] |
| Gulf of Lion | 97 | slope | slope | na | na | na | MC | L/S | 44 | 452 | 25.7 | 4.8 | 0.95 | [11] |
| Gulf of Lion | 98 | slope | MC540A | 42.5 | 3.5 | 911 | MC | L/S | 76 | na | na | na | na | [12] |
| Levantine | 96-98 | slope | S9 | na | na | 700 | BC | L/S | 72 | na | na | na | na | [13] |
| Adriatic | 62 | slope | 34 | na | na | 220 | G/PC | D | 24 | 88.1 | 16.5 | 3.5 | 0.87 | [14] |
| Adriatic | 62 | slope | 35 | na | na | 253 | G/PC | D | 21 | 95.1 | 14.7 | 3.5 | 0.88 | [14] |
| Adriatic | 62 | slope | 36 | na | na | 260 | G/PC | D | 23 | 86.7 | 14.1 | 3.5 | 0.87 | [14] |
| Adriatic | 62 | bathyal | 272 | na | na | 843 | G/PC | D | 30 | 71 | 19.5 | 4.0 | 0.90 | [14] |
| Adriatic | 62 | bathyal | 273 | na | na | 957 | G/PC | D | 28 | 86.3 | 19.9 | 4.2 | 0.93 | [14] |
| Adriatic | 62 | bathyal | 274 | na | na | 293 | G/PC | D | 26 | 71.2 | 15.1 | 3.6 | 0.87 | [14] |
| Adriatic | 62 | bathyal | 275 | na | na | 819 | G/PC | D | 38 | 83.3 | 23.5 | 4.5 | 0.95 | [14] |
| Adriatic | 62 | bathyal | 276 | na | na | 384 | G/PC | D | 24 | 92.2 | 17.6 | 3.8 | 0.90 | [14] |
| Adriatic | 62 | bathyal | 281 | na | na | 302 | G/PC | D | 18 | 86.7 | 13.6 | 3.0 | 0.81 | [14] |
| Adriatic | 62 | bathyal | 282 | na | na | 744 | G/PC | D | 14 | 75.1 | 12.4 | 3.2 | 0.86 | [14] |
| Adriatic | 62 | bathyal | 283 | na | na | 875 | G/PC | D | 14 | 87.3 | 9.6 | 2.6 | 0.75 | [14] |
| Adriatic | 62 | bathyal | 284 | na | na | 699 | G/PC | D | 19 | 81 | 15.6 | 3.4 | 0.87 | [14] |
| Adriatic | 62 | bathyal | 285 | na | na | 207 | G/PC | D | 26 | 93.7 | 19.6 | 4.2 | 0.94 | [14] |
| Adriatic | 62 | bathyal | 292 | na | na | 1152 | G/PC | D | 24 | 79.0 | 16.0 | 3.7 | 0.89 | [14] |
| Adriatic | 62 | bathyal | 292 | na | na | 1198 | G/PC | D | 29 | 80.5 | 18.4 | 4.0 | 0.92 | [14] |
| Adriatic | 62 | bathyal | 295 | na | na | 1161 | G/PC | D | 45 | 90.5 | 26.2 | 5.0 | 0.97 | [14] |
| Adriatic | 62 | bathyal | 295 | na | na | 1063 | G/PC | D | 30 | 84.8 | 20.5 | 4.2 | 0.93 | [14] |
| Adriatic | 62 | bathyal | 306 | na | na | 706 | G/PC | D | 35 | 87.6 | 22.5 | 4.5 | 0.95 | [14] |
| Adriatic | 62 | bathyal | 307 | na | na | 896 | G/PC | D | 36 | 89.1 | 21.6 | 4.3 | 0.94 | [14] |
| Adriatic | 62 | bathyal | 310 | na | na | 615 | G/PC | D | 27 | 72.3 | 19.0 | 4.3 | 0.95 | [14] |
| Adriatic | 91 | bathyal | AD 91-30 | 42.0 | 16.9 | 398 | BC | L/S | 76 | 704 | na | na | na | [15] |
| Adriatic | 91 | bathyal | AD 91-29 | 42.0 | 17.0 | 487 | BC | L/S | 70 | 742 | na | na | na | [15] |
| Adriatic | 91 | bathyal | AD 91-28 | 42.0 | 17.0 | 578 | BC | L/S | 56 | 1701 | na | na | na | [15] |
| Adriatic | 91 | bathyal | AD 91-27 | 42.0 | 17.0 | 664 | BC | L/S | 32 | 167 | na | na | na | [15] |
| Adriatic | 91 | bathyal | AD 91-26 | 42.0 | 17.1 | 794 | BC | L/S | 37 | 496 | na | na | na | [15] |
| Adriatic | 91 | bathyal | AD 91-25 | 42.0 | 17.2 | 898 | BC | L/S | 40 | 731 | na | na | na | [15] |
| Adriatic | 91 | bathyal | AD 91-01 | 41.8 | 17.9 | 1200 | BC | L/S | 16 | 93 | na | na | na | [15] |
| Gulf of Taranto | 96 | bathyal | KG10-2 | 40.1 | 17.0 | 993 | BC | L/S | 41 | na | na | na | na | [13] |
| Alboran | 07 | abyssal | V4A | 36.8 | 0.5 | 2688 | BC | L/S | 30 | 194 | 23.8 | 4.4 | 0.95 | Pancotti. unp |
| Alboran | 07 | abyssal | V4B | 36.5 | 1.0 | 2650 | BC | L/S | 20 | 64 | 17.2 | 3.7 | 0.91 | Pancotti. unp |
| Alboran | 07 | abyssal | V4C | 36.3 | 1.4 | 2500 | BC | L/S | 25 | 129 | 18.4 | 3.8 | 0.90 | Pancotti. unp |
| N African | 07 | abyssal | V3A | 38.4 | 6.9 | 2855 | BC | L/S | 19 | 180 | 15.5 | 3.6 | 0.90 | Pancotti. unp |
| N African | 07 | abyssal | V3B | 39.3 | 6.1 | 2854 | BC | L/S | 11 | 75 | 9.2 | 2.1 | 0.61 | Pancotti. unp |
| Catalan | 07 | abyssal | V3C | 40.6 | 5.3 | 2748 | BC | L/S | 18 | 73 | 13.8 | 3.4 | 0.87 | Pancotti. unp |
| Tyrrenian | 07 | abyssal | V2A | 39.5 | 13.0 | 3570 | BC | L/S | 14 | 158 | 10.9 | 2.7 | 0.78 | Pancotti. unp |
| Tyrrenian | 07 | abyssal | V2C | 40.0 | 12.9 | 3575 | BC | L/S | 7 | 32 | 6.8 | 2.2 | 0.72 | Pancotti. unp |
| Sicily Channel | 07 | abyssal | V11 | 36.7 | 18.7 | 3903 | BC | L/S | 1 | 3 | 1.0 | 0.0 | 0.00 | Pancotti. unp |
| Levantine | 07 | abyssal | V10A | 37.6 | 28.5 | 3032 | BC | L/S | 6 | 15 | 6.0 | 1.9 | 0.61 | Pancotti. unp |
| Levantine | 07 | abyssal | D7 | 36.2 | 25.1 | 1505 | BC | L/S | 12 | 35 | 11.3 | 3.1 | 0.87 | Pancotti. unp |
| Levantine | 07 | abyssal | S7 | 34.8 | 27.1 | 2079 | BC | L/S | 4 | 7 | 4.0 | 1.7 | 0.62 | Pancotti. unp |
| Levantine | 07 | abyssal | IP | 34.7 | 26.1 | 4345 | BC | L/S | 8 | 21 | 8.0 | 2.5 | 0.75 | Pancotti. unp |
| Catalan | 99 | bathyal | T82/16 | 38.5 | 1.5 | 210 | BC | L/S | 9 | 30.6 | 7.0 | 2.8 | 0.85 | [16] |
| Catalan | 99 | bathyal | T82/17 | 38.5 | 1.5 | 320 | BC | L/S | 10 | 45.5 | 8.0 | 2.6 | 0.81 | [16] |
| Catalan | 99 | bathyal | T82/18 | 38.4 | 1.4 | 555 | BC | L/S | 8 | 61.6 | 7.0 | 2.4 | 0.79 | [16] |
| Catalan | 99 | bathyal | T82/19 | 38.4 | 1.4 | 790 | BC | L/S | 9 | 64 | 7.8 | 2.7 | 0.81 | [16] |
| Catalan | 99 | bathyal | T82/20 | 38.4 | 1.4 | 900 | BC | L/S | 9 | 74.6 | 8.3 | 2.6 | 0.80 | [16] |
| Catalan | 99 | bathyal | T82/21 | 38.3 | 1.5 | 1500 | BC | L/S | 9 | 50.3 | 6.0 | 2.3 | 0.75 | [16] |
| Catalan | 99 | bathyal | T82/22 | 38.1 | 1.5 | 1960 | BC | L/S | 3 | 51 | 3.0 | 1.4 | 0.58 | [16] |
| Catalan | 99 | abyssal | T82/23 | 37.9 | 1.4 | 2400 | BC | L/S | 3 | 29.5 | 3.0 | 1.3 | 0.57 | [16] |
| Catalan | 99 | bathyal | T82/6 | 39 | 2.9 | 315 | BC | L/S | 7 | 35.6 | 6.0 | 2.2 | 0.76 | [16] |
| Catalan | 99 | bathyal | T82/7 | 38.9 | 2.8 | 660 | BC | L/S | 8 | 40 | 7.0 | 2.6 | 0.84 | [16] |
| Catalan | 99 | bathyal | T82/8 | 38.9 | 2.8 | 820 | BC | L/S | 10 | 78.5 | 8.9 | 2.4 | 0.77 | [16] |
| Catalan | 99 | bathyal | T82/9 | 38.9 | 2.8 | 1562 | BC | L/S | 10 | 24.1 | 9.0 | 2.9 | 0.89 | [16] |
| Catalan | 99 | bathyal | T82/10 | 38.8 | 2.8 | 1920 | BC | L/S | 8 | 71 | 6.4 | 2.0 | 0.66 | [16] |
| Catalan | 99 | abyssal | T82/11 | 38.7 | 2.8 | 2440 | BC | L/S | 7 | 60.2 | 3.8 | 1.3 | 0.47 | [16] |
| Catalan | 99 | abyssal | T87/71 | 38.9 | 10.6 | 2654 | BC | L/S | 4 | 34.4 | 4.0 | 1.7 | 0.68 | [16] |
| Catalan | 99 | bathyal | T87/68 | 38.7 | 10.7 | 1538 | BC | L/S | 9 | 59.1 | 8.9 | 2.9 | 0.86 | [16] |
| Catalan | 99 | bathyal | T87/67 | 38.7 | 10.8 | 1326 | BC | L/S | 9 | 63.7 | 8.8 | 2.8 | 0.85 | [16] |
| Catalan | 99 | bathyal | T87/66 | 38.7 | 10.8 | 1102 | BC | L/S | 9 | 52.6 | 9.0 | 2.9 | 0.86 | [16] |
| Catalan | 99 | bathyal | T87/65 | 38.6 | 10.8 | 904 | BC | L/S | 10 | 64.5 | 9.6 | 3.0 | 0.87 | [16] |
| Catalan | 99 | bathyal | T87/64 | 38.6 | 10.8 | 630 | BC | L/S | 9 | 56.1 | 8.7 | 2.4 | 0.75 | [16] |
| Catalan | 99 | bathyal | T87/63 | 38.3 | 11.2 | 785 | BC | L/S | 10 | 53.1 | 9.9 | 2.9 | 0.86 | [16] |
| Catalan | 99 | bathyal | T87/51 | 36.7 | 12.0 | 592 | BC | L/S | 10 | 29.2 | 10.0 | 2.8 | 0.84 | [16] |
| Catalan | 99 | bathyal | T87/50 | 36.7 | 12.1 | 820 | BC | L/S | 9 | 47.4 | 8.0 | 2.8 | 0.86 | [16] |
| Catalan | 99 | bathyal | T87/49 | 36.7 | 12.1 | 1205 | BC | L/S | 7 | 44.9 | 7.0 | 2.4 | 0.80 | [16] |
| Catalan | 99 | bathyal | T87/55 | 36.8 | 12.3 | 1256 | BC | L/S | 8 | 54 | 8.0 | 2.7 | 0.83 | [16] |
| Catalan | 99 | bathyal | T87/56 | 36.8 | 12.3 | 917 | BC | L/S | 9 | 66.5 | 8.9 | 2.8 | 0.84 | [16] |
| Catalan | 99 | bathyal | T87/57 | 36.8 | 12.3 | 512 | BC | L/S | 10 | 45.4 | 10.0 | 2.7 | 0.81 | [16] |
| Catalan | 99 | bathyal | T87/48 | 36.6 | 12.5 | 1307 | BC | L/S | 8 | 48.5 | 8.0 | 2.6 | 0.82 | [16] |
| Alboran | 99 | bathyal | T87/4 | 39.3 | 20.0 | 200 | BC | L/S | 10 | 35.7 | 7.0 | 2.7 | 0.82 | [16] |
| Alboran | 99 | bathyal | T82/42 | 36.5 | -3.0 | 810 | BC | L/S | 12 | 71.5 | 9.2 | 2.7 | 0.80 | [16] |
| Alboran | 99 | bathyal | T82/54 | 36.2 | -4.5 | 1080 | BC | L/S | 10 | 78.7 | 8.9 | 2.5 | 0.78 | [16] |
| Alboran | 99 | bathyal | T82/53 | 35.8 | -4.4 | 1400 | BC | L/S | 5 | 70 | 4.9 | 1.9 | 0.71 | [16] |
| Catalan | 99 | abyssal | T82/34 | 37.1 | -0.5 | 2520 | BC | L/S | 5 | 54.1 | 4.9 | 1.6 | 0.62 | [16] |
| Catalan | 99 | bathyal | T87/90 | 38.5 | 9.3 | 1500 | BC | L/S | 6 | 41.6 | 6.0 | 2.3 | 0.78 | [16] |
| Catalan | 99 | bathyal | T87/83 | 37.7 | 8.8 | 1301 | BC | L/S | 8 | 57.7 | 7.8 | 2.6 | 0.83 | [16] |
| Catalan | 99 | bathyal | T87/84 | 37.7 | 8.7 | 1554 | BC | L/S | 8 | 62.6 | 7.9 | 2.5 | 0.79 | [16] |
| Catalan | 99 | bathyal | T87/85 | 37.8 | 8.7 | 1872 | BC | L/S | 6 | 60.6 | 6.0 | 1.7 | 0.56 | [16] |
| Catalan | 99 | abyssal | T87/87 | 38.1 | 9.4 | 2587 | BC | L/S | 3 | 54.4 | 3.0 | 1.3 | 0.54 | [16] |
| Catalan | 99 | bathyal | T87/62 | 38.3 | 11.3 | 1040 | BC | L/S | 9 | 58.6 | 8.8 | 2.7 | 0.83 | [16] |
| Catalan | 99 | bathyal | T87/61 | 38.3 | 11.3 | 1246 | BC | L/S | 11 | 61.5 | 10.5 | 3.0 | 0.87 | [16] |
| Alboran | 99 | bathyal | T87/128 | 35.5 | -2.7 | 296 | BC | L/S | 13 | 50.2 | 10.0 | 3.4 | 0.91 | [16] |
| Catalan | 99 | bathyal | T87/96 | 39.0 | 0.4 | 397 | BC | L/S | 11 | 38.9 | 7.0 | 2.5 | 0.76 | [16] |
| Catalan | 99 | bathyal | T87/79 | 37.6 | 8.8 | 511 | BC | L/S | 10 | 46.8 | 8.0 | 2.8 | 0.84 | [16] |
| Alboran | 99 | bathyal | T82/40 | 36.7 | -3.1 | 300 | BC | L/S | 10 | 46.3 | 6.0 | 2.3 | 0.73 | [16] |
| Alboran | 99 | bathyal | T82/41 | 36.6 | -3.1 | 510 | BC | L/S | 9 | 69.2 | 7.6 | 2.5 | 0.78 | [16] |
| Alboran | 99 | bathyal | T82/45 | 36.1 | -3.9 | 1480 | BC | L/S | 12 | 74.9 | 9.2 | 2.6 | 0.74 | [16] |
| Alboran | 99 | bathyal | T82/44 | 36.2 | -3.9 | 1720 | BC | L/S | 9 | 46.8 | 8.0 | 2.7 | 0.83 | [16] |
| Alboran | 99 | bathyal | T82/49 | 36.0 | -2.9 | 210 | BC | L/S | 7 | 36.3 | 4.0 | 1.1 | 0.35 | [16] |
| Alboran | 99 | bathyal | T87/137 | 35.9 | -3.0 | 218 | BC | L/S | 9 | 37.5 | 7.0 | 2.0 | 0.65 | [16] |
| Alboran | 99 | bathyal | T82/48 | 36.0 | -2.9 | 390 | BC | L/S | 11 | 24.4 | 8.0 | 2.6 | 0.78 | [16] |
| Alboran | 99 | bathyal | T87/129 | 35.6 | -2.7 | 419 | BC | L/S | 13 | 55.4 | 11.0 | 3.3 | 0.90 | [16] |
| Alboran | 99 | bathyal | T87/130 | 35.6 | -2.8 | 509 | BC | L/S | 11 | 56.1 | 10.0 | 3.1 | 0.89 | [16] |
| Alboran | 99 | bathyal | T87/136 | 35.9 | -2.9 | 528 | BC | L/S | 12 | 45.5 | 7.0 | 2.7 | 0.81 | [16] |
| Alboran | 99 | bathyal | T87/131 | 35.7 | -2.9 | 704 | BC | L/S | 12 | 62.7 | 11.5 | 2.9 | 0.83 | [16] |
| Alboran | 99 | bathyal | T87/134 | 35.9 | -3.0 | 705 | BC | L/S | 13 | 44.6 | 12.0 | 3.2 | 0.88 | [16] |
| Alboran | 99 | bathyal | T82/47 | 36.1 | -2.8 | 710 | BC | L/S | 9 | 20.9 | 7.0 | 2.6 | 0.84 | [16] |
| Alboran | 99 | bathyal | T87/132 | 35.8 | -2.9 | 936 | BC | L/S | 13 | 63.2 | 10.4 | 2.8 | 0.81 | [16] |
| Alboran | 99 | bathyal | T87/135 | 35.9 | -3.0 | 937 | BC | L/S | 13 | 48.4 | 13.0 | 3.2 | 0.89 | [16] |
| Alboran | 99 | bathyal | T82/46 | 36.1 | -2.8 | 1080 | BC | L/S | 13 | 38 | 12.0 | 3.0 | 0.87 | [16] |
| Alboran | 99 | bathyal | T87/133 | 35.8 | -2.9 | 1100 | BC | L/S | 12 | 53.5 | 11.7 | 2.5 | 0.76 | [16] |
| Alboran | 99 | bathyal | T82/27 | 37.6 | -0.6 | 350 | BC | L/S | 9 | 31.5 | 7.0 | 2.4 | 0.80 | [16] |
| Alboran | 99 | bathyal | T82/28 | 37.6 | -0.6 | 560 | BC | L/S | 9 | 33.1 | 6.0 | 2.2 | 0.74 | [16] |
| Alboran | 99 | bathyal | T82/29 | 37.5 | -0.6 | 720 | BC | L/S | 8 | 34.4 | 5.0 | 2.1 | 0.74 | [16] |
| Alboran | 99 | bathyal | T82/30 | 37.5 | -0.6 | 1020 | BC | L/S | 10 | 52.1 | 10.0 | 2.4 | 0.71 | [16] |
| Alboran | 99 | bathyal | T82/31 | 37.5 | -0.6 | 1620 | BC | L/S | 13 | 49.4 | 13.0 | 3.2 | 0.89 | [16] |
| Alboran | 99 | bathyal | T82/32 | 37.4 | -0.7 | 1820 | BC | L/S | 11 | 35.6 | 11.0 | 3.1 | 0.89 | [16] |
| Alboran | 99 | abyssal | T82/33 | 37.2 | -0.5 | 2400 | BC | L/S | 5 | 48.1 | 5.0 | 1.5 | 0.60 | [16] |
| Catalan | 99 | bathyal | T87/97 | 39.0 | 0.4 | 307 | BC | L/S | 11 | 50.8 | 9.0 | 2.8 | 0.84 | [16] |
| Catalan | 99 | bathyal | T87/95 | 39.0 | 0.4 | 499 | BC | L/S | 9 | 47.1 | 9.0 | 1.8 | 0.57 | [16] |
| Catalan | 99 | bathyal | T87/94 | 38.9 | 0.4 | 695 | BC | L/S | 11 | 57.9 | 10.4 | 2.4 | 0.76 | [16] |
| Catalan | 99 | bathyal | T87/93 | 38.9 | 0.4 | 926 | BC | L/S | 9 | 70.5 | 7.7 | 1.6 | 0.51 | [16] |
| Catalan | 99 | bathyal | T87/92 | 38.9 | 0.4 | 1110 | BC | L/S | 6 | 61.1 | 5.8 | 1.6 | 0.51 | [16] |
| Catalan | 99 | bathyal | T87/77 | 37.6 | 8.9 | 300 | BC | L/S | 8 | 31.9 | 8.0 | 2.8 | 0.87 | [16] |
| Catalan | 99 | bathyal | T87/78 | 37.6 | 8.8 | 400 | BC | L/S | 8 | 37 | 7.0 | 2.5 | 0.81 | [16] |
| Catalan | 99 | bathyal | T87/80 | 37.6 | 8.8 | 700 | BC | L/S | 9 | 52.5 | 9.0 | 2.9 | 0.86 | [16] |
| Catalan | 99 | bathyal | T87/81 | 37.6 | 8.8 | 895 | BC | L/S | 10 | 64.7 | 9.7 | 3.0 | 0.86 | [16] |
| Catalan | 99 | bathyal | T87/82 | 37.6 | 8.8 | 1097 | BC | L/S | 9 | 71.6 | 8.6 | 2.7 | 0.82 | [16] |
| Alboran | 99 | bathyal | T82/57 | 36.4 | -4.7 | 300 | BC | L/S | 9 | 44.7 | 9.0 | 2.7 | 0.85 | [16] |
| Alboran | 99 | bathyal | T82/56 | 36.4 | -4.7 | 595 | BC | L/S | 11 | 68.8 | 10.7 | 2.9 | 0.82 | [16] |
| Alboran | 99 | bathyal | T82/55 | 36.3 | -4.6 | 800 | BC | L/S | 12 | 59.9 | 11.5 | 2.8 | 0.82 | [16] |
| Alboran | 99 | bathyal | T82/43 | 36.3 | -3.9 | 1180 | BC | L/S | 11 | 76.5 | 9.7 | 2.3 | 0.67 | [16] |
| Alboran | 99 | bathyal | T87/108 | 36.7 | 2.6 | 212 | BC | L/S | 11 | 50 | 11.0 | 3.1 | 0.88 | [16] |
| Alboran | 99 | bathyal | T87/109 | 36.8 | 2.7 | 301 | BC | L/S | 12 | 49.8 | 10.0 | 3.3 | 0.90 | [16] |
| Alboran | 99 | bathyal | T87/110 | 36.8 | 2.7 | 419 | BC | L/S | 12 | 46.8 | 11.0 | 2.9 | 0.84 | [16] |
| Alboran | 99 | bathyal | T87/111 | 36.8 | 2.6 | 508 | BC | L/S | 13 | 55.4 | 12.8 | 3.4 | 0.91 | [16] |
| Alboran | 99 | bathyal | T87/112 | 36.9 | 2.6 | 742 | BC | L/S | 12 | 44.2 | 12.0 | 3.0 | 0.83 | [16] |
| Alboran | 99 | bathyal | T87/113 | 36.9 | 2.6 | 911 | BC | L/S | 11 | 51.3 | 11.0 | 3.0 | 0.86 | [16] |
| Alboran | 99 | bathyal | T87/114 | 36.9 | 2.6 | 1100 | BC | L/S | 11 | 51.4 | 11.0 | 2.7 | 0.80 | [16] |
| Alboran | 99 | bathyal | T87/115 | 37.0 | 2.6 | 1313 | BC | L/S | 11 | 58.2 | 10.5 | 2.4 | 0.69 | [16] |
| Alboran | 99 | bathyal | T87/117 | 37.0 | 2.6 | 1489 | BC | L/S | 11 | 54.9 | 10.8 | 2.4 | 0.70 | [16] |
| Alboran | 99 | bathyal | T87/116 | 37.0 | 0.1 | 1798 | BC | L/S | 8 | 62.5 | 7.6 | 2.1 | 0.68 | [16] |
| Alboran | 99 | abyssal | T87/118 | 37.0 | 0.1 | 2000 | BC | L/S | 12 | 69.4 | 9.7 | 2.7 | 0.82 | [16] |
| Alboran | 99 | abyssal | T87/119 | 36.0 | 0.1 | 2594 | BC | L/S | 10 | 74.7 | 8.7 | 2.4 | 0.73 | [16] |
| Sicily Channel | 99 | bathyal | T87/39 | 34.5 | 0.7 | 408 | BC | L/S | 9 | 41 | 9.0 | 2.9 | 0.85 | [16] |
| Sicily Channel | 99 | bathyal | T87/43 | 35.9 | 0.5 | 1514 | BC | L/S | 7 | 57 | 6.6 | 1.7 | 0.58 | [16] |
| Sicily Channel | 99 | bathyal | T87/45 | 36.5 | 0.6 | 1716 | BC | L/S | 7 | 48.9 | 7.0 | 2.3 | 0.74 | [16] |
| Sicily Channel | 99 | bathyal | T87/38 | 34.5 | 0.7 | 567 | BC | L/S | 7 | 30.8 | 7.0 | 2.2 | 0.70 | [16] |
| Sicily Channel | 99 | bathyal | T87/37 | 34.5 | 0.7 | 707 | BC | L/S | 8 | 45.1 | 8.0 | 2.3 | 0.70 | [16] |
| Sicily Channel | 99 | bathyal | T87/36 | 34.5 | 0.7 | 916 | BC | L/S | 8 | 59.4 | 8.0 | 2.6 | 0.80 | [16] |
| Sicily Channel | 99 | bathyal | T87/35 | 34.4 | 0.7 | 1120 | BC | L/S | 4 | 39 | 4.0 | 1.9 | 0.73 | [16] |
| Sicily Channel | 99 | bathyal | T87/30 | 34.5 | 0.7 | 1400 | BC | L/S | 4 | 60 | 4.0 | 1.9 | 0.72 | [16] |
| Sicily Channel | 99 | bathyal | T87/29 | 34.6 | 0.7 | 1524 | BC | L/S | 4 | 58.2 | 4.0 | 1.6 | 0.61 | [16] |
| Sicily Channel | 99 | bathyal | T87/28B | 34.6 | 16.7 | 1798 | BC | L/S | 4 | 68.9 | 4.0 | 1.8 | 0.69 | [16] |
| Sicily Channel | 99 | bathyal | T87/27B | 34.7 | 16.7 | 1900 | BC | L/S | 4 | 96.8 | 4.0 | 1.9 | 0.71 | [16] |
| Sicily Channel | 99 | abyssal | T87/26B | 34.8 | 16.8 | 2915 | BC | L/S | 4 | 92.8 | 3.0 | 0.9 | 0.33 | [16] |
| Sicily Channel | 99 | abyssal | T87/24B | 35.0 | 0.7 | 2175 | BC | L/S | 3 | 100 | 2.0 | 1.0 | 0.51 | [16] |
| Sicily Channel | 99 | abyssal | T87/23B | 35.1 | 0.7 | 3073 | BC | L/S | 2 | 77.8 | 2.0 | 0.8 | 0.34 | [16] |
| Sicily Channel | 99 | abyssal | T87/22 | 35.2 | 0.7 | 3671 | BC | L/S | 1 | 58.8 | 1.0 | 0.0 | 0.00 | [16] |
| Sicily Channel | 99 | bathyal | T87/5 | 39.3 | 0.8 | 289 | BC | L/S | 9 | 40.8 | 9.0 | 2.4 | 0.77 | [16] |
| Sicily Channel | 99 | bathyal | T87/6 | 39.3 | 20.0 | 409 | BC | L/S | 8 | 39.9 | 8.0 | 2.5 | 0.80 | [16] |
| Sicily Channel | 99 | bathyal | T87/7 | 39.3 | 0.8 | 455 | BC | L/S | 8 | 45.4 | 7.0 | 2.6 | 0.82 | [16] |
| Sicily Channel | 99 | bathyal | T87/8 | 39.3 | 20.0 | 701 | BC | L/S | 9 | 48.6 | 6.0 | 2.6 | 0.83 | [16] |
| Sicily Channel | 99 | bathyal | T87/9 | 39.2 | 20.0 | 931 | BC | L/S | 7 | 51.4 | 7.0 | 2.4 | 0.82 | [16] |
| Sicily Channel | 99 | bathyal | T87/10 | 39.2 | 20.0 | 1091 | BC | L/S | 7 | 46.6 | 7.0 | 2.4 | 0.79 | [16] |
| Sicily Channel | 99 | bathyal | T87/11 | 39.2 | 19.9 | 1322 | BC | L/S | 6 | 59.4 | 5.7 | 2.1 | 0.75 | [16] |
| Sicily Channel | 99 | bathyal | T87/12 | 39.1 | 19.9 | 1505 | BC | L/S | 4 | 71.2 | 4.0 | 1.7 | 0.64 | [16] |
| Sicily Channel | 99 | bathyal | T87/13 | 38.9 | 19.9 | 1740 | BC | L/S | 6 | 78.1 | 5.3 | 1.7 | 0.62 | [16] |
| Sicily Channel | 99 | bathyal | T87/14 | 38.6 | 19.9 | 1999 | BC | L/S | 5 | 58.5 | 4.0 | 1.7 | 0.64 | [16] |
| Sicily Channel | 99 | abyssal | T87/15 | 38.3 | 19.9 | 2510 | BC | L/S | 2 | 78.7 | 2.0 | 0.5 | 0.18 | [16] |
| Levantine | 99 | bathyal | T83/59 | 33.0 | 22.9 | 273 | BC | L/S | 3 | 24.5 | 3.0 | 0.7 | 0.25 | [16] |
| Levantine | 99 | bathyal | T83/60 | 33.0 | 22.9 | 462 | BC | L/S | 6 | 35.5 | 6.0 | 1.8 | 0.64 | [16] |
| Levantine | 99 | bathyal | T83/61 | 33.1 | 23.0 | 642 | BC | L/S | 8 | 55.4 | 7.8 | 2.1 | 0.69 | [16] |
| Levantine | 99 | bathyal | T83/62 | 33.1 | 23.0 | 815 | BC | L/S | 6 | 48.7 | 6.0 | 2.2 | 0.78 | [16] |
| Levantine | 99 | bathyal | T83/63 | 33.1 | 23.0 | 1093 | BC | L/S | 7 | 45.9 | 7.0 | 2.0 | 0.67 | [16] |
| Levantine | 99 | bathyal | T83/64 | 33.1 | 1.0 | 1185 | BC | L/S | 4 | 46.2 | 4.0 | 1.7 | 0.66 | [16] |
| Levantine | 99 | bathyal | T83/65 | 33.2 | 1.0 | 1603 | BC | L/S | 4 | 32.4 | 4.0 | 2.0 | 0.76 | [16] |
| Levantine | 99 | bathyal | T83/66 | 33.2 | 1.0 | 1849 | BC | L/S | 3 | 37.5 | 3.0 | 1.2 | 0.51 | [16] |
| Levantine | 99 | abyssal | T83/67 | 33.2 | 1.0 | 2075 | BC | L/S | 5 | 54.3 | 5.0 | 2.1 | 0.76 | [16] |
| Levantine | 99 | abyssal | T83/68 | 33.3 | 1.0 | 2310 | BC | L/S | 2 | 40 | 2.0 | 1.0 | 0.51 | [16] |
| Levantine | 99 | bathyal | T83/16 | 31.3 | 29.7 | 200 | BC | L/S | 8 | 24.2 | 8.0 | 2.8 | 0.88 | [16] |
| Levantine | 99 | bathyal | T83/17 | 31.4 | 29.7 | 300 | BC | L/S | 8 | 35.6 | 8.0 | 2.6 | 0.84 | [16] |
| Levantine | 99 | bathyal | T83/19 | 31.4 | 29.7 | 563 | BC | L/S | 7 | 55.1 | 7.0 | 2.3 | 0.75 | [16] |
| Levantine | 99 | bathyal | T83/20 | 31.6 | 29.6 | 790 | BC | L/S | 7 | 77.7 | 6.7 | 1.9 | 0.61 | [16] |
| Levantine | 99 | bathyal | T83/23 | 31.9 | 29.4 | 1465 | BC | L/S | 6 | 58.6 | 5.9 | 1.8 | 0.59 | [16] |
| Levantine | 99 | abyssal | T83/25 | 32.2 | 29.3 | 2034 | BC | L/S | 3 | 88.9 | 3.0 | 1.3 | 0.54 | [16] |
| Levantine | 99 | abyssal | T83/26 | 32.4 | 29.2 | 2300 | BC | L/S | 3 | 77.8 | 3.0 | 1.1 | 0.45 | [16] |
| Levantine | 99 | abyssal | T83/27 | 32.8 | 29.1 | 2620 | BC | L/S | 3 | 64.8 | 3.0 | 1.1 | 0.44 | [16] |
| Levantine | 99 | abyssal | T83/28 | 32.8 | 29.0 | 3090 | BC | L/S | 1 | 75 | 1.0 | 0.0 | 0.00 | [16] |
