# Supplementary material for: Deep-Sea Biodiversity in the Mediterranean Sea: The Known, the Unknown, and the Unknowable
Source: PLoS One. 2010 Aug 2;5(8):e11832. doi: 10.1371/journal.pone.0011832 (PMC2914020; doi:10.1371/journal.pone.0011832)
Supplement: Table S3 — Data of nematodes biodiversity. Reported are: location, sampling period, habitat, station, latitude (Lat), longitude (Long), depth, sampling gear (BC for box corer and MC for multicorer), Species Richness (SR) and Genus Richness (values reported in red), ES(51), Shannon index (H′, log base 2), Margalef index (D), Pileou index (J) and references included in Text S2. Red values are referred to genus level. (0.48 MB DOC) [file pone.0011832.s003.doc]

**Table S3**.

| Location | Period | Habitat | Station | Lat | Long | Depth | Sampling | SR | ES(51) | H' | D | J’ | Reference |
| --- | --- | --- | --- | --- | --- | --- | --- | --- | --- | --- | --- | --- | --- |
|  |  |  |  | N | E | m |  |  |  |  |  |  |  |
| Catalan margin | 2005 | canyon | LD1 | 42.6 | 3.4 | 434 | BC | 36 | 25.3 | 4.6 | 7.4 | 0.897 | [17] |
| Catalan margin | 2005 | canyon | LD1 | 42.6 | 3.4 | 434 | BC | 32 | 28.3 | 4.9 | 8.3 | 0.880 | [17] |
| Catalan margin | 2005 | canyon | LD1 | 42.6 | 3.4 | 434 | BC | 40 | 22.3 | 4.3 | 6.5 | 0.930 | [17] |
| Catalan margin | 2005 | canyon | LD2 | 42.4 | 3.5 | 990 | BC | 42 | 25.5 | 4.6 | 7.9 | 0.907 | [17] |
| Catalan margin | 2005 | canyon | LD2 | 42.4 | 3.5 | 990 | BC | 37 | 27.4 | 4.8 | 8.6 | 0.870 | [17] |
| Catalan margin | 2005 | canyon | LD2 | 42.4 | 3.5 | 990 | BC | 37 | 23.6 | 4.4 | 7.3 | 0.873 | [17] |
| Catalan margin | 2005 | slope | SS1 | 42.1 | 3.6 | 398 | BC | 41 | 26.3 | 4.7 | 7.7 | 0.927 | [17] |
| Catalan margin | 2005 | slope | SS1 | 42.1 | 3.6 | 398 | BC | 32 | 29.2 | 5.1 | 8.7 | 0.859 | [17] |
| Catalan margin | 2005 | slope | SS1 | 42.1 | 3.6 | 398 | BC | 38 | 23.4 | 4.3 | 6.7 | 0.923 | [17] |
| Catalan margin | 2005 | canyon | CC1 | 42.3 | 3.6 | 960 | BC | 44 | 30.1 | 5.0 | 9.8 | 0.909 | [17] |
| Catalan margin | 2005 | canyon | CC1 | 42.3 | 3.6 | 960 | BC | 47 | 31.3 | 5.1 | 10.4 | 0.906 | [17] |
| Catalan margin | 2005 | canyon | CC1 | 42.3 | 3.6 | 960 | BC | 51 | 28.9 | 4.9 | 9.3 | 0.914 | [17] |
| Catalan margin | 2005 | slope | NS1 | 42.6 | 3.7 | 334 | BC | 45 | 30.2 | 5.1 | 9.4 | 0.921 | [17] |
| Catalan margin | 2005 | slope | NS1 | 42.6 | 3.7 | 334 | BC | 44 | 31.1 | 5.2 | 9.7 | 0.925 | [17] |
| Catalan margin | 2005 | slope | NS1 | 42.6 | 3.7 | 334 | BC | 47 | 29.2 | 5.0 | 9.2 | 0.939 | [17] |
| Catalan margin | 2005 | slope | SS2 | 42.1 | 3.8 | 985 | BC | 46 | 27.8 | 4.8 | 8.6 | 0.949 | [17] |
| Catalan margin | 2005 | slope | SS2 | 42.1 | 3.8 | 985 | BC | 38 | 31.1 | 5.2 | 9.4 | 0.872 | [17] |
| Catalan margin | 2005 | slope | SS2 | 42.1 | 3.8 | 985 | BC | 41 | 24.5 | 4.4 | 7.8 | 0.865 | [17] |
| Catalan margin | 2005 | canyon | CC2 | 42.2 | 3.8 | 1434 | BC | 32 | 24.7 | 4.5 | 7.5 | 0.881 | [17] |
| Catalan margin | 2005 | canyon | CC2 | 42.2 | 3.8 | 1434 | BC | 35 | 27.3 | 4.8 | 8.6 | 0.852 | [17] |
| Catalan margin | 2005 | canyon | CC2 | 42.2 | 3.8 | 1434 | BC | 42 | 22.2 | 4.3 | 6.4 | 0.900 | [17] |
| Catalan margin | 2005 | canyon | LD3 | 42.4 | 3.8 | 1497 | BC | 43 | 22.6 | 4.3 | 6.9 | 0.919 | [17] |
| Catalan margin | 2005 | canyon | LD3 | 42.4 | 3.8 | 1497 | BC | 15 | 29.7 | 5.0 | 9.2 | 0.946 | [17] |
| Catalan margin | 2005 | canyon | LD3 | 42.4 | 3.8 | 1497 | BC | 35 | 15.5 | 3.7 | 4.7 | 0.858 | [17] |
| Catalan margin | 2005 | slope | NS2 | 42.4 | 3.9 | 1022 | BC | 40 | 28.0 | 4.9 | 8.0 | 0.935 | [17] |
| Catalan margin | 2005 | slope | NS2 | 42.4 | 3.9 | 1022 | BC | 41 | 30.4 | 5.1 | 8.8 | 0.946 | [17] |
| Catalan margin | 2005 | slope | NS2 | 42.4 | 3.9 | 1022 | BC | 33 | 25.6 | 4.6 | 7.2 | 0.919 | [17] |
| Catalan margin | 2005 | slope | SS3 | 42.1 | 4.0 | 1887 | BC | 48 | 32.3 | 5.2 | 10.1 | 0.943 | [17] |
| Catalan margin | 2005 | slope | SS3 | 42.1 | 4.0 | 1887 | BC | 53 | 33.8 | 5.4 | 10.8 | 0.947 | [17] |
| Catalan margin | 2005 | slope | SS3 | 42.1 | 4.0 | 1887 | BC | 46 | 30.7 | 5.1 | 9.5 | 0.929 | [17] |
| Catalan margin | 2005 | canyon | CC3 | 42.2 | 4.3 | 1874 | BC | 25 | 25.3 | 4.5 | 6.8 | 0.953 | [17] |
| Catalan margin | 2005 | canyon | CC3 | 42.2 | 4.3 | 1874 | BC | 27 | 25.8 | 4.6 | 7.4 | 0.928 | [17] |
| Catalan margin | 2005 | canyon | CC3 | 42.2 | 4.3 | 1874 | BC | 35 | 24.8 | 4.3 | 6.2 | 0.905 | [17] |
| Catalan margin | 2005 | canyon | SC | 42.1 | 4.7 | 2342 | BC | 39 | 29.5 | 5.0 | 8.8 | 0.926 | [17] |
| Catalan margin | 2005 | canyon | SC | 42.1 | 4.7 | 2342 | BC | 46 | 31.0 | 5.2 | 9.5 | 0.933 | [17] |
| Catalan margin | 2005 | canyon | SC | 42.1 | 4.7 | 2342 | BC | 42 | 28.0 | 4.9 | 8.1 | 0.944 | [17] |
| W Med | 1998 | basin | St 10 | 40.6 | 5.0 | 2755 | BC | 31 | 25.7 | 4.5 | 6.4 | 0.919 | [18] |
| W Med | 1998 | basin | St 10 | 40.6 | 5.0 | 2755 | BC | 33 | 27.0 | 4.6 | 8.0 | 0.934 | [18] |
| W Med | 1998 | basin | St 10 | 40.6 | 5.0 | 2755 | BC | 19 | 19.0 | 4.0 | 4.8 | 0.904 | [18] |
| W Med | na | canyon | 3 | 43.0 | 5.5 | 245 | na | 88 | na | na | na | na | [19] |
| W Med | na | canyon | 5 | 43.0 | 5.5 | 340 | na | 59 | na | na | na | na | [19] |
| W Med | na | canyon | 6 | 43.0 | 5.5 | 360 | na | 47 | na | na | na | na | [19] |
| W Med | na | canyon | 7 | 43.0 | 5.5 | 387 | na | 68 | na | na | na | na | [19] |
| W Med | na | canyon | 8 | 43.0 | 5.5 | 430 | na | 108 | na | na | na | na | [19] |
| W Med | na | canyon | 9 | 43.0 | 5.5 | 485 | na | 61 | na | na | na | na | [19] |
| W Med | na | canyon | 10 | 43.0 | 5.5 | 525 | na | 24 | na | na | na | na | [19] |
| W Med | na | canyon | 11 | 43.0 | 5.5 | 580 | na | 31 | na | na | na | na | [19] |
| W Med | 1998 | basin | St 8 | 38.4 | 6.9 | 2850 | BC | 29 | 23.9 | 4.4 | 6.2 | 0.913 | [18] |
| W Med | 1998 | basin | St 8 | 38.4 | 6.9 | 2850 | BC | 37 | 27.0 | 4.7 | 7.4 | 0.921 | [18] |
| W Med | 1998 | basin | St 8 | 38.4 | 6.9 | 2850 | BC | 18 | 18.0 | 3.8 | 5.0 | 0.905 | [18] |
| W Med | na | slope | 6 | 42.7 | 8.6 | 1220 | BC | 101 | na | 6.0 | na | na | [20] |
| W Med | na | slope | 6 | 42.7 | 8.6 | 1220 | BC | 108 | na | 6.0 | na | na | [20] |
| W Med | na | slope | 6 | 42.7 | 8.6 | 1220 | BC | 93 | na | 6.0 | na | na | [20] |
| W Med | na | slope | 5 | 42.7 | 8.7 | 990 | BC | 136 | na | 6.3 | na | na | [20] |
| W Med | na | slope | 5 | 42.7 | 8.7 | 990 | BC | 153 | na | 6.5 | na | na | [20] |
| W Med | na | slope | 5 | 42.7 | 8.7 | 990 | BC | 118 | na | 6.1 | na | na | [20] |
| W Med | na | slope | 4 | 42.7 | 8.7 | 820 | BC | 109 | na | 5.7 | na | na | [20] |
| W Med | na | slope | 4 | 42.7 | 8.7 | 820 | BC | 118 | na | 5.9 | na | na | [20] |
| W Med | na | slope | 4 | 42.7 | 8.7 | 820 | BC | 99 | na | 5.5 | na | na | [20] |
| W Med | na | slope | 3 | 42.6 | 8.7 | 530 | BC | 107 | na | 6.0 | na | na | [20] |
| W Med | na | slope | 3 | 42.6 | 8.7 | 530 | BC | 108 | na | 6.1 | na | na | [20] |
| W Med | na | slope | 3 | 42.6 | 8.7 | 530 | BC | 106 | na | 5.9 | na | na | [20] |
| W Med | na | slope | 2 | 42.6 | 8.7 | 280 | BC | 148 | na | 6.5 | na | na | [20] |
| W Med | na | slope | 2 | 42.6 | 8.7 | 280 | BC | 159 | na | 6.5 | na | na | [20] |
| W Med | na | slope | 2 | 42.6 | 8.7 | 280 | BC | 137 | na | 6.5 | na | na | [20] |
| W Med | 1998 | basin | St 7 | 36.6 | 12.2 | 1290 | BC | 35 | 26.0 | 4.6 | 7.7 | 0.919 | [20] |
| W Med | 1998 | basin | St 7 | 36.6 | 12.2 | 1290 | BC | 36 | 28.6 | 4.9 | 8.0 | 0.939 | [18] |
| W Med | 1998 | basin | St 7 | 36.6 | 12.2 | 1290 | BC | 34 | 25.8 | 4.6 | 7.4 | 0.899 | [18] |
| C Med | 2005 | seamount | Palinuro | 39.9 | 12.8 | 3581 | BC | 16 | 16 | 3.7 | 4.9 | 0.937 | [21] |
| C Med | 2005 | seamount | Palinuro | 39.9 | 12.8 | 3581 | BC | 11 | 11 | 3.4 | 3.8 | 0.977 | [21] |
| C Med | 2005 | seamount | Palinuro | 39.9 | 12.8 | 3581 | BC | 18 | 18 | 4.1 | 5.7 | 0.988 | [21] |
| C Med | 2005 | basin | Ctr 2 | 38.9 | 13.3 | 3463 | BC | 19 | 19 | 3.4 | 5.0 | 0.798 | [21] |
| C Med | 2005 | basin | Ctr 2 | 38.9 | 13.3 | 3463 | BC | 20 | 20 | 4.1 | 5.8 | 0.943 | [21] |
| C Med | 2005 | basin | Ctr 2 | 38.9 | 13.3 | 3463 | BC | 27 | 27 | 4.4 | 6.7 | 0.925 | [21] |
| C Med | 2005 | basin | Ctr 1 | 39.5 | 13.4 | 3507 | BC | 29 | 29 | 4.7 | 7.6 | 0.959 | [21] |
| C Med | 2005 | basin | Ctr 1 | 39.5 | 13.4 | 3507 | BC | 22 | 22 | 4.2 | 6.0 | 0.936 | [21] |
| C Med | 2005 | basin | Ctr 1 | 39.5 | 13.4 | 3507 | BC | 10 | 10 | 3.2 | 3.5 | 0.958 | [21] |
| C Med | 2005 | seamount | Marsili | 39.1 | 14.1 | 3430 | BC | 12 | 12 | 3.6 | 4.4 | 1.000 | [21] |
| South Adriatic | 2006 | canyon | 6 | 41.3 | 17.1 | 341 | BC | 3 | 6.7 | 2.3 | 2.2 | 0.790 | [17] |
| South Adriatic | 2006 | canyon | 6 | 41.3 | 17.1 | 341 | BC | 10 | 10.2 | 3.3 | 3.4 | 0.974 | [17] |
| South Adriatic | 2006 | canyon | 6 | 41.3 | 17.1 | 341 | BC | 7 | 3.2 | 1.3 | 1.1 | 0.882 | [17] |
| South Adriatic | 2006 | canyon | 8 | 41.4 | 17.1 | 370 | BC | 46 | 30.8 | 5.0 | 9.4 | 0.947 | [17] |
| South Adriatic | 2006 | canyon | 8 | 41.4 | 17.1 | 370 | BC | 39 | 34.9 | 5.4 | 10.6 | 0.902 | [17] |
| South Adriatic | 2006 | canyon | 8 | 41.4 | 17.1 | 370 | BC | na | 26.7 | 4.7 | 8.1 | na | [17] |
| South Adriatic | 2006 | canyon | 5 | 41.4 | 17.1 | 446 | BC | 29 | 22.2 | 4.1 | 6.6 | 0.779 | [17] |
| South Adriatic | 2006 | canyon | 5 | 41.4 | 17.1 | 446 | BC | 34 | 24.1 | 4.4 | 7.2 | 0.878 | [17] |
| South Adriatic | 2006 | canyon | 5 | 41.4 | 17.1 | 446 | BC | 29 | 20.3 | 3.7 | 5.9 | 0.826 | [17] |
| South Adriatic | 2006 | canyon | 4 | 41.3 | 17.2 | 435 | BC | 20 | 26.0 | 4.4 | 6.8 | 0.926 | [17] |
| South Adriatic | 2006 | canyon | 4 | 41.3 | 17.2 | 435 | BC | 35 | 31.2 | 4.8 | 6.8 | 0.924 | [17] |
| South Adriatic | 2006 | canyon | 4 | 41.3 | 17.2 | 435 | BC | 29 | 20.8 | 4.1 | 6.8 | 0.939 | [17] |
| South Adriatic | 2006 | slope | 1 | 41.3 | 17.2 | 406 | BC | 34 | 27.6 | 4.7 | 7.7 | 0.920 | [17] |
| South Adriatic | 2006 | slope | 1 | 41.3 | 17.2 | 406 | BC | 29 | 22.0 | 4.2 | 6.4 | 0.866 | [17] |
| South Adriatic | 2006 | slope | 1 | 41.3 | 17.2 | 406 | BC | 30 | 27.7 | 4.5 | 7.1 | 0.924 | [17] |
| South Adriatic | 2006 | canyon | 2 | 41.3 | 17.2 | 590 | BC | 31 | 26.6 | 4.6 | 7.4 | 0.911 | [17] |
| South Adriatic | 2006 | canyon | 2 | 41.3 | 17.2 | 590 | BC | 32 | 28.2 | 4.7 | 8.0 | 0.904 | [17] |
| South Adriatic | 2006 | canyon | 2 | 41.3 | 17.2 | 590 | BC | 37 | 25.1 | 4.5 | 6.9 | 0.916 | [17] |
| South Adriatic | 2006 | canyon | 3 | 41.3 | 17.2 | 593 | BC | 24 | 26.2 | 4.6 | 7.2 | 0.918 | [17] |
| South Adriatic | 2006 | canyon | 3 | 41.3 | 17.2 | 593 | BC | 37 | 28.0 | 4.9 | 8.4 | 0.917 | [17] |
| South Adriatic | 2006 | canyon | 3 | 41.3 | 17.2 | 593 | BC | 35 | 24.3 | 4.3 | 6.0 | 0.920 | [17] |
| South Adriatic | 2006 | canyon | 10 | 41.3 | 17.2 | 618 | BC | 38 | 22.5 | 4.2 | 6.5 | 0.890 | [17] |
| South Adriatic | 2006 | canyon | 10 | 41.3 | 17.2 | 618 | BC | 26 | 26.6 | 4.6 | 8.0 | 0.848 | [17] |
| South Adriatic | 2006 | canyon | 10 | 41.3 | 17.2 | 618 | BC | 25 | 18.5 | 3.8 | 5.1 | 0.825 | [17] |
| South Adriatic | 2006 | canyon | 9 | 41.3 | 17.3 | 721 | BC | 39 | 31.5 | 5.0 | 9.6 | 0.946 | [17] |
| South Adriatic | 2006 | canyon | 9 | 41.3 | 17.3 | 721 | BC | 48 | 32.9 | 5.2 | 10.4 | 0.934 | [17] |
| South Adriatic | 2006 | canyon | 9 | 41.3 | 17.3 | 721 | BC | 43 | 30.0 | 4.8 | 8.7 | 0.896 | [17] |
| E Med | 1998 | basin | St 6 | 35.6 | 17.4 | 4000 | BC | 22 | 22.0 | 3.8 | 5.5 | 0.880 | [18] |
| E Med | 1998 | basin | St 6 | 35.6 | 17.4 | 4000 | BC | 24 | 22.9 | 3.9 | 5.7 | 0.918 | [18] |
| E Med | 1998 | basin | St 6 | 35.6 | 17.4 | 4000 | BC | 21 | 21.0 | 4.1 | 5.3 | 0.842 | [17] |
| South Adriatic | 2006 | slope | 11 | 41.2 | 17.6 | 908 | BC | 40 | 28.1 | 4.8 | 8.6 | 0.907 | [18] |
| South Adriatic | 2006 | slope | 11 | 41.2 | 17.6 | 908 | BC | 38 | 25.7 | 4.4 | 8.2 | 0.833 | [17] |
| South Adriatic | 2006 | slope | 11 | 41.2 | 17.6 | 908 | BC | 12 | 12.0 | 2.8 | 3.0 | 0.778 | [17] |
| E Med | 1998 | rise | CR | 38.5 | 18.0 | 2255 | BC | 36 | 27.2 | 4.8 | 7.7 | 0.931 | Danovaro. unp |
| E Med | 1998 | rise | CR | 38.5 | 18.0 | 2255 | BC | 30 | 23.3 | 4.5 | 6.4 | 0.915 | Danovaro. unp |
| E Med | 1998 | rise | CR | 38.5 | 18.0 | 2255 | BC | 35 | 26.4 | 4.7 | 7.4 | 0.926 | Danovaro. unp |
| E Med | 1998 | basin | St 5 | 35.7 | 20.1 | 3200 | BC | 29 | 21.0 | 4.3 | 5.8 | 0.897 | [18] |
| E Med | 1998 | basin | St 5 | 35.7 | 20.1 | 3200 | BC | 24 | 22.5 | 4.4 | 6.4 | 0.904 | [18] |
| E Med | 1998 | basin | St 5 | 35.7 | 20.1 | 3200 | BC | 27 | 19.5 | 4.2 | 5.0 | 0.890 | [18] |
| E Med | 1998 | basin | TH | 35.2 | 21.4 | 3225 | BC | 21 | 17.1 | 3.8 | 4.5 | 0.857 | Danovaro. unp |
| E Med | 1998 | basin | TH | 35.2 | 21.4 | 3225 | BC | 19 | 16.6 | 3.7 | 4.2 | 0.876 | Danovaro. unp |
| E Med | 1998 | basin | TH | 35.2 | 21.4 | 3225 | BC | 20 | 17.4 | 3.9 | 4.4 | 0.906 | Danovaro. unp |
| E Med | 1998 | basin | St 4 | 34.9 | 22.5 | 2950 | BC | 24 | 20.4 | 4.1 | 5.0 | 0.911 | [18] |
| E Med | 1998 | basin | St 4 | 34.9 | 22.5 | 2950 | BC | 24 | 21.0 | 4.2 | 5.5 | 0.931 | [18] |
| E Med | 1998 | basin | St 4 | 34.9 | 22.5 | 2950 | BC | 22 | 19.4 | 4.0 | 4.5 | 0.891 | [18] |
| E Med | 1989 | basin | A7 | 35.9 | 22.9 | 550 | BC | 11 | 11.0 | 3.3 | 2.4 | 0.925 | Danovaro. unp |
| E Med | 1989 | basin | A9 | 36.0 | 23.2 | 204 | BC | 37 | 37.0 | 5.0 | 9.3 | 0.968 | Danovaro. unp |
| E Med | 1989 | basin | A12 | 36.0 | 23.3 | 636 | BC | 25 | 21.9 | 4.5 | 4.5 | 0.964 | Danovaro. unp |
| E Med | 1989 | basin | A13 | 36.0 | 23.3 | 892 | BC | 30 | 23.7 | 4.7 | 5.4 | 0.939 | Danovaro. unp |
| E E Med | 1989 | basin | A14 | 36.0 | 23.4 | 1215 | BC | 17 | 16.4 | 3.9 | 3.6 | 0.965 | Danovaro. unp |
| E Med | 1989 | basin | A15 | 36.1 | 23.7 | 533 | BC | 11 | 11.0 | 3.3 | 2.3 | 0.944 | Danovaro. unp |
| Aegean Sea | 1997 | basin | S 3 | 36.0 | 23.9 | 1194 | MC | 35 | na | na | na | na | [22] |
| Aegean Sea | 1998 | basin | S 3 | 36.0 | 23.9 | 1194 | MC | 38 | na | na | na | na | [22] |
| E Med | 1989 | basin | A17 | 36.0 | 24.1 | 1147 | BC | 12 | 12.0 | 3.5 | 3.0 | 0.976 | Danovaro. unp |
| E Med | 1989 | basin | A20 | 35.9 | 24.6 | 1078 | BC | 11 | 11.0 | 3.3 | 2.8 | 0.939 | Danovaro. unp |
| E Med | 1989 | basin | A21 | 35.9 | 24.8 | 1531 | BC | 11 | 11.0 | 3.4 | 2.4 | 0.981 | Danovaro. unp |
| Aegean Sea | 1997 | basin | S 2 | 36.7 | 25.1 | 1580 | MC | 36 | na | na | na | na | [22] |
| Aegean Sea | 1998 | basin | S 2 | 36.7 | 25.1 | 1580 | MC | 34 | na | na | na | na | [22] |
| E Med | 1989 | basin | A22 | 35.9 | 25.1 | 1840 | BC | 15 | 15.0 | 3.8 | 3.8 | 0.982 | Danovaro. unp |
| Aegean Sea | 1997 | basin | N 8 | 40.4 | 25.2 | 340 | MC | 39 | na | na | na | na | [22] |
| Aegean Sea | 1998 | basin | N 8 | 40.4 | 25.2 | 340 | MC | 28 | na | na | na | na | [22] |
| Cretan sea | 1989 | basin | D7 | 36.3 | 25.2 | 1600 | BC | 13 | 13.0 | 3.6 | 3.1 | 0.977 | [23] |
| Cretan sea | 1989 | basin | D7 | 36.3 | 25.2 | 1600 | BC | 16 | 15.9 | 3.9 | 3.6 | 0.966 | [23] |
| Cretan sea | 1989 | basin | D7 | 36.3 | 25.2 | 1600 | BC | 15 | 14.7 | 3.9 | 2.9 | 0.986 | [23] |
| Cretan sea | 1994 | basin | D7 | 36.3 | 25.2 | 1600 | BC | 44 | 27.8 | 4.8 | 9.3 | 0.883 | [23] |
| Cretan sea | 1994 | basin | D7 | 36.3 | 25.2 | 1600 | BC | 34 | 25.9 | 4.7 | 7.2 | 0.918 | [23] |
| Cretan sea | 1994 | basin | D7 | 36.3 | 25.2 | 1600 | BC | 31 | 24.1 | 4.6 | 6.5 | 0.926 | [23] |
| Cretan sea | 1995 | basin | D7 | 36.3 | 25.2 | 1600 | BC | 35 | 26.5 | 4.8 | 7.4 | 0.938 | [23] |
| Cretan sea | 1995 | basin | D7 | 36.3 | 25.2 | 1600 | BC | 31 | 24.0 | 4.6 | 6.5 | 0.929 | [23] |
| Cretan sea | 1995 | basin | D7 | 36.3 | 25.2 | 1600 | BC | 35 | 24.3 | 4.6 | 7.4 | 0.889 | [23] |
| Cretan sea | 1995 | basin | D7 | 36.3 | 25.2 | 1600 | BC | 38 | 26.1 | 4.7 | 8.0 | 0.887 | [23] |
| Cretan sea | 1995 | basin | D7 | 36.3 | 25.2 | 1600 | BC | 29 | 21.2 | 4.2 | 6.1 | 0.862 | [23] |
| Cretan sea | 1995 | basin | D7 | 36.3 | 25.2 | 1600 | BC | 33 | 24.1 | 4.5 | 6.9 | 0.887 | [23] |
| Cretan sea | 1995 | basin | D7 | 36.3 | 25.2 | 1600 | BC | 32 | 23.4 | 4.5 | 6.7 | 0.895 | [23] |
| Cretan sea | 1995 | basin | D7 | 36.3 | 25.2 | 1600 | BC | 35 | 25.1 | 4.6 | 7.4 | 0.901 | [23] |
| Cretan sea | 1995 | basin | D7 | 36.3 | 25.2 | 1600 | BC | 27 | 21.0 | 4.1 | 5.6 | 0.869 | [23] |
| Cretan sea | 1997 | basin | D7 | 36.3 | 25.2 | 1600 | BC | 22 | 18.9 | 3.7 | 5.0 | 0.839 | [23] |
| Cretan sea | 1997 | basin | D7 | 36.3 | 25.2 | 1600 | BC | 30 | 21.5 | 4.2 | 6.2 | 0.861 | [23] |
| Cretan sea | 1997 | basin | D7 | 36.3 | 25.2 | 1600 | BC | 21 | 17.0 | 3.6 | 4.5 | 0.827 | [23] |
| Cretan sea | 1998 | basin | D7 | 36.3 | 25.2 | 1600 | BC | 19 | 19.0 | 4.0 | 5.0 | 0.936 | [23] |
| Cretan sea | 1998 | basin | D7 | 36.3 | 25.2 | 1600 | BC | 12 | 11.4 | 2.7 | 2.7 | 0.766 | [23] |
| Cretan sea | 1998 | basin | D7 | 36.3 | 25.2 | 1600 | BC | 31 | 20.6 | 3.9 | 6.7 | 0.779 | [23] |
| Aegean Sea | 1997 | basin | N 9 | 40.3 | 25.2 | 675 | MC | 43 | na | na | na | na | [22] |
| Aegean Sea | 1998 | basin | N 9 | 40.3 | 25.2 | 675 | MC | 32 | na | na | na | na | [22] |
| Aegean Sea | 1997 | basin | N 1 | 40.3 | 25.2 | 1271 | MC | 34 | na | na | na | na | [22] |
| Aegean Sea | 1998 | basin | N 1 | 40.3 | 25.2 | 1271 | MC | 34 | na | na | na | na | [22] |
| Aegean Sea | 1997 | basin | S 1 | 36.1 | 25.3 | 1772 | MC | 34 | na | na | na | na | [22] |
| Aegean Sea | 1998 | basin | S 1 | 36.1 | 25.3 | 1772 | MC | 31 | na | na | na | na | [22] |
| E Med | 1998 | basin | St 3 | 33.4 | 28.3 | 3055 | BC | 25 | 22.5 | 4.2 | 4.3 | 0.933 | [18] |
| E Med | 1998 | basin | St 3 | 33.4 | 28.3 | 3055 | BC | 29 | 24.4 | 4.4 | 6.0 | 0.965 | [18] |
| E Med | 1998 | basin | St 3 | 33.4 | 28.3 | 3055 | BC | 8 | 8.0 | 2.9 | 2.6 | 0.901 | [18] |
| E Med | 1998 | basin | St 1 | 35.8 | 28.7 | 3870 | BC | 19 | 19.0 | 3.9 | 4.0 | 0.913 | [18] |
| E Med | 1998 | basin | St 1 | 35.8 | 28.7 | 3870 | BC | 20 | 20.0 | 4.0 | 5.0 | 0.930 | [18] |
| E Med | 1998 | basin | St 1 | 35.8 | 28.7 | 3870 | BC | 19 | 18.7 | 3.9 | 3.0 | 0.896 | [18] |
