# Supplementary material for: Deep-Sea Biodiversity in the Mediterranean Sea: The Known, the Unknown, and the Unknowable
Source: PLoS One. 2010 Aug 2;5(8):e11832. doi: 10.1371/journal.pone.0011832 (PMC2914020; doi:10.1371/journal.pone.0011832)
Supplement: Table S4 — Data of megafauna biodiversity. Reported are: location, sampling period, habitat, station, latitude (Lat), longitude (Long), depth, sampling gear (trawl: c for commercial or OTMS), Species Richness (SR), number of individuals (N), Margalef index (D), Pielou index (J), ES(51), Shannon index (H′), Simpson (1−λ) and references included in Text S2. (0.19 MB DOC) [file pone.0011832.s004.doc]

**Table S4.**

| Location | Period | Habitat | Station | Lat | Long | Depth | Sampling | SR | N | D | J’ | ES(51) | H' | 1-λ | References |
| --- | --- | --- | --- | --- | --- | --- | --- | --- | --- | --- | --- | --- | --- | --- | --- |
|  |  |  |  | N | E | m | trawl |  |  |  |  |  |  |  |  |
| Catalan margin (Blanes) | 03/04 | canyon head | APR03C | 41.4 | 3.2 | 600 | C | 60 | 14635 | 6.2 | 0.478 | 12.1 | 2.8 | 0.71 | [24] |
| Catalan margin (Blanes) | 03/04 | canyon head | MAY03C | 41.6 | 2.8 | 576 | C | 48 | 5173 | 5.5 | 0.705 | 17.3 | 3.9 | 0.87 | [24] |
| Catalan margin (Blanes) | 03/04 | canyon head | AUG03C | 41.6 | 2.9 | 364 | C | 61 | 5747 | 6.9 | 0.783 | 22.0 | 4.6 | 0.93 | [24] |
| Catalan margin (Blanes) | 03/04 | canyon head | SEP03C | 41.6 | 2.8 | 502 | C | 64 | 9008 | 6.9 | 0.612 | 15.7 | 3.7 | 0.83 | [24] |
| Catalan margin (Blanes) | 03/04 | canyon wall | DEC03C | 41.6 | 2.8 | 475 | C | 68 | 12341 | 7.1 | 0.667 | 17.6 | 4.1 | 0.89 | [24] |
| Catalan margin (Blanes) | 03/04 | canyon wall | APR03W | 41.5 | 3.0 | 585 | C | 79 | 16974 | 8.0 | 0.607 | 16.5 | 3.8 | 0.85 | [24] |
| Catalan margin (Blanes) | 03/04 | canyon wall | MAY03W | 41.4 | 3.0 | 603 | C | 55 | 6633 | 6.1 | 0.667 | 15.7 | 3.9 | 0.89 | [24] |
| Catalan margin (Blanes) | 03/04 | canyon wall | AUG03W | 41.5 | 3.1 | 520 | C | 57 | 10537 | 6.0 | 0.594 | 13.9 | 3.5 | 0.84 | [24] |
| Catalan margin (Blanes) | 03/04 | canyon wall | SEP03W | 41.5 | 3.0 | 567 | C | 60 | 6725 | 6.7 | 0.656 | 16.3 | 3.9 | 0.88 | [24] |
| Catalan margin (Blanes) | 03/04 | canyon wall | DEC03W | 41.5 | 3.1 | 402 | C | 61 | 11866 | 6.4 | 0.531 | 13.5 | 3.1 | 0.75 | [24] |
| Catalan margin (Blanes) | 03/04 | slope | APR03M | 41.6 | 2.8 | 585 | C | 59 | 12639 | 6.1 | 0.416 | 12.4 | 2.4 | 0.55 | [24] |
| Catalan margin (Blanes) | 03/04 | slope | MAY03M | 41.4 | 3.2 | 667 | C | 59 | 11451 | 6.2 | 0.419 | 10.6 | 2.5 | 0.63 | [24] |
| Catalan margin (Blanes) | 03/04 | slope | AUG03M | 41.4 | 3.2 | 700 | C | 41 | 5183 | 4.7 | 0.553 | 11.9 | 3.0 | 0.75 | [24] |
| Catalan margin (Blanes) | 03/04 | slope | SEP03M | 41.4 | 3.2 | 631 | C | 48 | 3106 | 5.8 | 0.619 | 14.1 | 3.5 | 0.84 | [24] |
| Catalan margin (Blanes) | 03/04 | slope | DEC03M | 41.4 | 3.2 | 512 | C | 61 | 20533 | 6.0 | 0.293 | 8.0 | 1.7 | 0.44 | [24] |
| Catalan margin (Blanes) | 03/04 | canyon head | MAR04C | 41.6 | 2.8 | 585 | C | 53 | 5424 | 6.0 | 0.532 | 12.1 | 3.0 | 0.78 | [24-27] |
| Catalan margin (Blanes) | 03/04 | slope | APR04M | 41.4 | 3.1 | 695 | C | 45 | 12123 | 4.7 | 0.310 | 7.9 | 1.7 | 0.43 | [24-27] |
| Catalan margin (Blanes) | 03/04 | slope | MAR04M | 41.4 | 3.2 | 640 | C | 42 | 2558 | 5.2 | 0.716 | 16.4 | 3.9 | 0.88 | [24-27] |
| Catalan margin (Blanes) | 03/04 | canyon wall | APR04W | 41.4 | 3.0 | 585 | C | 54 | 3221 | 6.6 | 0.743 | 18.7 | 4.3 | 0.92 | [24-27] |
| Catalan margin (Blanes) | 03/04 | canyon wall | MAR04W | 41.4 | 3.0 | 567 | C | 58 | 6891 | 6.4 | 0.543 | 13.8 | 3.2 | 0.75 | [24-27] |
| Balearic Sea | 01 | slope | W1230 | 40.5 | 1.5 | 1230 | OTMS | 22 | 7354 | 2.4 | 0.540 | 9.6 | 2.4 | 0.66 | [24-28] |
| Balearic Sea | 01 | slope | W600 | 38.4 | 1.5 | 600 | OTMS | 31 | 3723 | 3.6 | 0.719 | 14.2 | 3.6 | 0.86 | [24-28] |
| Balearic Sea | 01 | slope | W800 | 38.3 | 1.5 | 800 | OTMS | 31 | 4386 | 3.6 | 0.768 | 15.5 | 3.8 | 0.89 | [24-28] |
| Balearic Sea | 01 | slope | W1000 | 38.3 | 1.5 | 1000 | OTMS | 22 | 2451 | 2.7 | 0.759 | 13.0 | 3.4 | 0.86 | [24-28] |
| Balearic Sea | 01 | slope | W1500 | 38.2 | 1.5 | 1500 | OTMS | 18 | 2848 | 2.1 | 0.773 | 12.0 | 3.2 | 0.85 | [24-28] |
| Balearic Sea | 01 | slope | W2500 | 38.2 | 2.2 | 2500 | OTMS | 18 | 1869 | 2.3 | 0.797 | 11.9 | 3.3 | 0.87 | [24-28] |
| Balearic Sea | 01 | slope | W2800 | 38.0 | 5.3 | 2800 | OTMS | 16 | 1917 | 2.0 | 0.829 | 11.5 | 3.3 | 0.88 | [25-29] |
| Western Ionian | 01 | slope | C600 | 38.2 | 16.4 | 600 | OTMS | 29 | 3414 | 3.4 | 0.807 | 16.2 | 3.9 | 0.91 | [25-29] |
| Western Ionian | 01 | slope | C800 | 38.2 | 16.4 | 800 | OTMS | 27 | 2459 | 3.3 | 0.809 | 15.6 | 3.8 | 0.90 | [25-29] |
| Western Ionian | 01 | slope | C1000 | 38.2 | 16.3 | 1000 | OTMS | 26 | 1882 | 3.3 | 0.804 | 15.9 | 3.8 | 0.89 | [25-29] |
| Western Ionian | 01 | slope | C1500 | 38.1 | 16.4 | 1500 | OTMS | 22 | 4052 | 2.5 | 0.537 | 11.0 | 2.4 | 0.61 | [25-29] |
| Western Ionian | 01 | slope | C2000 | 37.4 | 16.4 | 2000 | OTMS | 9 | 1302 | 1.1 | 0.776 | 7.2 | 2.5 | 0.78 | [25-29] |
| Western Ionian | 01 | slope | C1500 | 38.1 | 10.4 | 1500 | OTMS | 10 | 480 | 1.5 | 0.853 | 9.4 | 2.8 | 0.81 | [25-29] |
| Western Ionian | 01 | slope | C1200 | 32.1 | 16.4 | 1200 | OTMS | 21 | 5293 | 2.3 | 0.870 | 15.2 | 3.8 | 0.91 | [25-29] |
| Western Ionian | 01 | slope | C1700 | 38.1 | 16.4 | 1700 | OTMS | 9 | 1278 | 1.1 | 0.618 | 7.5 | 2.0 | 0.58 | [25-29] |
| Eastern Ionian | 01 | slope | E600 | 36.5 | 22.0 | 600 | OTMS | 34 | 8249 | 3.7 | 0.466 | 10.2 | 2.4 | 0.61 | [25-29] |
| Eastern Ionian | 01 | slope | E800 | 36.5 | 22.6 | 800 | OTMS | 29 | 2544 | 3.6 | 0.664 | 12.9 | 3.2 | 0.82 | [25-29] |
| Eastern Ionian | 01 | slope | E1300 | 36.4 | 22.1 | 1300 | OTMS | 20 | 1000 | 2.8 | 0.869 | 15.1 | 3.8 | 0.90 | [25-29] |
| Eastern Ionian | 01 | slope | E2200 | 36.3 | 22.0 | 2200 | OTMS | 13 | 1310 | 1.7 | 0.622 | 7.7 | 2.3 | 0.71 | [25-29] |
| Eastern Ionian | 01 | slope | E2600 | 36.2 | 22.0 | 2600 | OTMS | 8 | 2056 | 0.9 | 0.515 | 5.5 | 1.5 | 0.50 | [25-29] |
| Eastern Ionian | 01 | slope | E1100 | 36.4 | 22.7 | 1100 | OTMS | 14 | 1485 | 1.8 | 0.745 | 9.7 | 2.8 | 0.81 | [25-29] |
| Eastern Ionian | 01 | slope | E1700 | 36.3 | 22.1 | 1700 | OTMS | 15 | 2485 | 1.8 | 0.620 | 7.7 | 2.4 | 0.76 | [25-29] |
| Eastern Ionian | 01 | slope | E800 | 36.5 | 22.1 | 800 | OTMS | 22 | 1667 | 2.8 | 0.771 | 13.8 | 3.4 | 0.86 | [25-29] |
| Eastern Ionian | 01 | slope | E600 | 36.5 | 22.0 | 600 | OTMS | 30 | 9318 | 3.2 | 0.566 | 11.3 | 2.8 | 0.72 | [25-29] |
| Western Ionian | 01 | abyssal | A4000 | 35.4 | 17.5 | 4000 | OTMS | 8 | 633 | 1.1 | 0.709 | 6.6 | 2.1 | 0.71 | [25-29] |
| Western Ionian | 01 | abyssal | A3300 | 36.1 | 16.3 | 3300 | OTMS | 11 | 633 | 1.6 | 0.708 | 8.3 | 2.4 | 0.75 | [25-29] |
| Barcelona margin | 91/92 | slope | RET1_UP | 41.1 | 2.0 | 465 | OTMS | 53 | 11764 | 5.5 | 0.727 | 18.5 | 4.2 | 0.90 | [30] |
| Barcelona margin | 91/92 | slope | RET2_UP | 41.1 | 2.1 | 538 | OTMS | 23 | 3291 | 2.7 | 0.687 | 13.6 | 3.1 | 0.77 | [30] |
| Barcelona margin | 91/92 | slope | RET3_UP | 41.1 | 2.1 | 464 | OTMS | 19 | 4758 | 2.1 | 0.486 | 10.4 | 2.1 | 0.51 | [30] |
| Barcelona margin | 91/92 | slope | RET4_UP | 41.1 | 2.0 | 438 | OTMS | 18 | 1002 | 2.5 | 0.876 | 15.3 | 3.7 | 0.88 | [30] |
| Barcelona margin | 91/92 | slope | RET1_MID | 41.1 | 2.1 | 750 | OTMS | 13 | 577 | 1.9 | 0.775 | 11.5 | 2.9 | 0.76 | [30] |
| Barcelona margin | 91/92 | slope | RET2_MID | 41.1 | 2.1 | 550 | OTMS | 7 | 656 | 0.9 | 0.639 | 5.8 | 1.8 | 0.63 | [30] |
| Barcelona margin | 91/92 | slope | RET3_MID | 41.1 | 2.1 | 570 | OTMS | 21 | 4197 | 2.4 | 0.470 | 10.0 | 2.1 | 0.51 | [30] |
| Barcelona margin | 91/92 | slope | RET4_MID | 41.1 | 2.1 | 600 | OTMS | 13 | 569 | 1.9 | 0.737 | 11.4 | 2.7 | 0.72 | [30] |
| Barcelona margin | 91/92 | slope | RET1_LOW | 41.0 | 2.2 | 1100 | OTMS | 13 | 2482 | 1.5 | 0.550 | 7.3 | 2.0 | 0.65 | [30] |
| Barcelona margin | 91/92 | slope | RET2_LOW | 40.6 | 2.1 | 1277 | OTMS | 6 | 175 | 1.0 | 0.819 | 6.0 | 2.1 | 0.69 | [30] |
| Barcelona margin | 91/92 | slope | RET3_LOW | 40.6 | 2.1 | 1233 | OTMS | 9 | 451 | 1.3 | 0.752 | 8.4 | 2.4 | 0.70 | [30] |
| Barcelona margin | 91/92 | slope | RET4_LOW | 41.6 | 2.1 | 1241 | OTMS | 11 | 344 | 1.7 | 0.912 | 10.7 | 3.2 | 0.86 | [30] |
