# Supplementary material for: Deep-Sea Biodiversity in the Mediterranean Sea: The Known, the Unknown, and the Unknowable
Source: PLoS One. 2010 Aug 2;5(8):e11832. doi: 10.1371/journal.pone.0011832 (PMC2914020; doi:10.1371/journal.pone.0011832)
Supplement: Table S5 — Benthic megafauna and macrofauna sampled on the Eastern Mediterranean seeps. (0.13 MB DOC) [file pone.0011832.s005.doc]

**Table S5.**

| GEOGRAPHIC AREA |  | **Olimpi and Anaximander Mud Volcanoes** | | | **Nile Fan** | | | | | |
| --- | --- | --- | --- | --- | --- | --- | --- | --- | --- | --- |
| GEOLOGICAL STRUCTURE |  | Napoli mud volcano | Amsterdam and Kazan mud volcanoes | | Brine pools (Caldera) | Pockmarks | | Mud volcanoes (Amon. Isis. Osiris) | | North Alex MV |
| DEPTH |  | 2000m | 1700-2000 m | | 2500-3000m | 1600-2100m | | 800-1100 m | | 500-700m |
| PHYLUM / CLASS / *Species* |  |  |  | |  |  | |  | |  |
| PORIFERA |  |  |  | |  |  | |  | |  |
| *Rhizaxinella pyrifera* |  | x |  | |  |  | |  | |  |
| ACTINIARIA |  | X |  | |  |  | |  | |  |
| GORGONACEA |  |  |  | |  |  | | X | |  |
| NEMERTINA |  |  | X | |  |  | |  | |  |
| NEMATODA |  | x | X | |  |  | |  | |  |
| POLYCHAETA |  | x | X | | x |  | | x | |  |
| Serpulidae |  |  | X | |  |  | |  | |  |
| Siboglinidae |  |  |  | |  |  | |  | |  |
| Obturata |  |  |  | |  |  | |  | |  |
| *Lamellibrachia* n.sp. (Andersen & Southward subm) * |  | x | x | | x | x | | x | |  |
| Siboglinidae Monilifera |  |  |  | |  |  | |  | |  |
| *Siboglinum* sp. |  |  | x | |  | x | |  | |  |
| ECHIURIDA |  |  |  | |  |  | |  | |  |
| *Bonellia viridis* |  |  | X | |  |  | |  | |  |
| MOLLUSCA |  |  |  | |  |  | |  | |  |
| BIVALVIA |  |  |  | |  |  | |  | |  |
| *Idas modiolaeformis *** |  | x | x | | x | x | |  | | x |
| Lucinidae |  |  |  | |  |  | |  | |  |
| *Myrtea amorpha***** |  | Shells | x | |  | x | |  | | x |
| *Lucinoma kazani* Salas & Woodside 2002 *** |  |  | x | |  | x | | x | | x |
| Thyasiridae |  |  |  | |  |  | |  | |  |
| *Thyasira striata ***** |  |  | x | |  | shells | |  | |  |
| Vesicomyidae |  |  |  | |  |  | |  | |  |
| *Isorropodon perplexum***** |  | shells | x | |  | shells | | x | |  |
| *Yoldiella sp.* |  |  | x | |  |  | |  | |  |
| GASTROPODS |  |  |  | |  |  | |  | |  |
| *Taranis moerchi* |  | shells | shells | |  |  | |  | |  |
| *Putseysia wiseri* |  | x | x | |  |  | |  | |  |
| *Clelandella n. sp.* |  | shells | x | |  |  | |  | |  |
| *Akritogyra conspicua* |  | x | x | |  |  | |  | |  |
| *Lurifax vitreus* |  |  | x | |  |  | |  | |  |
| *Odostomia n. sp.* |  |  | shells | |  |  | |  | |  |
| *Drilliola loprestiana* |  |  | x | |  |  | |  | |  |
| *Benthomangelia macra* |  |  | x | |  |  | |  | |  |
| *Xylodiscula sp.* |  |  | shells | |  |  | |  | |  |
| *Benthonella tenella* |  |  | shells | |  |  | |  | |  |
| *Lepetella sp.* (limpets) |  |  | x | |  |  | |  | |  |
| APLACOPHORA |  |  |  | |  |  | |  | |  |
| *Falcidens sp.* |  |  | x | |  |  | |  | |  |
| CRUSTACEA |  |  |  | |  |  | |  | |  |
| Amphipoda |  | x | x | |  |  | |  | |  |
| Decapoda |  |  |  | |  |  | |  | |  |
| *Munidopsis* sp. |  |  |  | |  |  | | x | |  |
| *Munidopsis marionis* |  |  | x | |  |  | |  | |  |
| *Munidopsis acutispina* |  |  | x | |  |  | |  | |  |
| *Chaceon mediterraneus* |  | x | x | |  |  | | x | |  |
| Tanaidacea |  |  | x | |  |  | |  | |  |
| *Paranarthrura intermedia* |  |  | x | |  |  | |  | |  |
| ECHINODERMATA |  |  |  | |  |  | |  | |  |
| Echinoidea |  | x | x | |  |  | |  | |  |
| *Echinus sp.* |  | x | x | |  |  | |  | |  |
| PISCES |  |  |  | |  |  | |  | |  |
| Zoarcidae |  |  |  | |  |  | | x | |  |
| * one sulfur-oxidizing symbiont (Sox) : (16SrRNA, functional genes,TEM, FISH, in vivo experiments) [31] | | | | | | | | | |  |
| ** 6 symbionts (sulfur-oxidizing, methanotrophic symbionts and others (16SrRNA, functional genes,TEM, FISH) [32] | | | | | | | | | | |
| *** one symbiont Sox (16SrARN, functional genes, TEM, isotopes) [33] | | | | | | |  |  |  | |
| ****one symbiont Sox (SEM+ isotopes) [34] | | | |  |  | |  |  |  | |
