# Supplementary material for: Deep-Sea Biodiversity in the Mediterranean Sea: The Known, the Unknown, and the Unknowable
Source: PLoS One. 2010 Aug 2;5(8):e11832. doi: 10.1371/journal.pone.0011832 (PMC2914020; doi:10.1371/journal.pone.0011832)
Supplement: Table S6 — Data on the extension of the sea bottom at the selected depth interval and average abundance of Foraminifera, Meiofauna (as Nematoda), Macrofauna, and Megafauna. (0.04 MB DOC) [file pone.0011832.s006.doc]

**Table S6**.

|  |  |  |  |  |  |  |
| --- | --- | --- | --- | --- | --- | --- |
| Depth interval | Area per bathymetric range |  | Foraminifera | Meiofauna  (as Nematoda) | Macrofauna | Megafauna |
|  | m^2^ |  | ind m^-2^ | ind m^-2^ | ind m^-2^ | ind m^-2^ |
| 200-1000 m | 5.36E+11 |  | 891160.4 | 361511.7 | 41.0 | 0.00645 |
| 1000-2000 m | 4.49E+11 |  | 90967.3 | 148194.4 | 97.1 | 0.0033 |
| 2000-3000 m | 7.42E+11 |  | 75830.4 | 131318.7 | 2.5 | na |
| 3000-4000 m | 2.56E+11 |  | 76377.2 | 54187.6 | 9.5 | na |
| >4000 m | 1.83E+10 |  | na | na | na | na |
|  |  |  |  |  |  |  |
