# Supplementary material for: Deep-Sea Biodiversity in the Mediterranean Sea: The Known, the Unknown, and the Unknowable
Source: PLoS One. 2010 Aug 2;5(8):e11832. doi: 10.1371/journal.pone.0011832 (PMC2914020; doi:10.1371/journal.pone.0011832)
Supplement: Table S7 — The equations of the rarefaction curves reported in Figure 6(a–e). (0.02 MB DOC) [file pone.0011832.s007.doc]

**Table S7.**

a) Bacteria

(1) Y = 15.1 Ln(X) – 26.3 (n = 16, R^2^ = 0.96, p < 0.01) for 200-1000 m.

(2) Y = 20.9 Ln(X) – 40.4 (n = 16, R^2^ = 0.93, p < 0.01) for 2000-3000 m.

(3) Y = 22.1 Ln(X) – 42.9 (n = 16, R^2^ = 0.91, p < 0.01) for 3000-4000 m.

b) Foraminifera

(1) Y = 12.6 Ln(X) – 26.4 (n = 16, R^2^ = 0.94, p < 0.01) for 200-1000 m.

(2) Y = 9.5 Ln(X) – 18.3 (n = 16, R^2^ = 0.96, p < 0.01) for 1000-2000 m.

(3) Y = 11.1 Ln(X) – 22.6 (n = 16, R^2^ = 0.96, p < 0.01) for 2000-3000 m.

(4) Y = 4.4 Ln(X) – 3.8 (n = 16, R^2^ = 0.99, p < 0.01) for 3000-4000 m.

c) Meiofauna (as Nematoda)

(1) Y = 34.9 Ln(X) – 83.6 (n = 16, R^2^ = 0.95, p < 0.01) for 200-1000 m.

(2) Y = 30.9 Ln(X) – 71.3 (n = 16, R^2^ = 0.96, p < 0.01) for 1000-2000 m.

(3) Y = 24.8 Ln(X) – 54.0 (n = 16, R^2^ = 0.97, p < 0.01) for 2000-3000 m.

(4) Y = 24.9 Ln(X) – 55.4 (n = 16, R^2^ = 0.96, p < 0.01) for 3000-4000 m.

d) Macrofauna

(1) Y = 7.5 Ln(X) – 9.8 (n = 16, R^2^ = 0.99, p < 0.01) for 200-1000 m.

(2) Y = 10.4 Ln(X) – 18.7 (n = 16, R^2^ = 0.98, p < 0.01) for 1000-2000 m.

(3) Y = 0.91 Ln(X) – 4.5 (n = 16, R^2^ = 0.65, p < 0.01) for 2000-3000 m.

(4) Y = 2.8 Ln(X) – 0.7 (n = 16, R^2^ = 0.92, p < 0.01) for 3000-4000 m.

(5) Y = 3.4 Ln(X) – 1.1 (n = 16, R^2^ = 0.93, p < 0.01) for > 4000 m.

e) Megafauna

(1) Y = 20.3 Ln(X) – 46.6 (n = 16, R^2^ = 0.95, p < 0.01) for 200-1000 m.

(2) Y = 6.3 Ln(X) – 8.4 (n = 16, R^2^ = 0.99, p < 0.01) for 1000-2000 m.
