# Supplementary material for: Deep-Sea Biodiversity in the Mediterranean Sea: The Known, the Unknown, and the Unknowable
Source: PLoS One. 2010 Aug 2;5(8):e11832. doi: 10.1371/journal.pone.0011832 (PMC2914020; doi:10.1371/journal.pone.0011832)
Supplement: Text S1 — Additional references. (0.04 MB DOC) [file pone.0011832.s008.doc]

**Text S1. Additional references**

These supplemental references are not included in the main text but are provided here to complete the information dealing with some historical and current findings on biodiversity in the deep Mediterranean Sea.

Azov Y (1986) Seasonal patterns of phytoplankton productivity and abundance in nearshore oligotrophic waters of the Levant Basin (Mediterranean). J Plankton Res 8: 41-53.

Bacescu M (1985) The effects of the geological and physiological factors on the distribution of marine plants and animals in the Mediterranean. In: Moraitou-Apostolopoulou M and Kiortsis V (editors) Mediterranean Marine Ecosystems. NATO Conference Series. New York, Plenum Press, 8: 195-212 pp.

Barnard JL (1964) Deep-sea Amphipoda (Crustacea) collected by the R/V “Vema” in the Eastern Pacific Ocean and the Caribbean and Mediterranean Seas. Bull Am Mus Nat Hist 127: 1-146 (figs. 1-33.

Ben Eliahu MN, Fiege D (1996) Serpulid tube-worms (Annelida: Polychaeta) of the Central and Eastern Mediterranean with particular attention to the Levant Basin. Senckenb Mar 28(1/3): 1-51.

Berman T, Azov Y, Schneller Y, Walline P, Townsend DW (1986) Extent, transparency, and phytoplankton distribution of the neritic waters overlying the Israeli coastal shelf. Oceanol Acta 9: 439-447.

Berman T. Townsand DW. El-Sayed SZ. Trees CC. Azov Y (1984) Optical transparency. chlorophyll and primary productivity in the Eastern Mediterranean near the Israeli coast. Oceanol Acta 7: 367-372.

Boetius A, Scheibe S, Tselepides A, Thiel H (1996). Microbial biomass and activities in deep-sea sediments of the Eastern Mediterranean: trenches and benthic hotspots. Deep Sea Res I 43: 1439-1460.

Boucher G, Lambshead PJD (1995) Ecological biodiversity of marine nematodes in samples from temperate. tropical and deep Sea regions. Conservation Biology 9: 1594-1604.

Bowman JP, McCuaig RD (2003) Biodiversity, community structural shifts and biogeography of prokaryotes within Antarctic continental shelf sediment. App Environ Microb 69: 2463-2483

Brian A (1942) I Crostacei eduli del mercato di Genova (Decapoda Natantia). Boll Pesca Piscic Idrobiol 18: 25-60.

Brunelli G, Bini G (1934) Ricerche comparative sulle pesche profonde di diversi mari Italiani. Boll Pesca Piscic Idrobiol 10(6): 733-744.

Cartes JE(1997) Dynamics of the bathyal Benthic Boundary Layer in the northwestern Mediterranean: depth and temporal variations in macrofaunal–megafaunal communities and their possible connections within deep-sea trophic webs. Progr Oceanogr 41(1): 111-139

Chardy P (1973) Les Isopodes Asellotes de la plaine abyssale Atlantique. Thesis. University of Paris.

Cimerman F Langer MR (1991) Mediterranean Foraminifera Slovenska Akademija Znanosti in Umetnosti, Ljubljana 118 pp.

Company JB, Sardà F (1998) Metabolic rates and energy content of deep-sea benthic decapod crustaceans in the western Mediterranean Sea. Deep Sea Res I 45: 1861-1880.

Company JB, Sardà F (2000) Growth parameters of deep-water decapod crustaceans in the Northwestern Mediterranean Sea: a comparative approach. Mar Biol 136: 79-90.

Corselli C, Favali P, Rosso MA, Spezie G, Taviani M, Savini A, Etiope G, Tursi A, Mastrototaro F, Remia A (2006) Aplabes consortium “The Santa Maria di Leuca Lophelia reefs of the Mediterranean sea: A research in progress”. EGU General assembly, Vienna, 2-7 April 2006.

D’Onghia G, Maiorano P, Sion L (2008) A review on the reproduction of grenadiers in the Mediterranean with new data on the gonad maturity and fecundity. In Orlov AM, Iwamoto T, editors. Grenadiers of the World Oceans: Biology, Stock Assessment and Fisheries. American Fisheries Society Symposium 63: 169-184.

Desbruyères D, Guille A, Ramos J (1972) Bionomie benthique du plateau continental de la côte Catalane Espangnole. Vie Milieu 23: 335-363.

Di Geronimo I (1974) Molluschi bentonici in sedimenti recenti batiali e abissali dello Jonio. Conchiglie 10: 133-172.

D'Onghia G, Bassanisi M, Tursi A (2000) Population structure. age and growth of macrourid fish from the upper slope of the Eastern-Central Mediterranean. J Fish Biol 56: 1217-1238.

Ege V (1934) The genus Stomias Cuv, taxonomy and biogeography. Dana Report 5: 58 p..

Giglioli EH (1881) Italian deep-sea exploration in the Mediterranean. Nature 24: 381-382.

Guille A (1970) Bionomie benthique du plateau continental de la côte catalane fransaise. II. Les communautés de la macrofaune. Vie Milieu 21: 149-280.

Jahnke R (1996) The global ocean flux of particulate organic carbon, areal distribution and magnitude. Global Biogeochem Cycles10:71-88.

Jeffreys JG (1881) On the mollusca procured during the “Lightning” and “Porcupine” expeditions. 1868-70. Part III. Proceedings Zoological Society London 25: 693-724.

Jeffreys JG (1883) Mediterranean Mollusco, No 3, and other Invertebrata. Annales Magazine natural History 5: 393-401.

Kato C, Li L, Tamaoka J, Horikoshi K (1997) Molecular analyses of the sediment of the 11000-m deep Mariana Trench. Extremophiles 1: 117-123.

Kouridaki I, Polymenakou PN, Tselepides A, Smith KL (2009) Comparative phylogenetic diversity of sediment bacteria from the deep NorthEastern Pacific Ocean and the Eastern Mediterranean Sea (submitted).

Ledoyer M (1969) Aperçu sur la faune vagile de quelques biotopes de substrat dur de Méditerranée orientale comparaison avec les mêmes biotopes en Méditerranée occidentale. Tethys 1: 281-290.

Levin L, Gage JD (1998) Relationships between oxygen, organic matter and the diversity of bathyal macrofauna. Deep Sea Res I 45:129-163

Marenzeller E von (1893) Zoologische Ergebnisse. II. Polychäten des Grundes, gesammelt 1890, 1891 und 1892. Denkschriften der Kais. Akademie der Wissenschaften, mathematisch-naturwissenschaftliche Classe, 60 [Berichte der Commission für Erforschung des östlichen Mittelmeeres, 6], 25–48. 4 pl.

Margalef R (1985) Key environments: Western Mediterranean. Pergamon Press, New York, 363 pp.

Mercader M, Lloris D, Rucabado J (2001) Tots els peixos del mar català. Diagnosi i claus d’indentificació. Institut d’Estudis Catalans (Arxius de les seccions de ciències; 128) 212 pp.

Morel A, André JM (1991) Sediment distribution and primary production in the Western Mediterranean derived and modeled from coastal zone color scanner observations. J Geophys Res B 96: 12685-12689.

Murray J, Hjort J (1912) The Depths of the Ocean. McMillan and Co (ed), London, 821 pp.

Murray JW (1991) Ecology and Palaeoecology of benthic Foraminifera. Longman Scientific and Technical, New York, 397 pp.

Parenzan P (1960) Pesci abissali e preabissali del Golfo di Taranto. Thalassia Jonica 3: 3-68.

Parenzan P (1970) Esplorazione bentonica batiale e abissale dello Jonio. Thalassia Salentina 4: 3-39.

Parker FL (1958) Eastern Mediterranean Foraminifera. Reports of the Swedish Deep-sea Expedition 8: 217-283.

Pastore M (1994) Condizioni ambientali del Mediterraneo. Rivista Marittima: 111-121.

Pérès JM (1967) The Mediterranean benthos. Oceanogr Mar Biol Annu Rev 5: 449-533.

Pérès JM (1982) Zonations: General features of organismic assemblages in pelagial and benthal. In: O Kinne (ed) Marine Ecology 1, Chichester John Wiley 1, Chichester: 9-66.

Platt HM, Warwick RM (1983) A synopsis of the free-living marine nematodes. Part I: British Enoplids. Cambridge University press. 307.

Platt HM, Warwick RM (1988) A synopsis of the free-living marine nematodes. Part II: British Chromadorids. Cambridge University press, 502.

Polymenakou P, Tselepides A, Stephanou E, Bertilsson S (2006) Carbon speciation and composition of natural microbial communities in polluted and pristine sediments of the Eastern Mediterranean Sea. Mar Poll Bull 52: 1396-1405.

Polymenakou PN, Tselepides A, Stephanou EG (2005c) Study of the mineralization effect on the distribution of lipids in sediments from the Cretan Sea: evidence for hydrocarbon degradation and starvation stress. Cont Shelf Res 25: 2196–2212.

Por FD, Dimentman C (1989). The Legacy of Tethys. An Aguatic Biogeography of the Levant. Kluwer Academic Publishers, Netherlands. 214pp.

Puig P, Palanques A (1998) Nepheloid structure and hydrographic control on the Barcelona continental margin, north-Western Mediterranean. Mar Geol 149: 39-54.

Quignard JP, Tomasini JA (2000) Mediterranean fish biodiversity. Biologia Marina Mediterranea 7 (3): 1-66.

Raulin V (1870) Description physique de l' île de Crete. Actes Societe Linnee Bordeaux 24: 353-770.

Reyss D (1971) Les canyons sous-marins de la mer Catalane, le rech du cap et le rech Lacaze-Duthiers, III. Les peuplements de macrofauna benthique. Vie Milieu 22: 529-613.

Risso A (1810) Ichthyologie de Nice ou histoire naturelle des poisons du department des Alpes Maritimes. Paris, F. Schoell. 388 pp.

Risso A (1826) Histoire naturelle des principales productions de l’Europe méridionale et particulièrment de celles des environs de Nice et des Alpes Maritimes. Paris: FG Levrault. 5 vols.

Risso A (1827) Observations sur quelques nouvelles espèces des Crustacés de la mer de Nice. Nova Acta Acad. Leop. Carol. 13: 817-822.

Roule L (1919) Poissons provenant des campagnes du yacht Princesse Alice (1891-1913) et du yacht Hirondelle II (1914). Résultats des Campagnes Scientifiques accomplies par le Prince Albert I, Monaco 52: 191 pp.

Rowe GT (1971) Benthic biomass and surface productivity. In: Costlow JD (editor) Fertility of the Sea. Gordon Gordon Breach, New York, 442-454.

Ryland JS (2000) European marine biology: past, present and future. Biologia Marina Mediterranea 7 (1): 1-27.

Sgarella F, Moncharmont-Zei M (1993) Benthic foraminifera of the > Gulf of Naples (Italy): systematic and autoecology. Bollettino della Società Paleontologica Italiana 32 (2): 145-264.

Stephensen K (1915) Isopoda, Tanaidacea, Cumacea, Amphipoda (excl. Hyperiidae). Report on the Danish Oceanographical expeditions 1908-1910 to the Mediterranean and adjacent Seas, 2, (Biology) D.1: 1-53, figs. 1-33.

Taning VA (1918) Mediterranean Scopelidae (Saurus, Aulopus, Chlorophthalmus and Myctophum). Danish Oceanographic Expedition IIA 7: 154 pp.

Tortonese E (1960) General Remarks on the Mediterranean Deep-sea Fishes. Bulletin De L'institut Oceanographique Monaco1167: 1-13.

Tselepides A, Lampadariou N, Hatziyanni E (2004) Distribution of meiobenthos at bathyal depths in the Mediterranean Sea. A comparison between sites of contrasting productivity. Sci Mar 68(3): 39-51.

Van Dover CL (2000) The Ecology of Deep-Sea Hydrothermal Vents. Princeton: Princeton University Press. 424 p.

Warwick RM, Howard HM, Somerfield PJ (1998) A synopsis of the freeliving marine nematodes. Part III: Monhysterids. Field Studies Council, Shrewsbury, 296.
