# Supplementary material for: Deep-Sea Biodiversity in the Mediterranean Sea: The Known, the Unknown, and the Unknowable
Source: PLoS One. 2010 Aug 2;5(8):e11832. doi: 10.1371/journal.pone.0011832 (PMC2914020; doi:10.1371/journal.pone.0011832)
Supplement: Text S2 — References included in the additional tables. (0.03 MB DOC) [file pone.0011832.s009.doc]

**Text S2: References included in the additional tables**

1. Luna GM, Dell’Anno A, Giuliano L, Danovaro R (2004) Bacterial diversity in deep Mediterranean sediments: relationship with the active bacterial fraction and substrate availability. Environ Microb 6: 745-753.
2. Polymenakou PN, Bertilsson S, Tselepides A, Stephanou EG (2005a) Links between geographic location, environmental factors and microbial community composition in sediments of the Eastern Mediterranean Sea. Microb Ecol 49: 367–378.
3. Polymenakou PN, Bertilsson S, Tselepides A, Stephanou EG (2005b) Bacterial community composition in different sediments from the Eastern Mediterranean Sea: a comparison of four 16S Ribosomal DNA clone libraries. Microb Ecol 50: 447–462.
4. Heijs SK, Laverman AM, Forney LJ, Hardoim PR, van Elsas JD (2008) Comparison of deep-sea sediment microbial communities in the Eastern Mediterranean. FEMS Microb Ecol 64(3): 362-377
5. Yakimov MM, Cappello S, Crisafi E, Tursi A, Savini A, et al. (2006) Phylogenetic survey of metabolically active microbial communities associated with the deep-sea coral Lophelia pertusa from the Apulian plateau, Central Mediterranean Sea. Deep Sea Res I 53: 62–75
6. Luna GM, Stumm K, Pusceddu A, Danovaro R (2009) Archaeal diversity in Deep-sea sediments estimated by means of different Terminal-Restriction Fragment Length Polymorphisms (T-RFLP) protocols. Curr Microb 59: 356–361
7. Danovaro R, Corinaldesi C, Luna GM, Magagnini M, Manini E, et al. (2009c) Prokaryote diversity and viral production in deep-sea sediments and Seamounts. Deep Sea Res II 56: 738-747.
8. Polymenakou PN, Lampadariou N, Mandalakis M, Tselepides A (2009) Phylogenetic diversity of sediment bacteria from the southern cretan margin, Eastern Mediterranean Sea. Syst Appl Microbiol 32: 17-26.
9. Parisi E (1981) Distribuzione dei foraminiferi bentonici nelle zone batiali del Tirreno e del Canale di Sicilia. Rivista Italiana di Paleontologia 87(2): 293-328.
10. Fontanier C, Jorissen FJ, Lansard B, Mouret A, Buscail R, et al. (2008). Live (stained) foraminiferal faunas from open slope environments separating submarine canyons in the Gulf of Lions (NW Mediterranean): diversity, density and microhabitats. Deep Sea Res I 55: 1532-1553
11. Schmiedl G, de Bovee F, Buscail R, Charrière B, Hemleben C, et al. (2000) Trophic control of benthic foraminiferal abundance and microhabitat in the bathyal Gulf of Lions, Western Mediterranean Sea. Mar Micropaleont 40: 167-188.
12. Heinz P, Kitazato H, Schmiedl G, Hemleben Ch (2001) Response of deep-sea benthic foraminifera from the Mediterranean Sea to simulated phytoplankton pulses under laboratory conditions. J Foraminif Res 31(3): 210–227.
13. Jannink NT (2001) Seasonality, biodiversity and microhabitats in benthic foraminifera. Geologica Ultraiectina 203 p. 191.
14. Jorrisen FJ (1988) The distribution of benthic foraminifera in the Adriatic Sea. Utrecht Micropaleontological Bulletins 37: 1-174.
15. De Stigter HC (1996) Recent and fossil benthic foraminifera in the Adriatic Sea: distribution patterns in relation to organic carbon flux and oxygen concentration at the Seabed. Geologica Ultraiectina 144: 254
16. De Rijk S, Jorissen FJ, Rohling EJ, Troelstra SR (2000) Organic flux on bathymetric zonation of Mediterranean benthic Foraminifera. Mar Micropaleont 40: 151-166.
17. Danovaro R, Bianchelli S, Gambi C, Mea M, Zeppilli D (2009) α-, β-, γ-, δ and ε-diversity of deep-sea nematodes in canyons and open slopes of the Northeast Atlantic and Mediterranean margins. Mar Ecol Progr Ser. 396: 197–209.
18. Danovaro R, Gambi C, Lampadariou N, Tselepides A (2008) Deep-sea nematode biodiversity in the Mediterranean basin: testing for longitudinal, bathymetric and energetic gradients. Ecography 31: 231-244.
19. Vivier MH (1978) Influence d’un déversement industriel profound sur la nématofaune (Canyon de Cassidaigne, Méditerranée). Téthys 8: 307-321
20. Soetaert K, Heip C, Vincx M (1991) Diversity of nematode assemblages along a Mediterranean deep-sea transect. Mar Ecol Progr Ser 75: 275-282.
21. Pusceddu A, Gambi C, Zeppilli D, Bianchelli S, Danovaro R (2009) Organic matter composition, meiofauna and nematode biodiversity in deep-sea sediments surrounding two Seamounts. Deep Sea Res II 56: 755-762.
22. Lampadariou N, Tselepides A (2006) Spatial variability of meiofaunal communities at areas of contrasting depth and productivity in the Aegean Sea (NE Mediterranean). Progr Oceanogr 69: 19-36.
23. Danovaro R, Dell’ Anno A, Fabiano M, Pusceddu A, Tselepides A (2001) Deep-sea ecosystem response to climate changes: the Eastern Mediterranean case study. Trends Ecol Evol 16: 505-510.
24. Ramirez-Llodra E, Company JB, Sardà F, Rotllant G (2009) Megabenthic diversity patterns and community structure of the Blanes submarine canyon and adjacent slope in the Northwestern Mediterranean: a human overprint? Mar Ecol 1-16.
25. Sardà F, Calafat A, Flexas MM, Tselepides A, Canals M, et al. (2004a). An introduction to Mediterranean deep-sea biology. Sci Mar 68 (3): 7-38.
26. Sardà F, D'Onghia G, Politou CY, Company JB, Maiorano P, Kapiris K (2004c) Maximum deep-sea distribution and ecological aspects of *Aristeus antennatus* (Risso 1816) in the Balearic and Ionian Mediterranean Sea. Sci Mar 68 (3): 117-127.
27. Sardà F, D'Onghia G, Politou C-Y, Tselepides A, eds (2004b) Mediterranean Deep-sea Biology. Monographs Scientia Marina 63 (3), 204 pp.
28. Tecchio S, Ramirez-Llodra E, Sardà F, Company JB. (2010) Biodiversity patterns of deep-sea benthic megafauna on western and central Mediterranean basins. Sci Mar.
29. Company JB, Maiorano A, Tselepides T, Politu CY, Plaity W, et al. (2004) Population characteristics of deep-sea decapod crustacean at four different sites of the Mediterranean Sea. Sci Mar 68( 3): 73-86.
30. Ramirez-Llodra E, Ballesteros M, Company JB, Dantart L, Sardà S (2008) Spatio-temporal variations of biomass and abundance in bathyal non-crustacean megafauna in the Catalan Sea (North-western Mediterranean). Mar Biol 153: 297-309.
31. Duperron S, de Beer D, Zbinden M, Boetius A, Schipani V, Kahil N, Gaill F (2009) Molecular characterization of bacteria associated with the trophosome and the tube of Lamellibrachia sp., a siboglinid annelid from cold seeps in the eastern Mediterranean. FEMS Microb Ecol 69(3): 395-409.
32. Duperron S, Halary S, Lorion J, Sibuet M, Gaill F (2008) Unexpected co-occurrence of six bacterial symbionts in the gills of the cold seep mussel Idas sp. (Bivalvia: Mytilidae). Environ Microb 10(2): 433-445.
33. Duperron S, Fiala-Médioni A, Caprais JC, Olu K, Sibuet M (2007) Evidence for chemoautotrophic symbiosis in a Mediterranean cold seep clam (Bivalvia: Lucinidae): comparative sequence analysis of bacterial 16S rRNA, APS reductase and RubisCO genes. FEMS Microb Ecol: 59 64–70.
34. Olu-Le Roy K, Sibuet M, Fiala-Médioni A, Gofas S, Salas C, et al (2004) Cold seep communities in the deep eastern Mediterranean Sea: composition, symbiosis and spatial distribution on mud volcanoes. Deep Sea Res I 51: 1915-1936.
